# Integrative proteomics and metabolomics data analysis exploring the mechanism of brain injury after cardiac surgery in chronic stress rats

**DOI:** 10.1186/s12871-024-02492-y

**Published:** 2024-03-22

**Authors:** Haoqi Yan, Hongbai Wang, Wenlin Chen, Yuan Jia, Fuxia Yan, Su Yuan

**Affiliations:** 1 Department of Anesthesiology, Fuwai Hospital, National Center of Cardiovascular Diseases, Chinese Academy of Medical Sciences and Peking Union Medical College, Beijing, 100037 People’s Republic of China; 2 Department of Cardiology, Peking Union Medical College Hospital, Peking Union Medical College & Chinese Academy of Medical Sciences, Beijing, China

**Keywords:** Chronic stress, Cardiac surgery, Brain injury, Metabolomics, Proteomics

## Abstract

**Objective:**

Preoperative chronic stress (CS) is associated with postoperative brain injury in patients undergoing open heart cardiac surgery. This research is to explore the potential molecular biological mechanisms of brain damage following cardiac surgery in preoperative CS rats by the analyses combining proteomics and metabolomics.

**Methods:**

We constructed the chronic unpredictable stress (CUS) and cardiac surgery models in adult rats. We proved the brain injury in CUS cardiac surgery rats by Hematoxylin–Eosin (H&E) staining, followed by separating the hippocampal tissue and investigating the potential mechanisms of brain injury by the methods of data-independent acquisition proteomics and untargeted metabolomics.

**Results:**

The signaling pathways of glycoproteins and metabolism of amino acids were the main possible mechanisms of brain injury in CUS rats following cardiac surgery according to the proteomics and metabolomics. In addition, the pathways of animo acids metabolism such as the pathways of lysine degradation and β-alanine metabolism may be the main mechanism of cardiac surgery related brain injury in preoperative CUS rats.

**Conclusions:**

The pathways of animo acids metabolism such as lysine degradation and β-alanine metabolism may be the potential mechanisms of brain injury in CUS rats following cardiac surgery. We should focus on the varieties of bioproteins and metabolites in these pathways, and related changes in other signaling pathways induced by the two pathways.

**Supplementary Information:**

The online version contains supplementary material available at 10.1186/s12871-024-02492-y.

## Introduction

Brain injury is a serious complication after cardiac surgery. Brain injury can have a variety of clinical manifestations. Previous studies have defined two types of poor neurological outcomes after coronary artery bypass grafting (CABG). Type I outcome includes fatal or non-fatal stroke or coma at discharge; type II outcome includes cognitive function deterioration, memory deficit, or seizures [1]. As a type II outcome, postoperative delirium (POD) is an acute, fluctuating psychiatric disorder following surgeries, and its main clinical manifestations include reduced ability to attention, disorganized thinking, and defect of spatial orientation [2]. POD is prevalent in patients undergoing cardiac and major non-cardiac surgeries and usually occurs in the first 3 days after surgery [3, 4]. According to previous studies, the incidence of POD ranges from 5%-72% in cardiac surgery patients [5, 6]. POD negatively affects postoperative recovery and is associated with a high incidence of long-term cognitive dysfunction and even death [7–9]. Although the specific mechanism of POD currently remains unclear, postoperative brain injury has been identified in patients with POD [10]. Cardiac surgery has been shown to compromise hippocampal neural networks, contributing to the development of delirium [11]. In addition, a secondary analysis from a randomized trial demonstrated that several cerebral injury biomarkers in the plasma peaked two hours post-cardiopulmonary bypass during cardiac surgery [12]. Besides, some studies have demonstrated that preoperative chronic stress (CS) is the main risk factor associated with POD [13–15]. Additionally, patients with cardiac disease easily develop CS symptoms such as anxiety and insomnia [16, 17]. Therefore, we hypothesize that the patient with preoperative CS may be more prone to postoperative brain injury through some molecular biological mechanisms in cognition-related brain regions, thereby resulting in POD occurrence. The objective of this experiment is to explore the possible mechanisms of injury in the hippocampal region of the brain following cardiac surgery in preoperative chronic unpredictable stress (CUS) rats using proteomics and metabolomics.

## Materials and methods

### Animals

We selected male Sprague–Dawley (SD) rats aged 7–8 weeks old and weighing 250 ± 20 g (the Animal Center of Fuwai Hospital). All experimental protocols were approved by Fuwai Hospital Animal Experimental Committee. All experimental rats were housed three per cage under the environment 22 °C with a 12-h light/dark cycle and had ad libitum access to food and water for 7 d to adapt to the new environment.

### Hematoxylin–eosin (HE) staining

The brain tissues of rats (Procedure A) on the day 22 were collected, fixed in 4% paraformaldehyde, embedded in paraffin, and dissected into sections with 10 μm thickness. Each section was then deparaffinized, hydrated, washed, and stained with hematoxylin–eosin (H&E) using a commercially purchased kit (Beyotime, Shanghai, China).

### Experimental design

**Fig. 1:**
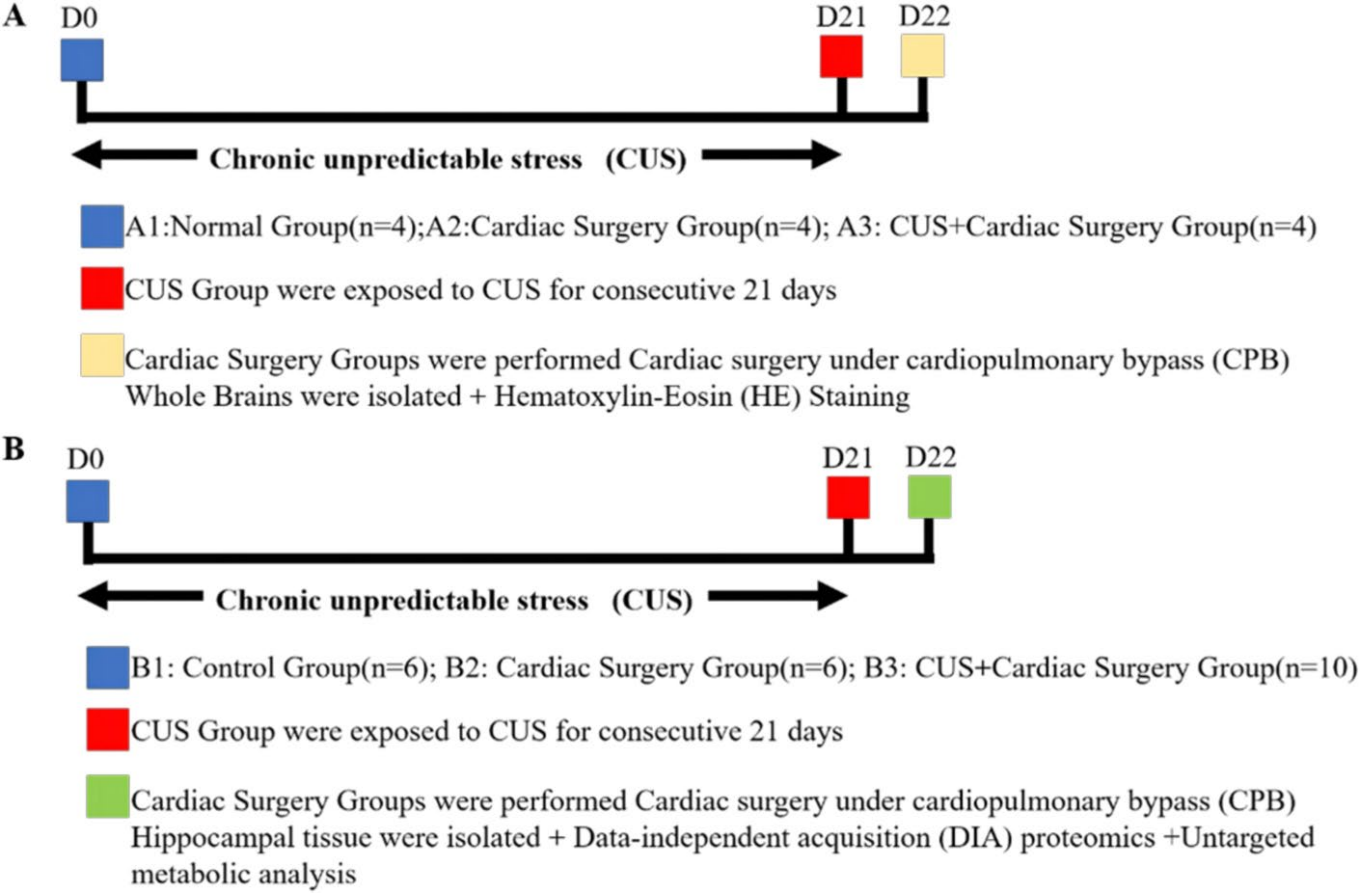
Experiment Timeline (**A**) Procedure A (**B**) Procedure B

In the current study, all rats were randomly assigned into two procedures after 7 days adaptation (Fig. 1). In Procedure A, the rats were divided into three groups.1) Normal(A1) group (*n* = 4): non-CUS and non-cardiac surgery; 2) cardiac surgery (A2) group (*n* = 4): non-CUS but cardiac surgery; and 3) CUS and cardiac surgery (A3) group (*n* = 4). As the Fig. 1B shows, the rats were randomly divided into three groups in procedure B.1) control (B1) group (*n* = 6): non-CUS and non-cardiac surgery; 2) cardiac surgery (B2) group (*n* = 6): non-CUS but cardiac surgery; and 3) CUS and cardiac surgery (B3) group (*n* = 10): CUS and cardiac surgery. The objective of establishing the cardiac surgery group is to eliminate the impact of acute stress induced by cardiac surgery on the rats. Meanwhile, the purpose of the control group is to mitigate the influence of confounding factors on the experiment. The rats assigned to the groups of non-CUS were kept undisturbed in their own cages, while rats assigned to the CUS group were exposed to chronic unpredictable stress for 21 days. The weights of rats were meticulously recorded on day 0, 7, 14, and 21. A comparative analysis of weight, using the Welch t-test, was conducted on days 0 and 21 between rats exposed to CUS and those in the non-CUS group to confirm the stress level of CUS rats [18]. On day 22, We performed cardiac surgery on cardiac surgery groups and decapitated all rats immediately after surgery. We isolated the whole rats’ brain for HE staining in procedure A and isolated the rats’ hippocampus for data-independent acquisition (DIA) and untargeted metabolomics in procedure B.

### Chronic unpredictable stress (CUS) model

Rats assigned to the CUS groups were exposed to two of the following eight stressors daily for consecutive 21 days: 1) 40 Hz 90 dB noise stimulation for 9 min; 2) swimming for 5 min in 4℃ cold water; 3) body restraint for 1 h; 4) pinching tail for 1 min; 5) food deprivation for 24 h; 6) water deprivation for 24 h; 7) nyctohemeral rhythm inversion, and 8) cage tilt at 45º [19]. To prevent the rats from adapting to the stress factors, we exerted two different stress factors on them daily, and the stressors varied within 48 h [19]. We performed the cardiac surgery under cardiopulmonary bypass (CPB) on day 22.

### Cardiac surgery model under cardiopulmonary bypass (CPB)

According to the surgical protocol from Koning et al. [20], the rats were anesthetically induced by 5% isoflurane inhalation and had no foot reduction response under pain stimulation, followed by endotracheal intubation (16G), and mechanical ventilation (tidal volume: 10 ml/kg, respiratory rate: 60–65 breaths/min, peep 2-4cmH2O). 2–3% isoflurane was continuously inhaled to maintain anesthesia and intraoperative inhaled oxygen concentration was 35%. The body temperature was maintained at 36.5℃ except during extracorporeal circulation. The blood pressure of the tail artery was monitored with a 22-G cannula needle. Fentanyl was 12 μg/kg, the intravenous injection was repeated before the start of CPB and 40 min after CPB, and inhaled isoflurane concentration was reduced to 1.5–2% when fentanyl was injected. The main components of CPB include a venous blood reservoir, a transfer pump, and a 4 ml oxygenator heat exchanger with three layers of hollow fiber membrane. Pipe through the right femoral artery and right jugular vein, and the end of the right jugular vein reaches the right atrium. After 500 U / kg of intravenous heparin, CPB was initiated and lasted for 30 min. The heart was exposed during CPB. Blood gas and hemoglobin concentrations were monitored at 10 min and 30 min after the start of CPB, and at 10 min and 60 min after the end of CPB, respectively. The pump liquid of CPB was 10 ml of 6% hydroxyethyl starch with a flow rate of 150–200 ml/kg/ min. The body temperature was maintained at the level of mild body hypothermia (35℃) during CPB and rewarmed slowly to 36–36.5℃, and then CPB was halted. Mechanical ventilation was restarted 10 min before the end of CPB. Heparin was neutralized by 2 mg/kg of protamine within 15 min after the end of CPB.

Immediately after surgery, when is the most severe brain injury occurs [12], all rats were decapitated, and the whole hippocampal tissue of the brain was isolated and frozen in liquid nitrogen, and then transferred to the fridge at -80 ℃.

### Data-independent acquisition (DIA) proteomics analysis

#### Total protein extraction and sample preparation

Hippocampal tissue (Procedure B) was ground individually in liquid nitrogen and lysed with lysis buffer containing 8 M Urea, 100 mM NH4HCO3 (pH 8), and 0.2% SDS, followed by 5 min of ultrasonication on ice. The lysate was centrifuged at 12000 g for 15 min under the 4℃ environment and the supernatant was transferred to a clean tube. Extracts from each sample were reduced with 10 mM DTT for 1 h at 56℃ and subsequently alkylated with sufficient iodoacetamide for 1 h at room temperature in the dark. Then samples were completely mixed with 4 times the volume of precooled acetone by vortexing and incubated at -20℃ for 2 h or more. Samples were then centrifuged, and the precipitation was collected. After washing twice with cold acetone, the pellet was dissolved by dissolution buffer, which contained 0.1 M triethylammonium bicarbonate (TEAB, pH 8.5) and 6 M urea.

To perform protein quality test, the BSA standard protein solution was prepared according to the instructions of Bradford protein quantitative kit, with gradient concentration ranged from 0 to 0.5 g/L. The standard curve was drawn with the absorbance of standard protein solution and the protein concentration of the sample was calculated. Then 120 μg of each protein sample was taken and the volume was made up to 100 μL with dissolution buffer, 1.5 μg trypsin and 500 μL of 100 mM TEAB buffer were added, after one night digestion, supernatant was collected and washed with washing buffer for 3 times,the eluent of each sample were combined and lyophilized.

#### LC–MS/MS analysis and data analysis

According to the protocol provided by Novogene,the sample was fractionated using a C18 column (Waters BEH C18 4.6 × 250 mm, 5 μm) on a Rigol L-3000 HPLC system. Proteomics analyses were conducted utilizing the EASY-nLC™ 1200 UHPLC system (Thermo Fisher), coupled with a Q Exactive HF-X mass spectrometer (Thermo Fisher). The instrument operated in both data-dependent acquisition (DDA) mode and data-independent acquisition (DIA) mode, each applied separately.

The protein database employed Proteome Discoverer 2.4 (PD2.4, Thermo) to analyze data obtained through DDA scanning. Key search parameters included a 10 ppm mass tolerance for precursor ions and a 0.02 Da tolerance for fragment ions. Fixed modifications comprised cysteine alkylation, while variable modifications encompassed methionine oxidation and N-terminus acetylation, allowing for up to 2 missed cleavage sites. To improve analysis quality, PD2.4 filtered results, retaining only peptide spectrum matches (PSMs) with a confidence level exceeding 99% and proteins with at least one unique peptide segment. False discovery rate (FDR) validation removed PSMs and proteins with FDR greater than 1%.

Identification outcomes from PD2.4 were imported into Spectronaut (version 9.0, Biognosys) to generate a spectral library. Peptide and ion pair selection rules were applied [21], choosing peptides and sub-ions meeting criteria to form a target list. DIA data was then imported, with chromatographic peaks extracted based on the target list. Sub-ion matching and peak area calculations enabled simultaneous qualitative and quantitative analysis of peptide segments. Retention time calibration used standard peptides in the samples, and a precursor ion Q-value cutoff was set at 0.01.

#### Differential protein analysis

The identified proteins were annotated by the databases of Cluster of Orthologous Groups of proteins (COG), Gene Ontology (GO), KEGG (Kyoto Encyclopedia of Genes and Genomes), and IPR [22]. Quantitative analyses of proteins included total differential analysis of identified proteins, screening and expression pattern clustering analysis of the differential proteins. The progress of protein differential analysis included: 1) picking out the sample pairs for comparison; 2) calculating fold change (FC): the ratio of the mean of all biological repeat quantitative values of each protein in the comparison sample pair. To determine the significance of the difference, a Welch t test of relative quantitative values of each protein in the sample and the corresponding *p*-value was calculated as a significance indicator. Up-regulation protein was screened when the *p*-value <0.05, meanwhile FC 1.2 or more, while down-regulation was screened when the *p*-value <0.05, meanwhile FC 0.83 or less based on previous research [23, 24]. After that, we conducted GO and KEGG functional enrichment analysis to observe the function of selected differential proteins [25].Then, we performed subcellular localization analysis to examine the spatial expression patterns of differentially expressed proteins (DEPs).Finally, the probable protein–protein interaction were predicted using the STRING-db server [26].

### Untargeted metabolomics

#### Sample preparation

Samples (100 mg each) were extracted and grounded with liquid nitrogen individually, and the homogenate was resuspended by a well vortex on ice with precooled buffer (80% methanol and 0.1% formic acid). The supernatant of the lysate was diluted to the final concentration by LC–MS grade water after centrifugation at 15,000 g at 4 °C for 20 min. The samples were transferred into a new tube and were centrifuged at 15,000 g at 4 °C for 20 min, and the supernatant was subsequently injected into the LC–MS/MS system analysis [27].

#### UHPLC-MS/MS analysis and data processing

Samples were loaded using a Hypesil Gold column (100 × 2.1 mm, 1.9 μm) on a Vanquish UHPLC system (Thermo Fisher, Germany) coupled with an Orbitrap Q ExactiveTMHF-X mass spectrometer (Thermo Fisher, Germany) in Novogene Co., Ltd. The stated linear gradient was 17 min and the flow rate was 0.2 ml/min.

For the data process, the raw data files generated by UHPLC-MS/MS were processed using the Compound Discoverer 3.1 (CD3.1, ThermoFisher) to perform peak alignment, peak picking, and quantitation for each metabolite. The primary parameters were configured as follows: a retention time tolerance of 0.2 min, an actual mass tolerance of 5 ppm, a signal intensity tolerance of 30%, a signal-to-noise ratio of 3, and a minimum intensity threshold of 100,000. After that, peak intensities were normalized to the total spectral intensity. Following this, the intensities of peaks were adjusted to the total spectral intensity. The resulting normalized data was then employed to forecast the molecular formula using information from additive ions, molecular ion peaks, and fragment ions. And then peaks were matched with the mzCloud (https://ww.mzcloud.org/). mzVault and MassList database to obtain the accurate qualitative and relative quantitative results. Statistical analyses were conducted utilizing R (version R-3.4.3), Python (version 2.7.6), and CentOS (CentOS release 6.6). In cases where the data deviated from a normal distribution, efforts were made to normalize them using the area normalization method.

#### Differential metabolites analysis

For the pathway analysis, metabolites were annotated by the KEGG database, Human Metabolome Database (HMDB), and LIPID Maps database. Principal components analysis (PCA) and partial least squares discriminant analysis (PLS-DA) were performed using metaX. Univariate analysis (Welch t test) was applied to evaluate the statistical significance. The metabolites with Variable Importance in the Projection (VIP) > 1 and *p*-value < 0.05 and FC ≥ 1.2 or FC ≤ 0.83 were considered to be differentially expressed metabolites based on previous research [28–30]. After that, the correlation between differential metabolites were analyzed using R (version R-3.4.3). Finally, to gain an overview of differentially expressed metabolites, the functions of these metabolites and metabolic pathways were studied by KEGG analysis.

### Integrated analysis of proteomics and metabolomics

To explore the correlation between differentially expressed proteins and metabolites, we performed the enrichment analysis of common pathways of differentially expressed proteins and metabolites by combining the proteomic and metabolomic analytical results. The relevance between differentially expressed proteins and metabolites was analyzed using the Pearson test. To obtain an overview of the common pathways, a KEGG analysis was conducted.

## Results

### Bodyweight

All animals survived throughout the experiment. At the beginning of the experiment, the mean body weight of all rats was 330.97 g, with no significant difference between CUS rats and no-CUS rats (*P* > 0.05). At 21 days after CUS exposure, a reduction in weight became apparent in CUS rats (*P* < 0.001), (Supplement Fig. 1). These findings confirm the stress level in rats undergoing the CUS model.

### Histopathological changes in the Hippocampus(HE Staining)

**Fig. 2:**
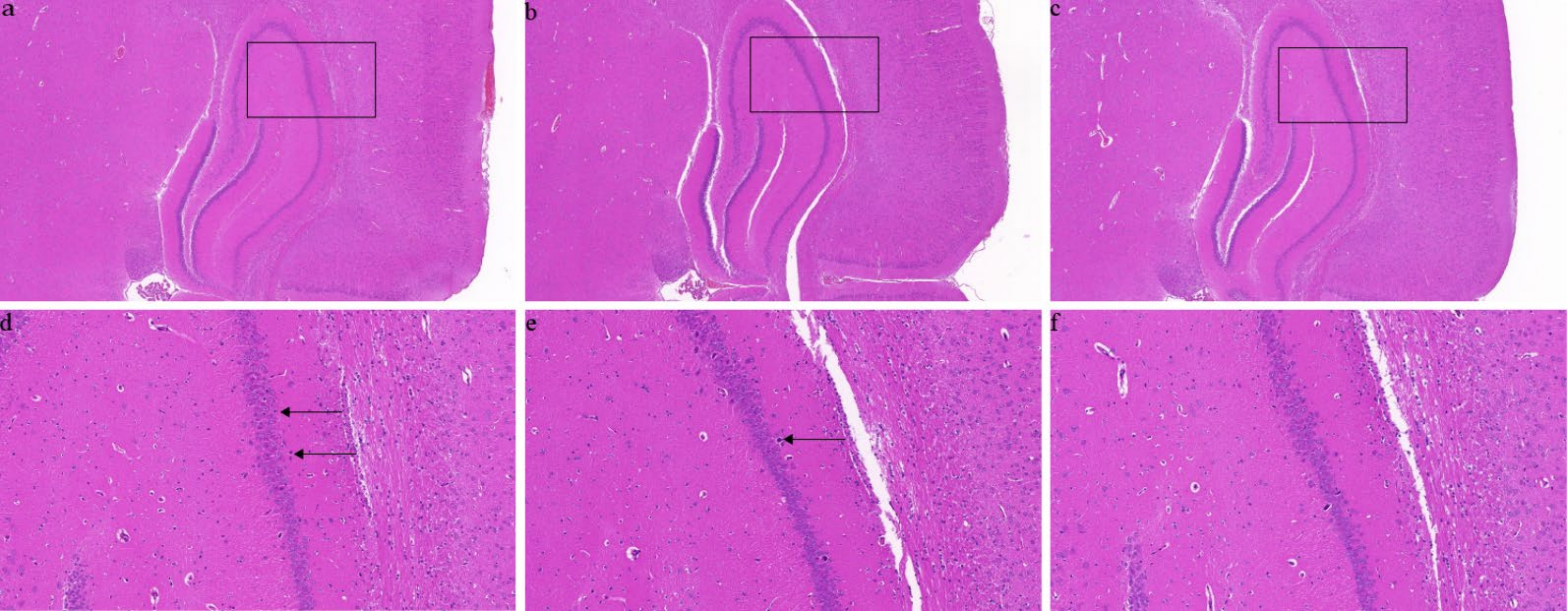
Histopathological changes in hippocampus tissues of rats in each group were observed by HE staining. The hippocampus tissues of CUS and cardiac surgery rats, at fivefold magnification (**a**) and at 20 fold magnification (**d**) after HE staining, the black arrow indicates the degenerative neurons. The hippocampus tissues of cardiac surgery rats, at fivefold magnification (**b**) and at 20 fold magnification (**e**) after HE staining, the black arrow indicates the degenerative neurons. The hippocampus tissues of normal rats, at fivefold magnification (**c**) and at 20 fold magnification (**f**) after HE staining

As shown in Fig. 2 and Supplement Fig. 2A-C, compared with normal group, the cardiac surgery group had mild brain damage, while hippocampal neurons in the CUS and cardiac surgery group were degenerated compared with cardiac surgery group, indicating the chronic stress can aggravate the brain injury of cardiac surgery in rats.

### Data-independent acquisition (DIA) proteomics analysis

#### Overview of proteomics analysis data

Altogether 402573 spectra were identified. A total of 234913matched spectra and 78937 unique peptides were obtained after the data filtration. Furthermore, 8392 proteins with FDR (false discovery rate) ≤ 0.01 were identified. After calibration with standard peptides, 7377 proteins were screened out for further comparative analysis. In addition, a total of 3425, 5685, 7362, and 7221 functional proteins were annotated by the four databases of COG, GO, KEGG, and IPR, respectively.

### Differentially expressed proteins (DEPs) analysis

**Fig. 3:**
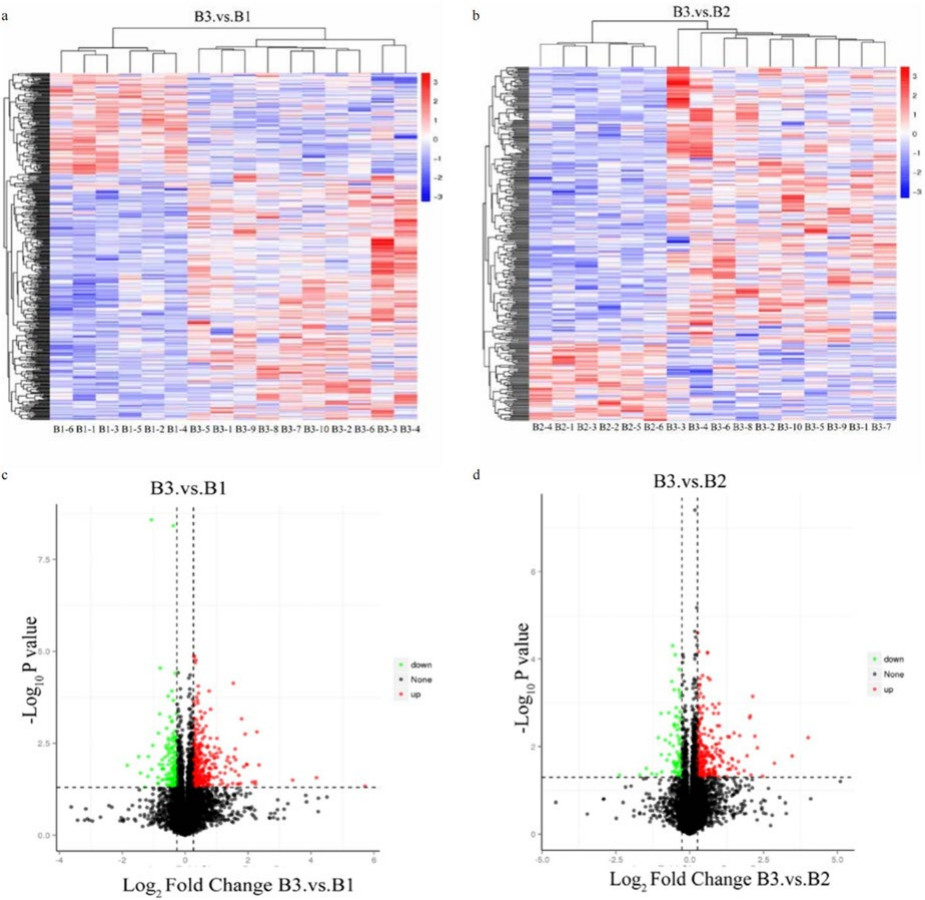
Heatmaps of all up-regulated and down-regulated DEPs between (**a**) CUS cardiac surgery (B3) and control group (B1); (**b**) CUS cardiac surgery (B3) and cardiac surgery group (B2); Volcano plots of all up-regulated and down-regulated DEPs between (**c**) CUS cardiac surgery (B3) and control group (B1); (**d**) CUS cardiac surgery (B3) and cardiac surgery group (B2)

DEPs analysis was conducted between different treatment groups. The volcano plots and heatmaps were applied to illustrate the discrepancy. We analyzed the protein expression differences between CUS cardiac surgery group (B3) and control group (B1) (Fig. 3a, c), CUS cardiac surgery group (B3) and cardiac surgery group (B2) (Fig. 3b, d). According to the criteria of *p* value < 0.05 and fold changes  ≥ 1.2 or ≤ 0.83, there were 333 proteins up-regulated, and 141 proteins downregulated between the B3 and B1 group, while 257 proteins were up-regulated, and 77 proteins were downregulated between the B3 and B2 groups.

**Fig. 4:**
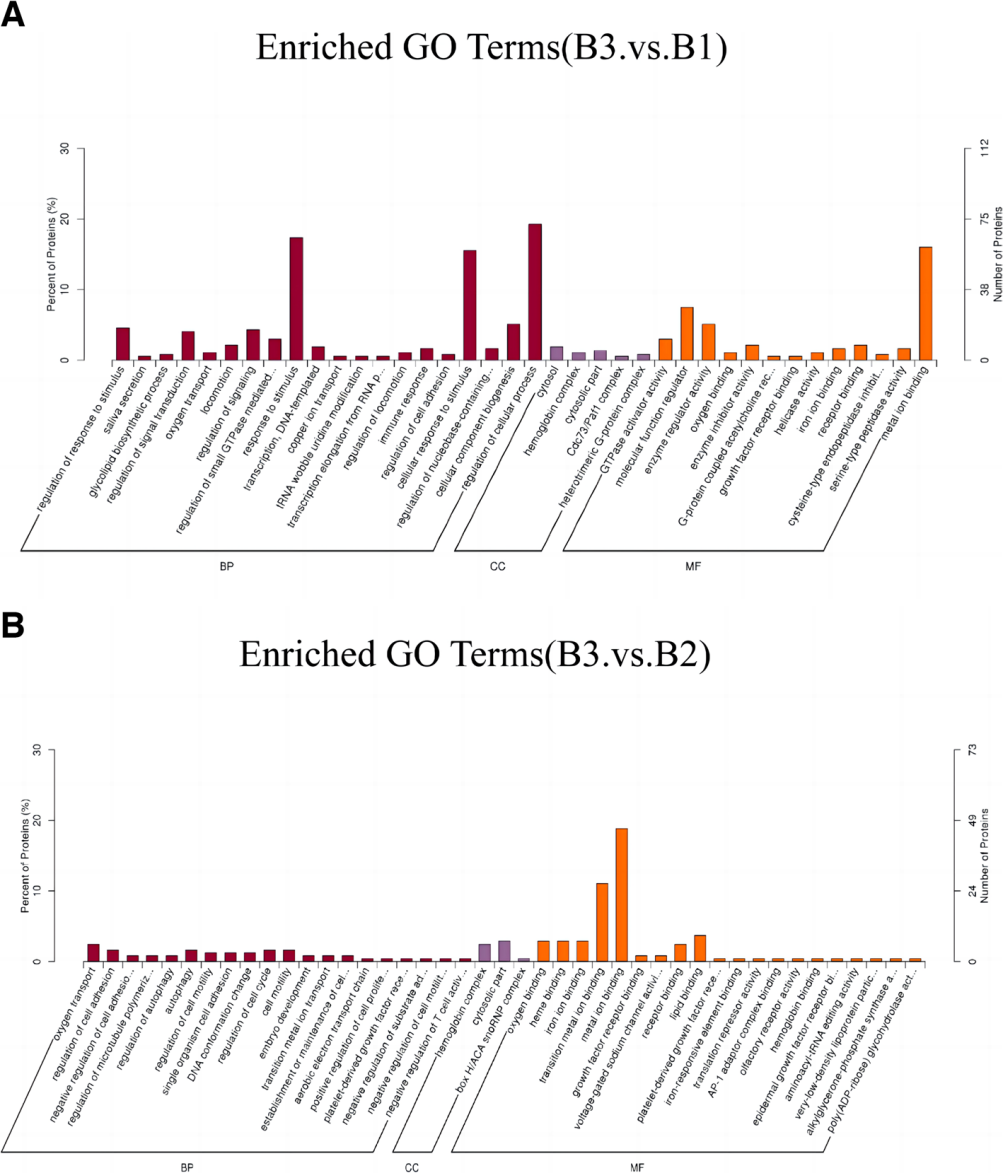
**A** Gene Ontology (GO) analysis among the DEPs between CUS cardiac surgery (B3) and control group (B1);BP: biological process; CC: cellular component; MF: molecular function.** B** Gene Ontology (GO) analysis among the DEPs between CUS cardiac surgery (B3) and cardiac surgery group (B2); BP: biological process; CC: cellular component; MF: molecular function

Generally, according to the proteomics analysis, the signaling pathways of glycoproteins were the main possible mechanisms of brain injury in CUS rats following cardiac surgery. In GO enrichment analysis, between the control group and the CUS cardiac surgery group, DEPs were enriched in the regulation of cellular process and cellular response to stimulus (Fig. 4A). Between the CUS cardiac surgery group and the cardiac surgery group, DEPs were enriched in metal ion binding, transition ion binding, lipid binding (Fig. 4B), demonstrating chronic stress influences neurotransmitter transmission process and molecular function in hippocampus tissue.

**Fig.5:**
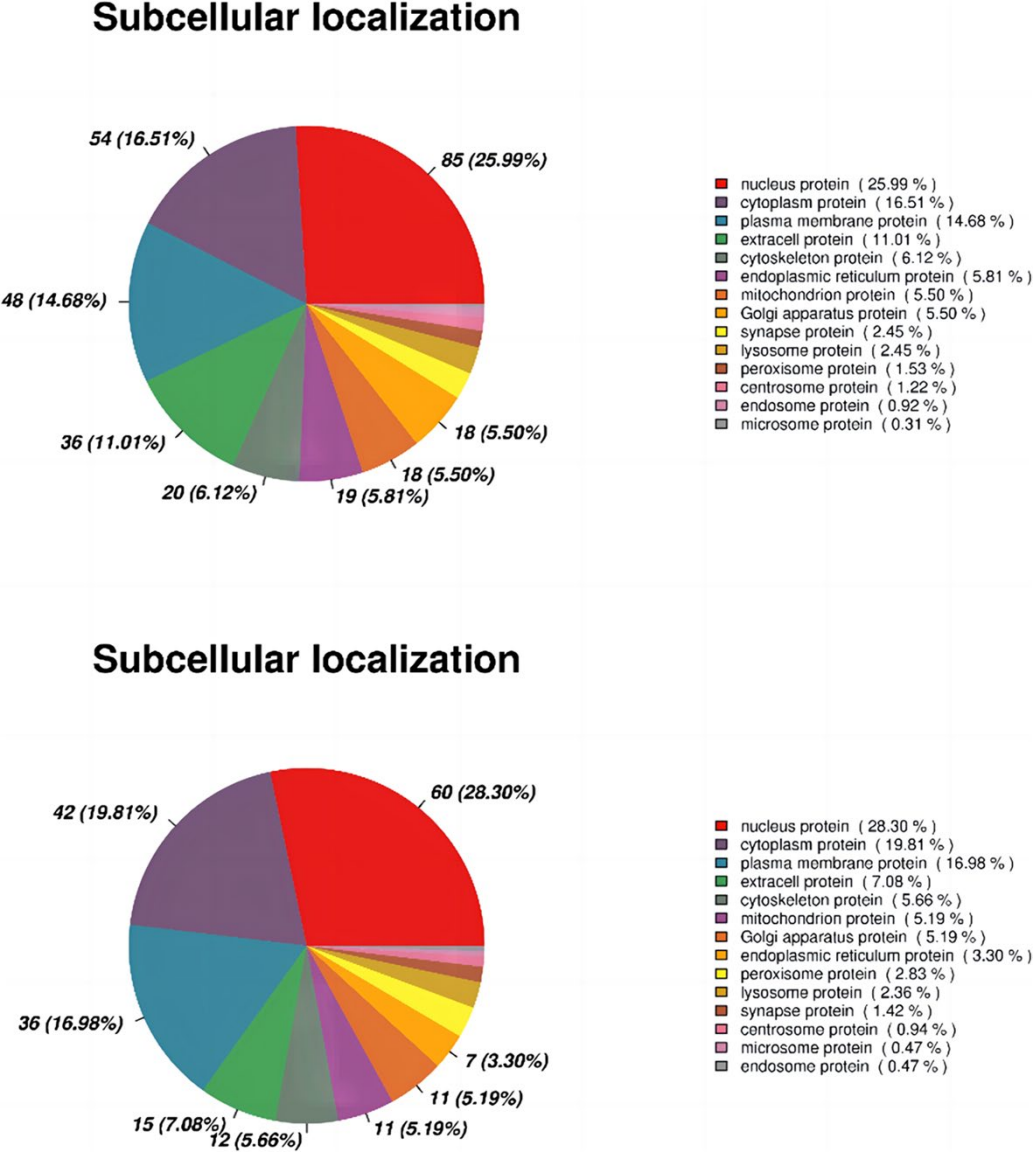
**A** Subcellular localization between CUS cardiac surgery and control group. **B** Subcellular localization between CUS cardiac surgery and cardiac surgery group

To examine the spatial expression patterns of differentially expressed proteins (DEPs), we performed subcellular localization analysis. The nucleus, cytoplasm, and plasma membrane emerged as the three predominant locations for DEPs in both the CUS cardiac surgery and control groups, as well as the CUS cardiac surgery and cardiac surgery groups (Fig. 5A, B).

Furthermore, we used the StringDB protein interaction database (http://string-db.org/) for the interaction analysis of identified proteins. The connections of DEPs between comparison groups were exhibited and the up-regulation, down-regulation, and interaction were explored (Supplement Fig. 3A,B).

## Metabolomics analysis

### Overview of metabolomics analysis data

Metabolomics analysis was performed to analyze the phenotypic changes. After deducting the isotope peaks, 535 ions in ESI+ and 386 ions in ESI- mode were detected. The PCA analysis of ions in both ESI+ and ESI- mode revealed the unique features and distribution of four different treated groups.

The functional and taxonomic annotation was further conducted. KEGG analysis suggested the tendency of the ions to enrich general metabolism pathways. HMDB annotation indicated that ions were generally involved in organic acid and derivatives, lipids and lipid-like molecules, and organ heterocyclic compounds. LIPID Maps annotation indicated that flavonoids (polyketides), glycerophoethanolamines (glycerophospholipids), and steroids (sterol lipids) were the main compounds in ions in ESI+ mode, and glycerophosphocholines (glycerophospholipids), fatty acids and conjugates were the majority of ions in ESI- mode due to their quantity of electricity.

### Differentially expressed metabolites (DEMs) analysis

**Fig. 6:**
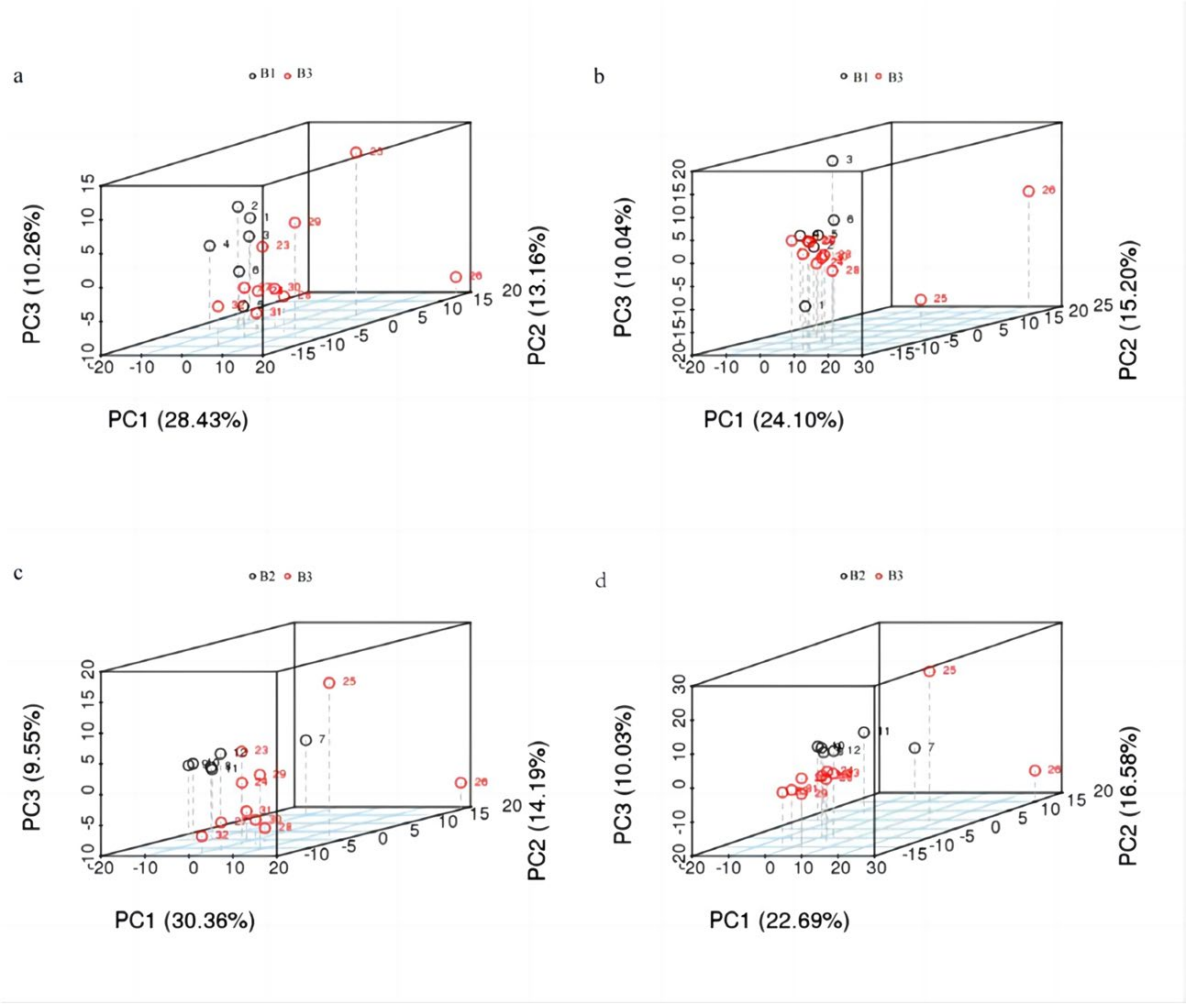
PCA analysis between (**a**) B3 and B1 group (ESI- mode) (**b**) B3 and B1 group(ESI+ mode) (**c**) B3 and B2 group(ESI- mode) (**d**) B3 and B2 group(ESI+ mode)

PCA and PLS-DA were employed to downscale the multidimensional data and realize regression analysis, and then screen out the DEMs. The relative quantitative values of the differential metabolites were normalized and clustered, while the PCA results are shown in the (Fig. 6).

**Fig. 7:**
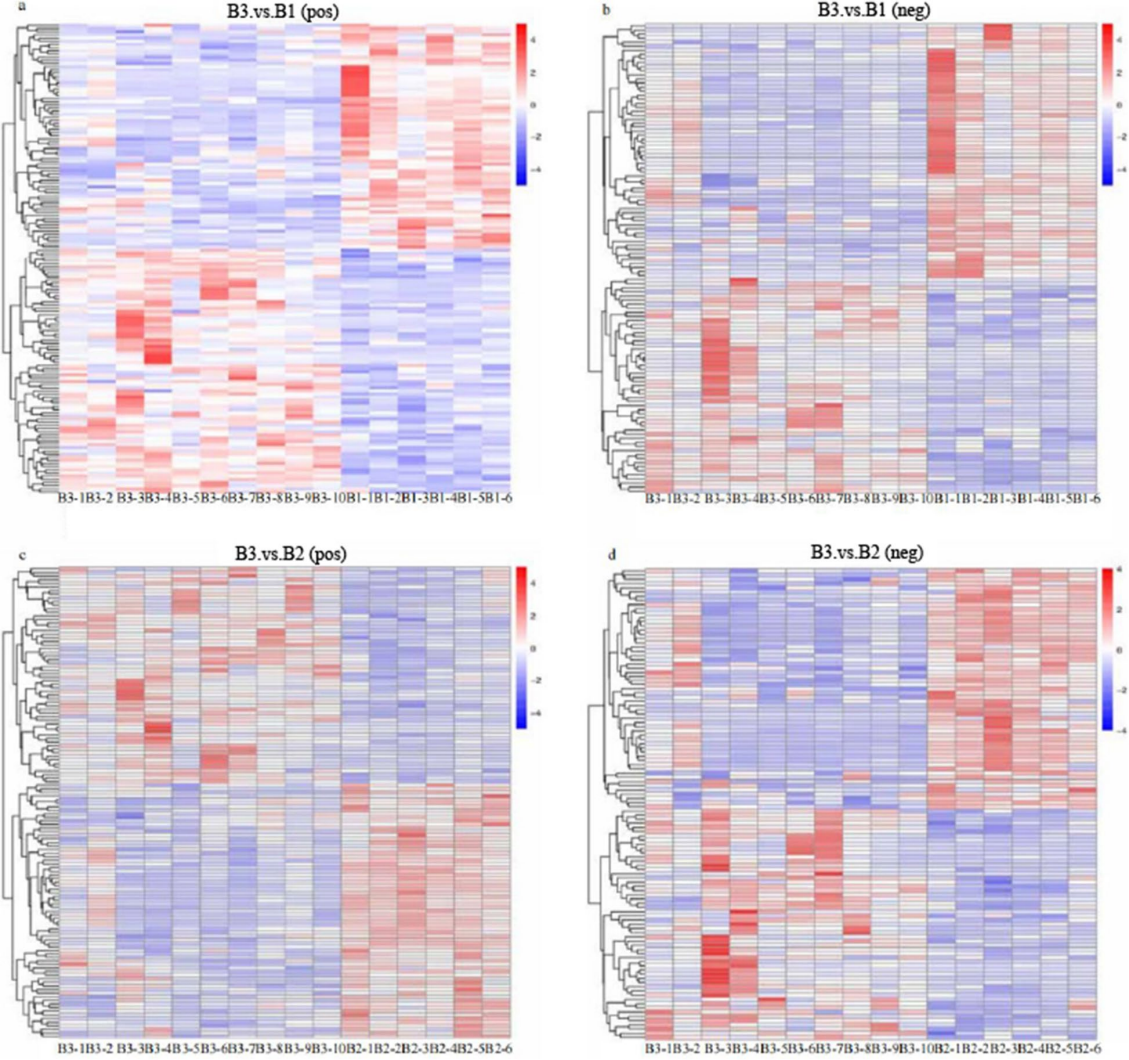
(**a**)Heatmaps of all up-regulated and down-regulated DEPs between CUS cardiac surgery (B3) and control group (B1) (ESI+);(**b**) CUS cardiac surgery (B3) and control group (B1) (ESI-);(**c**) CUS cardiac surgery (B3) and cardiac surgery group (B2) (ESI+);(**d**) CUS cardiac surgery (B3) and cardiac surgery group (B2) (ESI-)

The analysis of DEMs were conducted between different treatment groups. The heatmaps were applied to illustrate the discrepancy, and the DEMs were 149 ions in ESI+ and 116 ions in ESI- mode between CUS cardiac surgery group and control group (Fig. 7a, b), while the DEMs were 131 ions in ESI+ and 112 ions in ESI- mode in comparison to the CUS cardiac surgery and cardiac surgery group (Fig. 7c,d).

To explore the consistency and correlation between different metabolites, we performed a differential metabolite correlation analysis. The results of differentially expressed ions in ESI+ and ESI- group between CUS cardiac surgery group and control group were demonstrated in the Supplement Fig. 4A, B, Many differential metabolites exhibit both positive and negative correlations with each other. For example, Gly-Phe is negatively correlated with 2,3,4-Trihydroxybenzoic acid, while is positively correlated with 2-Arachidonoyl glycerol. The Supplement Fig. 4C, D illustrates the outcomes of differentially expressed ions within both ESI+ and ESI-group when comparing the CUS cardiac surgery group with the cardiac surgery group. Lipid metabolic products exhibit pairwise positive correlations with each other, while some differential metabolites exhibit negative correlation with each other, such as LPE 18:0 and N-carbamyl-L-glutamicacid.

**Fig. 8:**
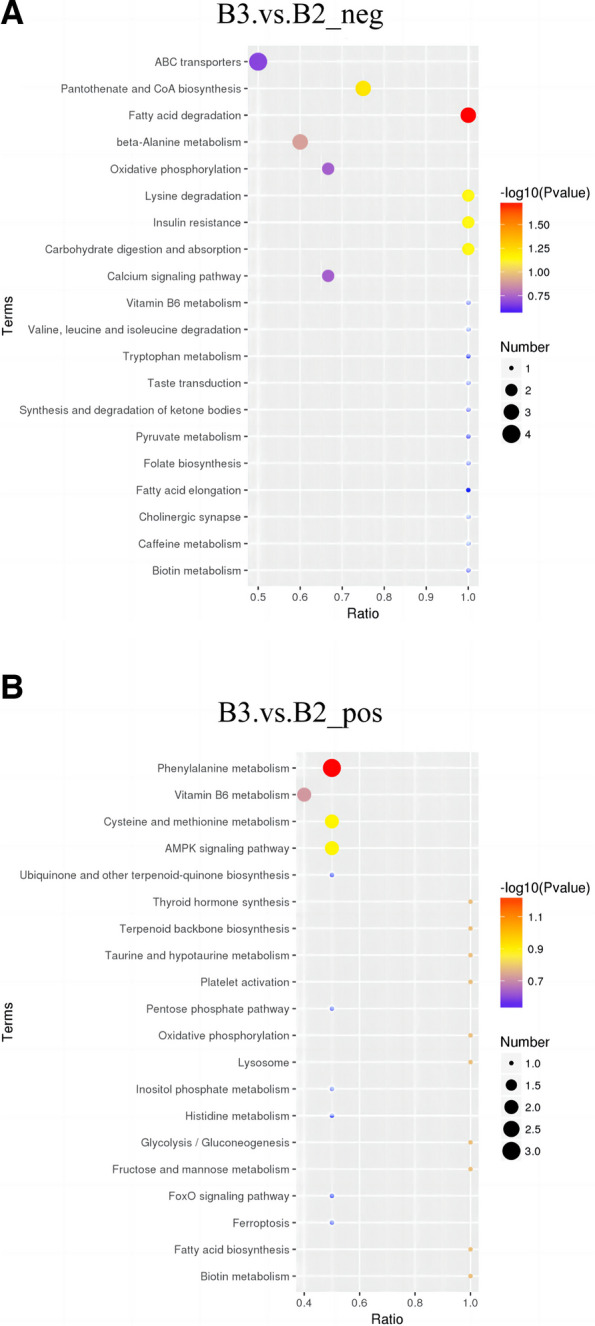
**A** Gene set enrichment analysis of KEGG analysis among the DEMs in ESI- mode between CUS cardiac surgery (B3) and cardiac surgery group (B2). **B** Gene set enrichment analysis of KEGG analysis among the DEMs in ESI+ mode between CUS cardiac surgery (B3) and cardiac surgery group (B2)

In KEGG enrichment analysis, we found that the metabolism of amino acids and lipids were the main possible mechanisms of brain injury in CUS rats following cardiac surgery. Analysis of CUS cardiac surgery and cardiac surgery group indicated that ions ESI+ DEMs were enriched in phenylalanine metabolism, vitamin B6 metabolism, cysteine, and methionine metabolism, AMPK signaling pathway, while ions ESI- DEMs were enriched in ABC transporters, lysine degradation and beta-alanine metabolism pathway (Fig. 8A, B), suggesting CUS influences cell function by changing the lipid metabolism and phenylalanine metabolism.

To further evaluate whether the DEMs could be potential diagnostic biomarkers, we performed ROC analysis with the quantitative values of DEMs and presented them as ROC curves. The results suggested that the DEMs all had good predictive properties. Specifically, the AUC of the ROC curve is 0.95 in the CUS cardiac surgery group and control group, indicating the DEMs have a great predictive property in cardiac surgery. Differential metabolites with diagnostic significance can be further clarified the neurological prognosis and adverse events after cardiac surgery (Supplement Fig. 5).

### Integrated analysis of metabolomics and proteomics

**Fig. 9:**
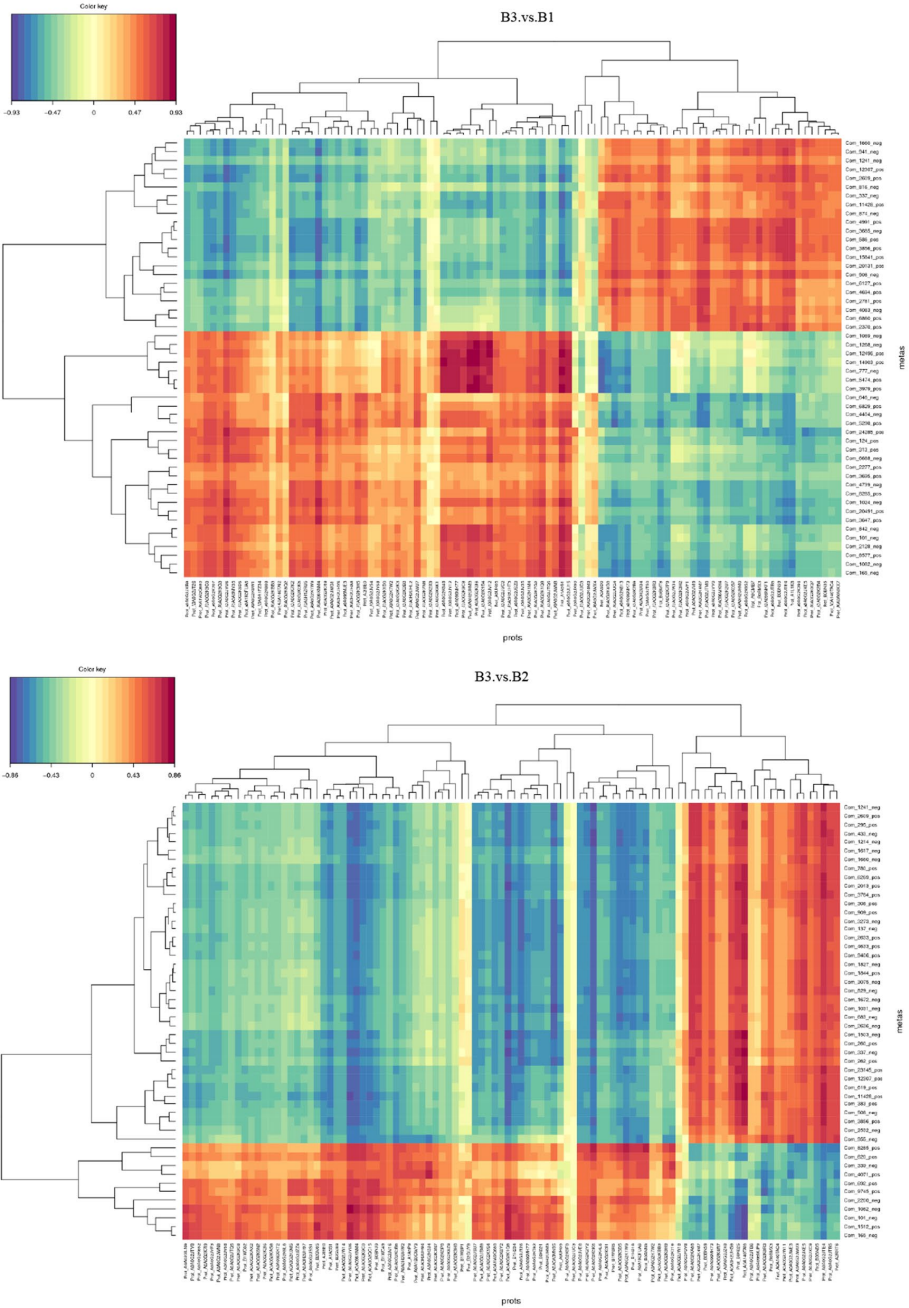
**A** Heatmaps of correlation analysis among the DEPs and DEMs between CUS cardiac surgery (B3) and control group (B1). **B** Heatmaps of correlation analysis among the DEPs and DEMs between CUS cardiac surgery (B3) and cardiac surgery group (B2)

**Fig. 10:**
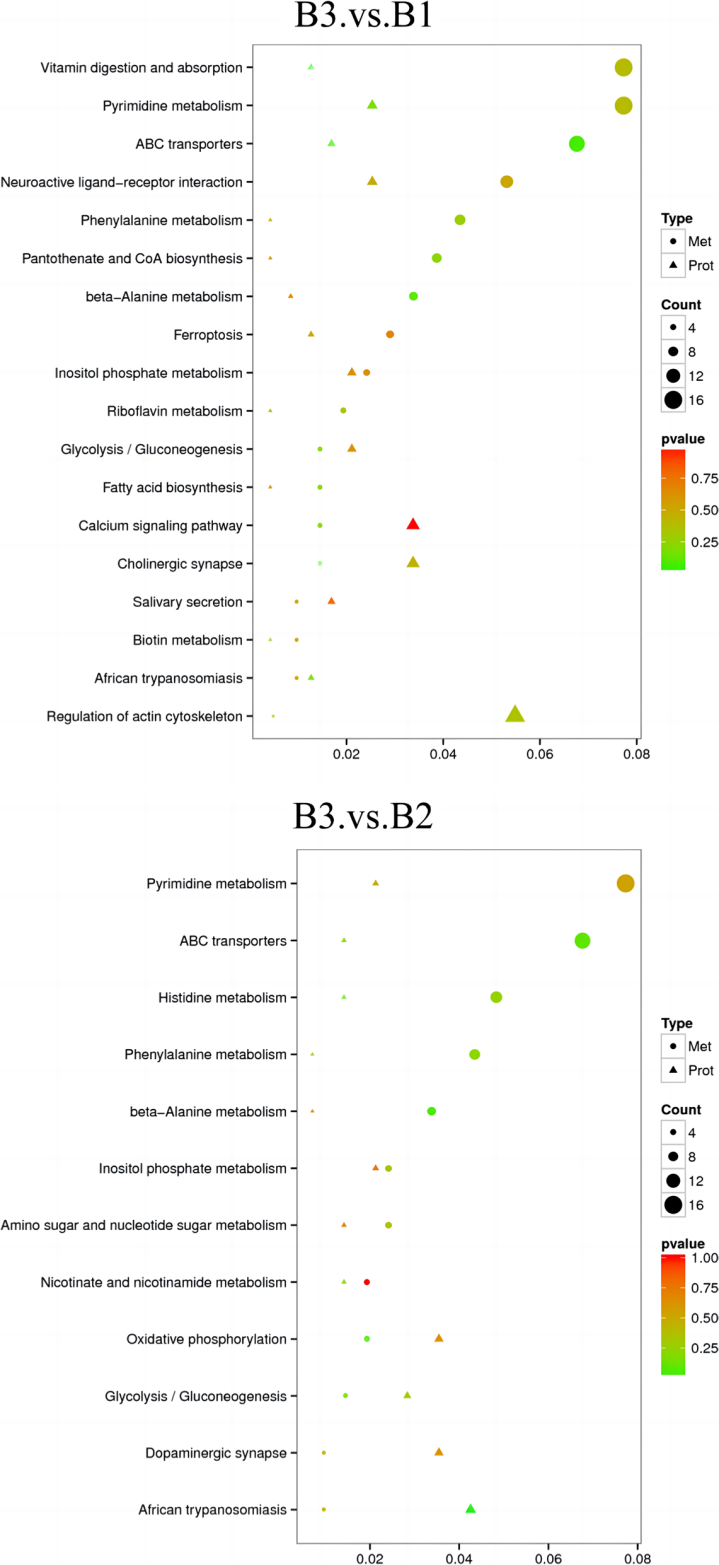
**A** KEGG analysis among the DEPs and DEMs between CUS cardiac surgery group (B3)and Control group (B1). **B** KEGG analysis among the DEPs and DEMs between CUS cardiac surgery group (B3)and cardiac surgery group (B2)

The heatmaps were applied to demonstrate the distribution of DEPs and DEMs between groups, and an obvious relevance was observed (Fig. 9A, B). The detailed list of DEMs and DEPs correlations between B3 and B1, B3 and B2 were shown in Supplement Tables 1 and 2. Furthermore, we analyzed all DEPs simultaneously with DEMs to obtain their corporate pathway enrichment information (Fig. 10 A, B). Between B3 and B1 group, B3 and B2 group, DEPs and DEMs were enriched in pyrimidine metabolism and beta-alanine metabolism pathway.

What’s more, between CUS cardiac surgery group and the control group, DEPs and DEMs tended to be enriched in ABC transporter pathways and histidine metabolism between the CUS cardiac surgery and cardiac surgery group, while DEPs and DEMs are enriched in several other metabolic pathways between CUS cardiac surgery group and cardiac surgery group.

## Discussion

As a common neurological complication after cardiac surgery, POD has been widely studied in previous studies [31].There are several theories regarding the pathophysiology of postoperative delirium based on findings from animal models, such as neuroinflammation, neurotransmitters, and subclinical cerebral vascular events. In addition, brain injury, especially the injury in hippocampus region is commonly found in POD patients and animal models [32, 33]. In this study, we used a CUS rat model to investigate the potential molecular biological mechanisms of brain damage following cardiac surgery. Rat hippocampus was extracted for integrative proteomics and metabolomics study. The results demonstrated significant differences among the comparison groups, suggesting that glycoproteins and metabolism of amino acids were the main possible mechanisms of brain injury in CUS rats following cardiac surgery. Specifically, chronic stress status has significant effects on the neurotransmitter pathway. What’s more, the underlying specific mechanisms and pathways need to be further investigated.

Previous studies showed that cardiac surgery with extracorporeal circulation is associated with major changes in metabolism and activates inflammatory cascades in patients [34], and suppression of neuroinflammation attenuates persistent cognitive and neurogenic deficits in a rat model of CPB [35]. However, few studies focused on metabolic changes in animals undergoing cardiac surgery.

To further analyze the changes in integrated proteomics and metabolomics, we further conducted the Integrated analysis of metabolomics and proteomics between each group. Combing the results of proteomics and metabolomics, the pathways of animo acids metabolism play an important role in the main mechanism of cardiac surgery related brain injury in preoperative CUS rats.Based on previous research,we focused on the pathway of lysine degardation and β-Alanine metabolism. L-Lysine is an essential proteogenic amino acid in humans. Previous research has demonstrated the roles of  lysine acetylation and deacetylation in brain development and neuropathies [36]. Lysine degradation is ketogenic yielding two acetyl-CoAs and several reduction equivalents and may be initiated either by ε-deamination or α-deamination. The ε-deamination is also known as the saccharopine pathway and is localized to the mitochondria. It is the major route for lysine degradation and is well-characterized at the molecular and biochemical levels. The lysine degradation pathway is relevant to the neurometabolic process, and the disturbance of this pathway can lead to the occurrence of two severe neurometabolic disorders, pyridoxine-dependent epilepsy (PDE) and glutaric aciduria type 1 (GA1) [37]. Our results suggested that the CUS state together with the trauma of cardiac surgery may disturb the lysine metabolism pathway and cause neurometabolic disorders. Meanwhile, beta-alanine metabolism is another pathway enriched in each group. β-alanine in the brain can function as either a neurotransmitter or a neuromodulator. The exact mechanisms underlying brain damage in beta-alanine disorder remain poorly understood. Some studies showed that high concentrations of β-alanine may cause metabolic disorders and disturbances in tissue redox state [38]. Patients affected by β-alaninemia present neurological dysfunction with convulsions, coma, somnolence, lethargy, hypotonia, hyporeflexia, developmental delay, and mental retardation. In addition, in the animal model, a previous study indicated that chronic exposure to β-Alanine generates oxidative stress and alters energy metabolism in cerebral cortex and cerebellum [39]. Contrary to the toxic effect of β-Alanine, there are studies suggesting that β-Alanine supplementation can reduce anxiety in both young and older rats by increasing the expression of brain derived neurotrophic factor (BDNF) in the subregion of the hippocampus [40]. Furthermore, Jay R. Hoffman. et al. proved that β-alanine can influence other metabolites in the hippocampus, and supplementation of this amino acid increases carnosine content in the hippocampus of middle-aged rats, without compromising histidine content [41]. But there are currently no reports on the effects of β-alanine on brain injury following cardiac surgery. Notably, regarding the cardiac system, Shetewy et al. suggested that the β-alanine was able to reduce contractile function in the hearts of rats, an effect related at least in part to impaired mitochondrial energy metabolism [42]. In our study, we found that beta-alanine metabolism in the hippocampus may be the potential mechanism of brain injury in CUS rats following cardiac surgery.

Our study has some limitations. To begin with, this study is an exploratory experiment, behavioral experiments were not included in this study. Subsequent experiments should be carried out to incorporate postoperative behavioral assessments to gauge alterations in the emotional and cognitive function of the CUS cardiac surgery rats. What’s more, in the DIA analysis, we did not perform the analysis of all proteins’ subcellular localization, which should be performed to accurate the percentage of DEPs’ subcellular localization in further study. What’s more, the current understanding of metabolites is relatively insufficient, therefore the conclusions obtained from analyzing metabolomic profiles are much less than data from analyzing proteomics, so the conclusions may not be complete. Furthermore, our investigation solely involved measurements at the protein and metabolite levels, without delving deeper into the underlying physiological mechanisms and causal relationships. For the further research, we will determine a research direction based on the results of proteomics and metabolomics, focusing on signaling pathways including some specific protein or metabolic substance. Finally, this study pertains to animal research, emphasizing the essential requirement for foundational investigations to elucidate the mechanisms and specific pathways involved in postoperative neurological injury following cardiac surgery in patients with chronic stress.

## Conclusion

In the present study, we applied DIA proteomics analysis, DEM analysis, and integrated analysis of metabolomics and proteomics to obtain a comprehensive demonstration of the rat hippocampal response to cardiac surgery in chronic stress states. Our results revealed the metabolism of amino acids, together with the signaling pathways of amino acids such as lysine degradation and beta-alanine metabolism were the main possible mechanisms of brain injury in CUS rats following cardiac surgery. These findings indicate a possible direction for the pathogenesis of brain injury after cardiac surgery in CUS rats and may help identify potential drug targets for perioperative brain protection.

## Fundings

Supported by the National Clinical Research Center of Cardiovascular Diseases, Fuwai Hospital, Chinese Academy of Medical Sciences (Grant No. NCRC2020014) and the National High-Level Hospital Clinical Research Funding (2022-GSP-GG-36).

### Supplementary Information

**Supplementary Material 1. **

## Data Availability

The data supporting the findings of the current study are available from the corresponding author on reasonable request.

## Abbreviations

BDNF: Brain Derived Neurotrophic Factor
CABG: Coronary Artery Bypass Grafting
COG: Cluster of Orthologous Groups of Proteins
CPB: Cardiopulmonary Bypass
CS: Chronic Stress
CUS: Chronic Unpredictable Stress
DEPs: Differentially Expressed Proteins
DEMs: Differentially Expressed Metabolites
DIA: Data-independent Acquisition
GO: Gene Ontology
HE: Hematoxylin–Eosin
HMDB: Human Metabolome Database
KEGG: Kyoto Encyclopedia of Genes and Genomes
PCA: Principal Components Analysis
PDE: Pyridoxine-Dependent Epilepsy
PLS-DA: Partial Least Squares Discriminant Analysis
POD: Postoperative Delirium
VIP: Variable Importance in the Projection
