# Supplementary material for: Integrative proteomics and metabolomics data analysis exploring the mechanism of brain injury after cardiac surgery in chronic stress rats
Source: BMC Anesthesiol. 2024 Mar 22;24:111. doi: 10.1186/s12871-024-02492-y (PMC10958840; doi:10.1186/s12871-024-02492-y)
Supplement: Supplementary file 1 — Supplementary Material 1. [file 12871_2024_2492_MOESM1_ESM.docx]

**
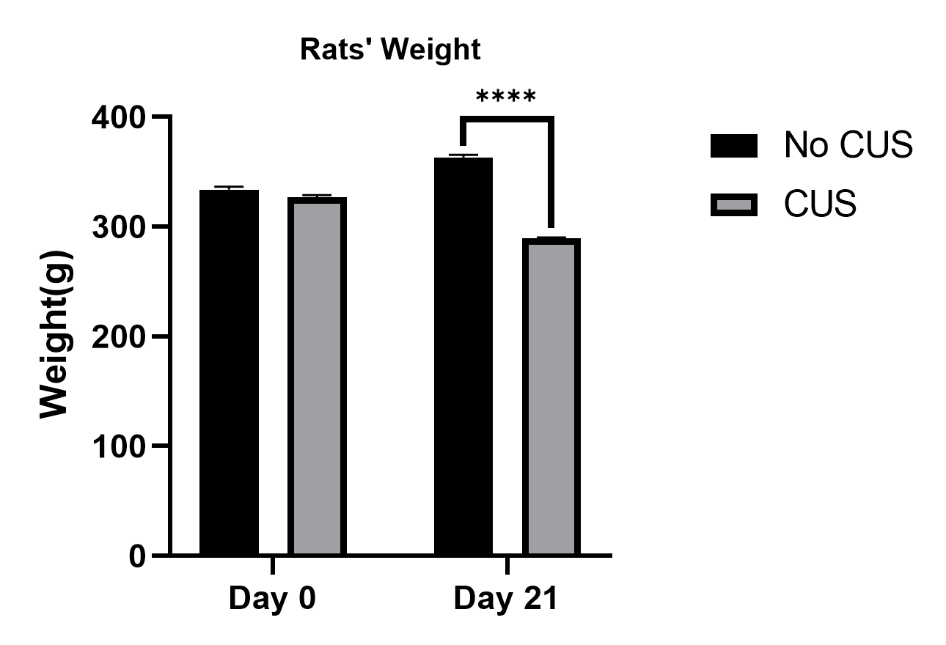
**

**Supplement Figure 1 Rats’ Weight in Day 0 and Day 21 （**** P<0.001）**


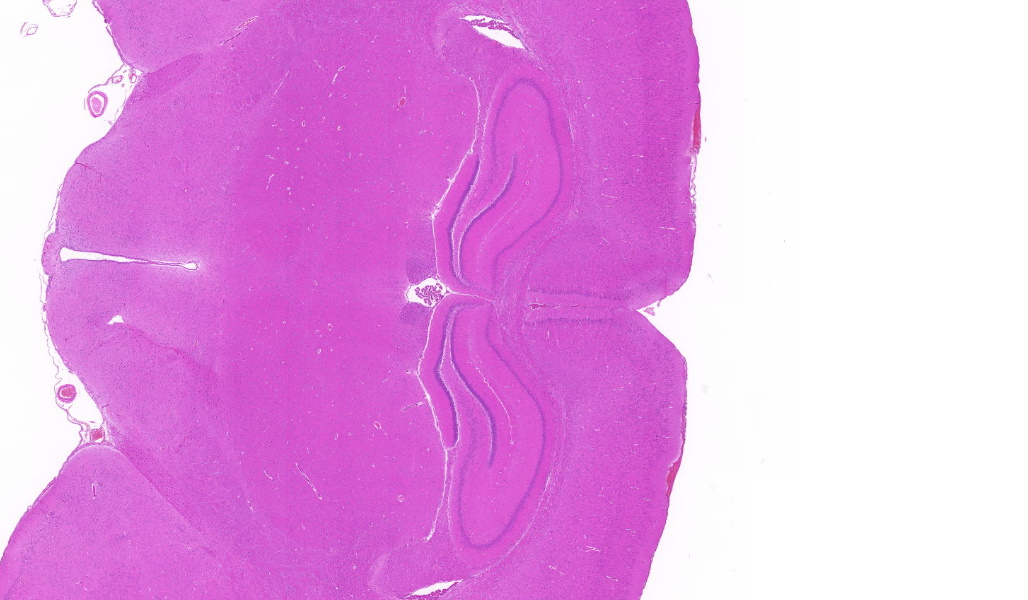


**Supplement Figure 2A** The hippocampus tissues of CUS and Cardiac Surgery Rats, at 2-fold magnification after HE staining,


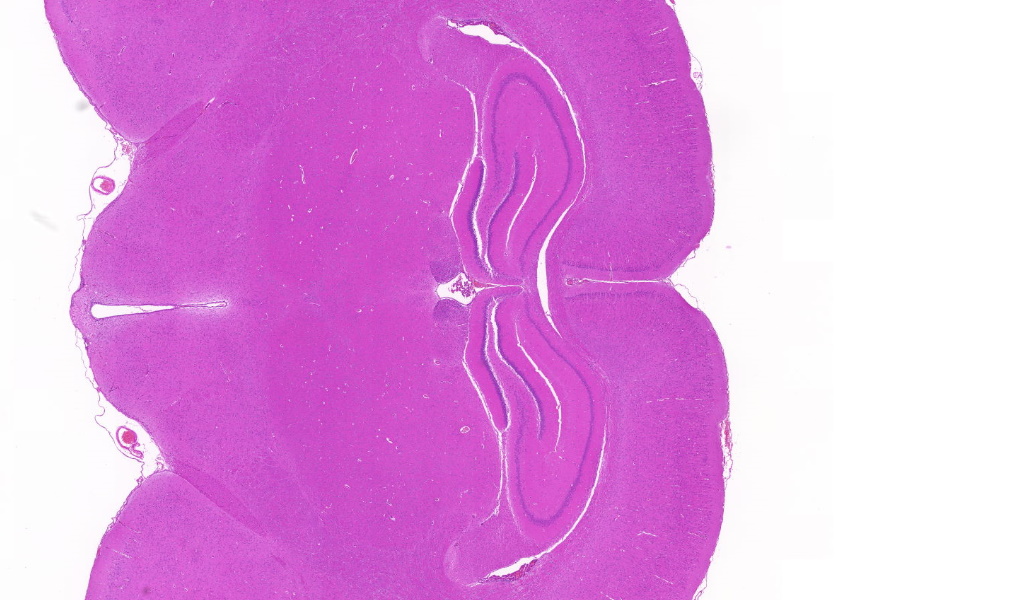


**Supplement Figure 2B** The hippocampus tissues of Cardiac Surgery Rats, at 2-fold magnification after HE staining,


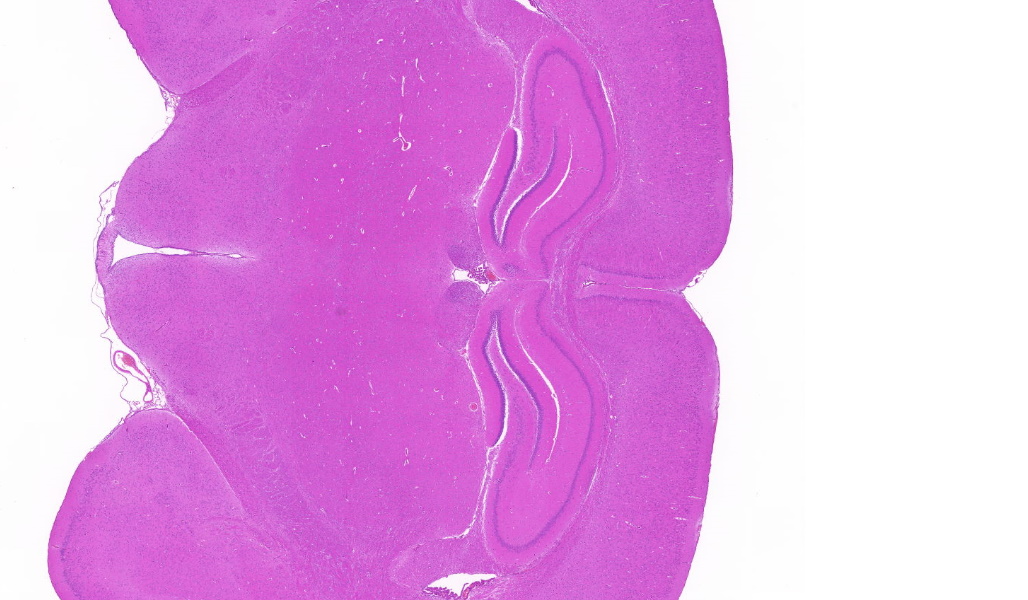


**Supplement Figure 2C** The hippocampus tissues of Normal Rats, at 2 fold magnification after HE staining,

**
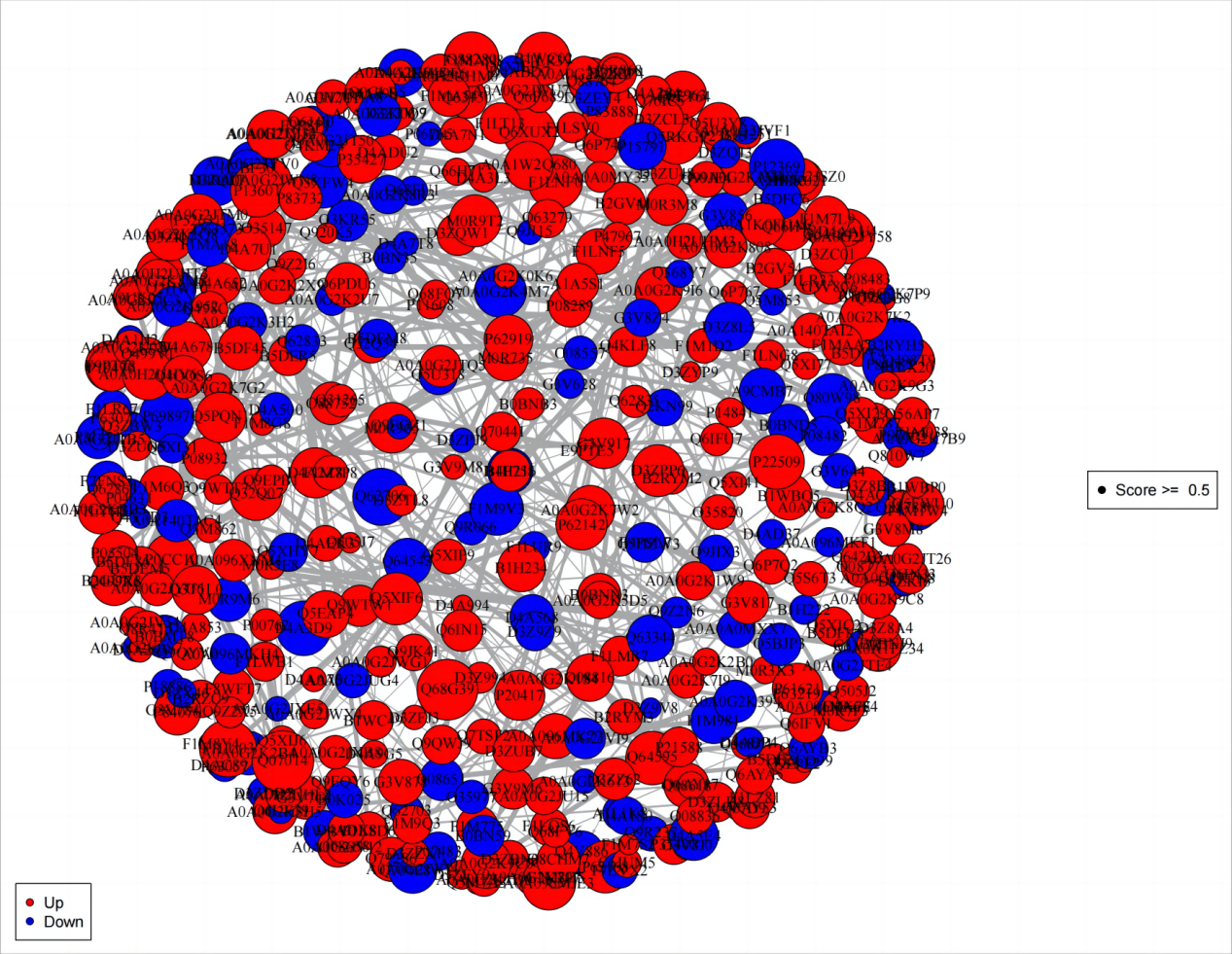
**

**Supplement Figure 3A Interaction Analysis of Identified Proteins between B3 and B1**

Each node in the interaction network represents a protein, and the node size represents the number of proteins interacting with it. The larger the node is, the more proteins interacting with it. The color of the nodes indicates the expression level of the protein in the comparison pair, with red representing significantly high expression of the protein and blue representing significantly low expression of the protein.


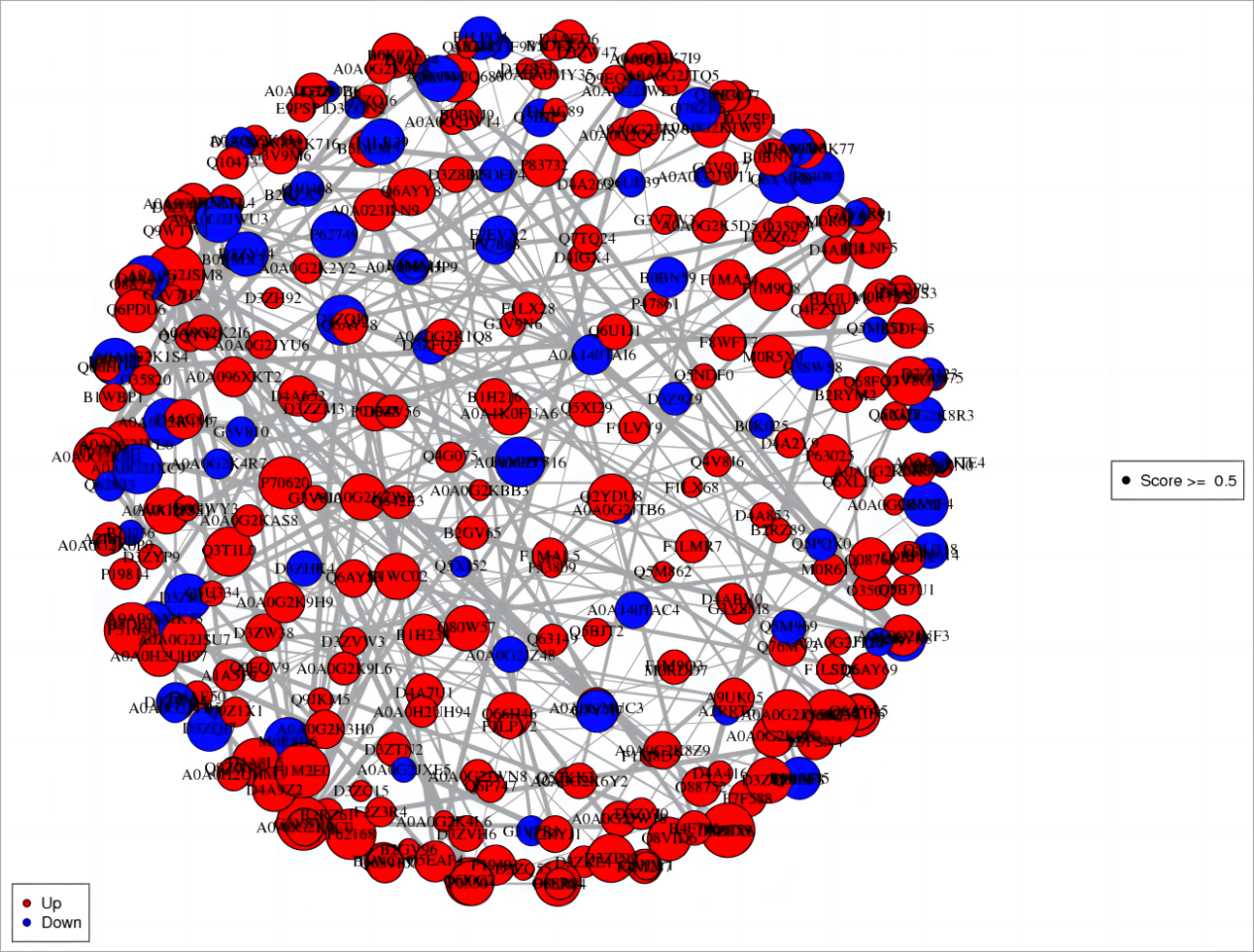


**Supplement Figure 3B Interaction Analysis of Identified Proteins between B3 and B2**

Each node in the interaction network represents a protein, and the node size represents the number of proteins interacting with it. The larger the node is, the more proteins interacting with it. The color of the nodes indicates the expression level of the protein in the comparison pair, with red representing significantly high expression of the protein and blue representing significantly low expression of the protein.


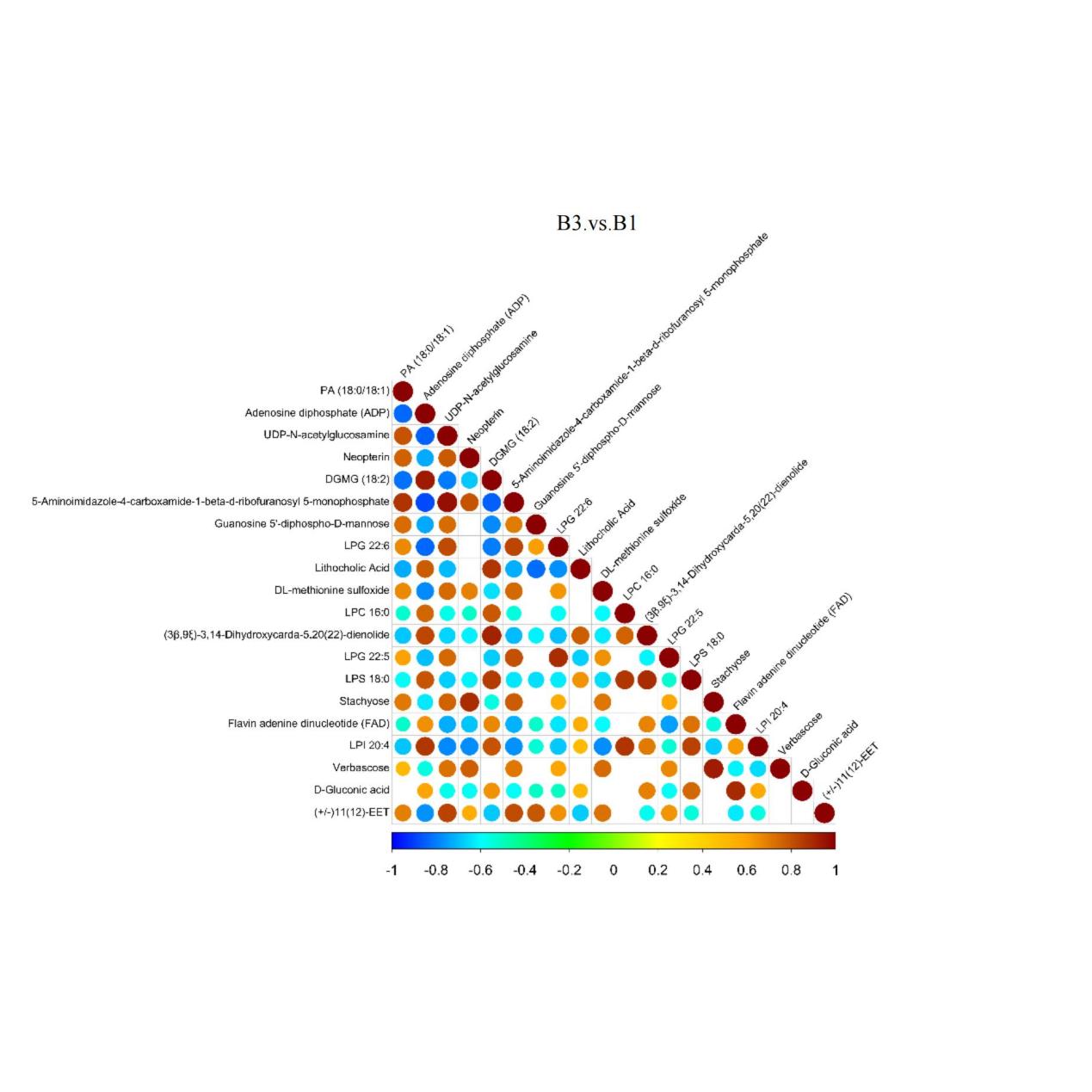


**Supplement Figure 4A** Differential Metabolite Correlation Analysis between B3 and B1(ESI- mode)


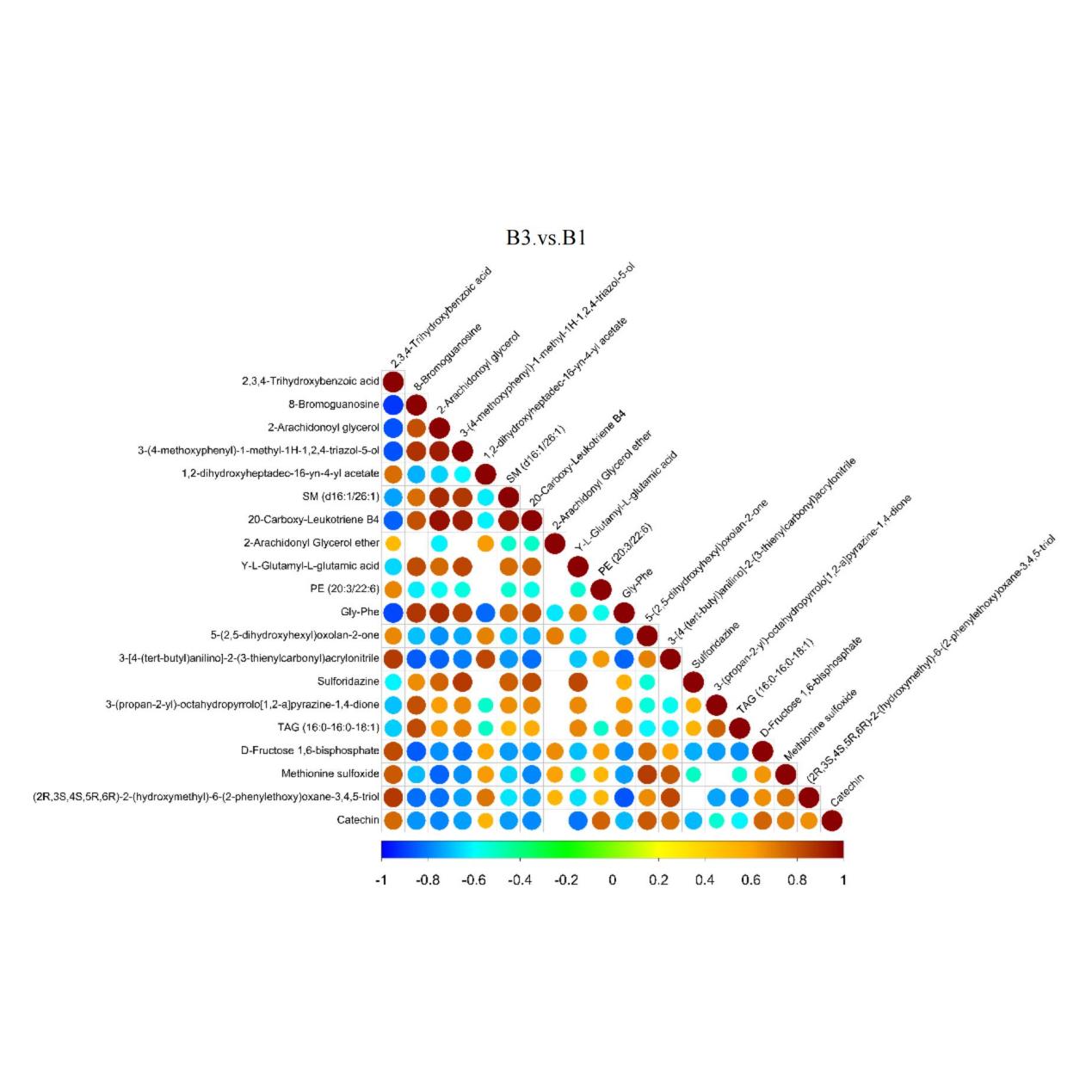


**Supplement Figure 4B** Differential Metabolite Correlation Analysis between B3 and B1(ESI+ mode)


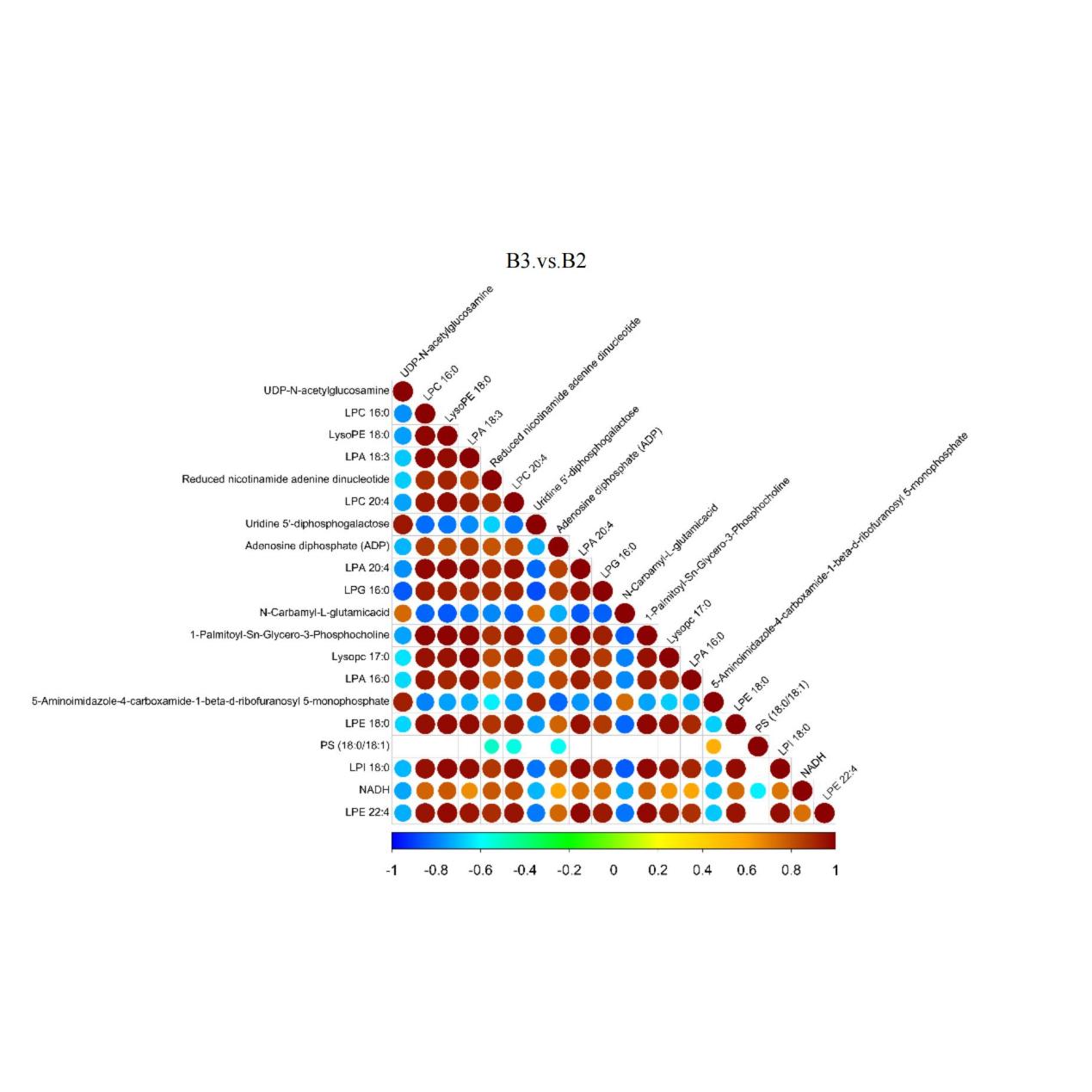


**Supplement Figure 4C** Differential Metabolite Correlation Analysis between B3 and B2(ESI- mode)


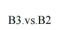

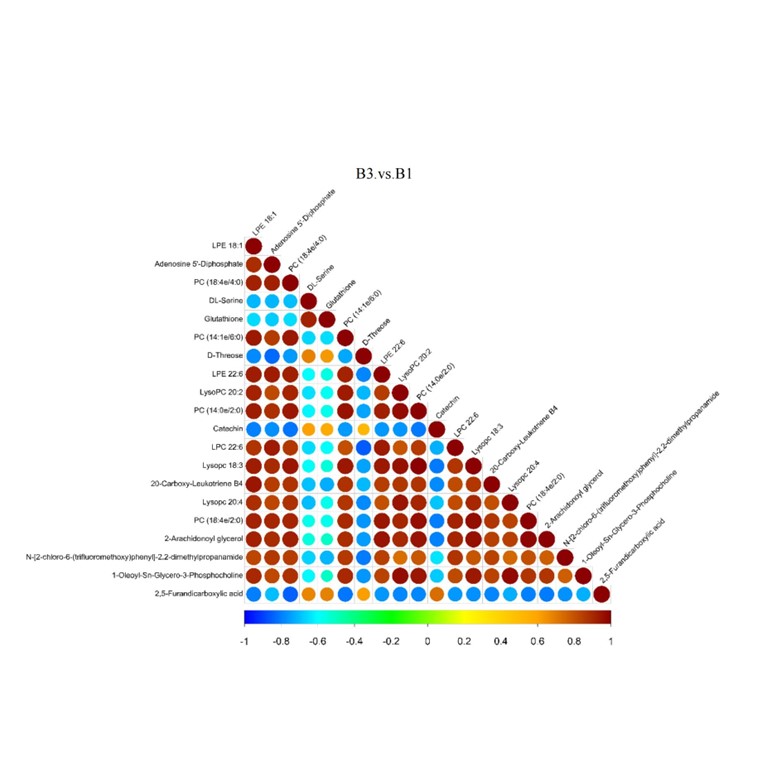


**Supplement Figure 4D** Differential Metabolite Correlation Analysis between B3 and B2(ESI+ mode)

**
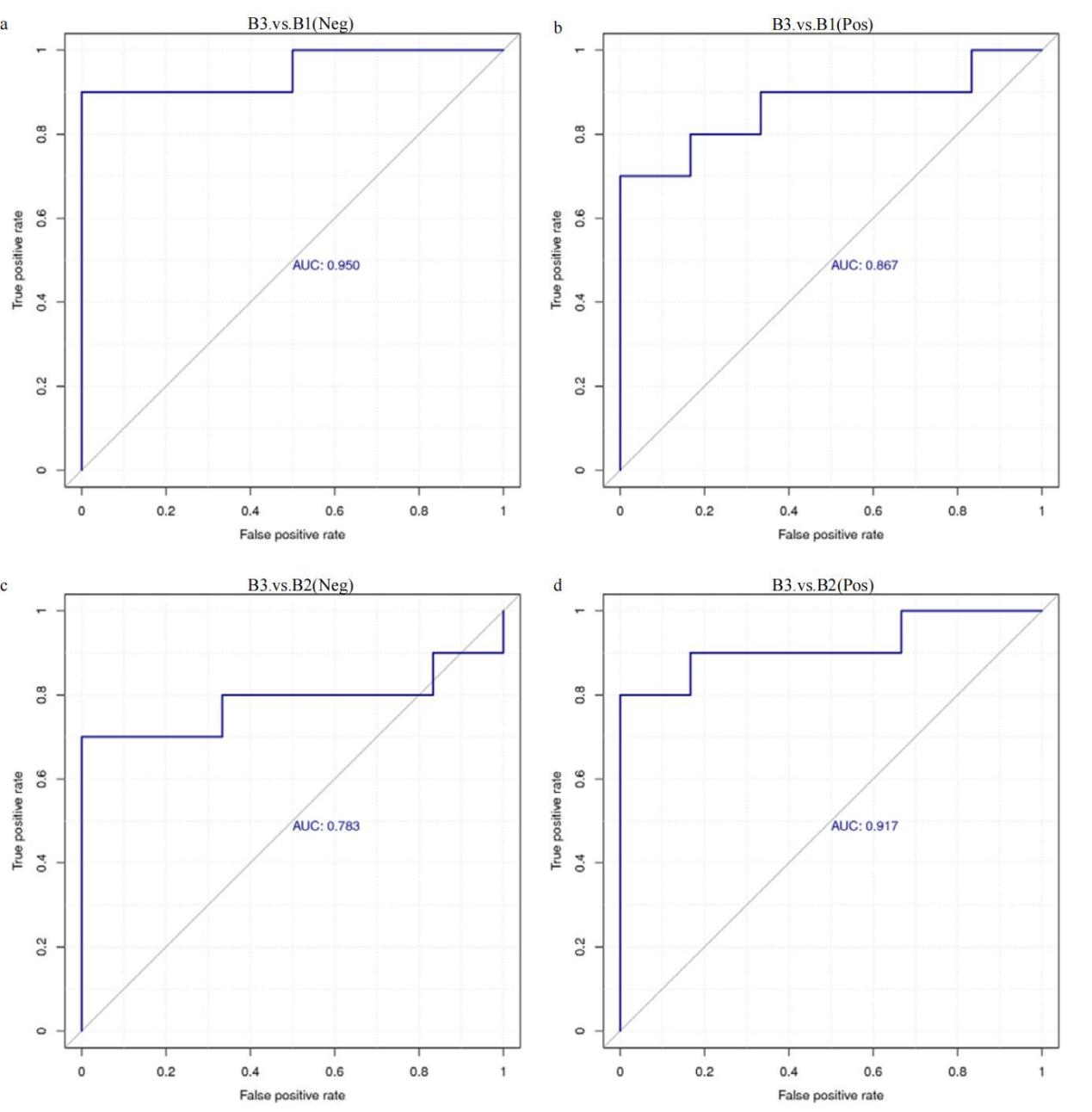
**

**Supplement Figure 5** ROC curves of DEMs between(a)B3 and B1(ESI-)(b)B3 and B1(ESI+) (c)B3 and B2(ESI-)(d) B3 and B2(ESI+)

**Supplement Table 1** The Detailed List of DEMs and DEPs Correlations between B3 and B1

| ID | Prot_A0A096MJE3 | Prot_A0A096MK73 | Prot_A0A096MK77 | Prot_A0A096MKF1 | Prot_A0A096MKH4 | Prot_A0A096XNM4 | Prot_A0A0A0MXX7 | Prot_A0A0A0MY35 | Prot_A0A0A1FZ34 |
| --- | --- | --- | --- | --- | --- | --- | --- | --- | --- |
| Com_4739_neg | 0.376858702 | -0.460327802 | 0.249242546 | -0.601008862 | -0.335144685 | 0.673443774 | -0.478348128 | 0.460366118 | 0.519093964 |
| Com_508_neg | -0.52908092 | 0.715538673 | -0.590420361 | 0.567622646 | 0.455782898 | -0.823526835 | 0.507087104 | -0.63332847 | -0.455931019 |
| Com_3647_pos | 0.591103717 | -0.544357969 | 0.473442277 | -0.575965889 | -0.495087762 | 0.81360466 | -0.467590698 | 0.647673695 | 0.364751462 |
| Com_4991_pos | -0.545242418 | 0.55329249 | -0.416831435 | 0.591383729 | 0.446902465 | -0.810530247 | 0.498060859 | -0.601611532 | -0.489896459 |
| Com_2609_pos | -0.436716382 | 0.671615531 | -0.573013431 | 0.664846786 | 0.46736895 | -0.824783024 | 0.53825176 | -0.808838503 | -0.522524191 |
| Com_586_pos | -0.390307236 | 0.536434893 | -0.450381482 | 0.616703705 | 0.434110271 | -0.806645927 | 0.508404958 | -0.744361957 | -0.472969391 |
| Com_165_neg | 0.311703354 | -0.705713412 | 0.694236949 | -0.442097797 | -0.358342453 | 0.730703875 | -0.405825026 | 0.669065599 | 0.649958834 |
| Com_6688_neg | 0.390581292 | -0.52529008 | 0.444866943 | -0.472767308 | -0.270234358 | 0.652124451 | -0.532746929 | 0.600822974 | 0.658917213 |
| Com_3685_neg | -0.493442795 | 0.631692837 | -0.435058956 | 0.605302694 | 0.402261383 | -0.803650698 | 0.613958205 | -0.584160687 | -0.471519264 |
| Com_313_pos | 0.428147951 | -0.455571341 | 0.344628959 | -0.279128736 | -0.260519461 | 0.639987293 | -0.603296136 | 0.49250094 | 0.482913192 |
| Com_15841_pos | -0.537133583 | 0.732981507 | -0.474762844 | 0.509440381 | 0.456860265 | -0.854703788 | 0.664948232 | -0.566107967 | -0.499126866 |
| Com_3856_pos | -0.535765602 | 0.645776056 | -0.501880203 | 0.634493172 | 0.470766305 | -0.843333478 | 0.548042536 | -0.667447446 | -0.535961794 |
| Com_1082_neg | 0.449735914 | -0.744458232 | 0.596008514 | -0.509126905 | -0.363403108 | 0.764678528 | -0.525316359 | 0.55297994 | 0.593493411 |
| Com_646_neg | 0.263124459 | -0.439297835 | 0.311228414 | -0.502641265 | -0.472218767 | 0.771465662 | -0.321107673 | 0.590833821 | 0.528242152 |
| Com_14903_pos | 0.189284131 | -0.6531901 | 0.803242234 | -0.170770829 | -0.180228336 | 0.416553036 | -0.239155128 | 0.547202993 | 0.355078316 |
| Com_2370_pos | -0.35663439 | 0.539558 | -0.174659062 | 0.648847985 | 0.481896255 | -0.7651481 | 0.603171018 | -0.58427426 | -0.460094354 |
| Com_4083_neg | -0.287717281 | 0.412998809 | -0.354268224 | 0.372647243 | 0.315015991 | -0.679793715 | 0.416023465 | -0.481594108 | -0.361319694 |
| Com_3695_pos | 0.672500501 | -0.452602013 | 0.279013695 | -0.458560426 | -0.429313975 | 0.506856375 | -0.304971157 | 0.320275854 | 0.143265353 |
| Com_12307_pos | -0.413750526 | 0.553205393 | -0.426886288 | 0.603537859 | 0.447861072 | -0.798062623 | 0.557977953 | -0.750317904 | -0.588185092 |
| Com_8577_pos | 0.265090114 | -0.640604484 | 0.646320708 | -0.208138565 | -0.482917243 | 0.709823263 | -0.437720119 | 0.788339027 | 0.433729982 |
| Com_24285_pos | 0.595728958 | -0.544219129 | 0.372051032 | -0.636218282 | -0.350103466 | 0.74441482 | -0.622912808 | 0.605117747 | 0.542747756 |
| Com_6860_pos | -0.287010768 | 0.454105251 | -0.341611346 | 0.576882753 | 0.345758568 | -0.618543312 | 0.343528565 | -0.558893805 | -0.254675331 |
| Com_2781_pos | -0.418175366 | 0.610209979 | -0.351517485 | 0.308383777 | 0.671289032 | -0.768211463 | 0.445986349 | -0.356456519 | -0.451474628 |
| Com_6127_pos | -0.286666047 | 0.465710603 | -0.401139514 | 0.396173724 | 0.481670319 | -0.568351053 | 0.253324538 | -0.557083535 | -0.266807758 |
| Com_842_neg | 0.437320959 | -0.572302877 | 0.696587045 | -0.406786445 | -0.313357813 | 0.546866081 | -0.195824508 | 0.619588639 | 0.208716844 |
| Com_5298_pos | 0.449073938 | -0.642022288 | 0.610381457 | -0.352986892 | -0.465582259 | 0.76802834 | -0.354838629 | 0.595979389 | 0.301437894 |
| Com_1241_neg | -0.371858227 | 0.51559186 | -0.430755511 | 0.503419415 | 0.211378499 | -0.6073729 | 0.481362876 | -0.513941135 | -0.355433134 |
| Com_124_pos | 0.42344119 | -0.581131403 | 0.726105888 | -0.398293601 | -0.429546823 | 0.727556823 | -0.312443816 | 0.81843217 | 0.520367033 |
| Com_20491_pos | 0.41442029 | -0.557813384 | 0.380262206 | -0.583441494 | -0.626802915 | 0.743828217 | -0.491250815 | 0.688676979 | 0.451598009 |
| Com_941_neg | -0.494105581 | 0.468638773 | -0.349553966 | 0.561441599 | 0.384740387 | -0.713027395 | 0.541856623 | -0.56586063 | -0.415921021 |
| Com_8285_pos | 0.575872829 | -0.660860548 | 0.460787844 | -0.511425577 | -0.472093429 | 0.763410122 | -0.494510952 | 0.641024515 | 0.2551895 |
| Com_2128_neg | 0.417109078 | -0.57388233 | 0.631160513 | -0.349791259 | -0.103760784 | 0.512727975 | -0.324933916 | 0.447110911 | 0.21722308 |
| Com_4694_pos | -0.54027772 | 0.647948951 | -0.585797695 | 0.240646138 | 0.617968381 | -0.769560424 | 0.290505691 | -0.570723256 | -0.219675626 |
| Com_1660_neg | -0.427846443 | 0.486234869 | -0.477006941 | 0.649138092 | 0.330941749 | -0.741249501 | 0.45522702 | -0.757960335 | -0.481665081 |
| Com_11428_pos | -0.598823936 | 0.534499097 | -0.496714122 | 0.364176048 | 0.439475983 | -0.691595099 | 0.356916614 | -0.518429712 | -0.598885435 |
| Com_3979_pos | 0.30931702 | -0.834849491 | 0.815083555 | -0.300576587 | -0.351935108 | 0.563151328 | -0.415070754 | 0.590337357 | 0.310547783 |
| Com_5474_pos | 0.332873961 | -0.803042787 | 0.759757637 | -0.23630374 | -0.383885178 | 0.528679598 | -0.432519277 | 0.515769761 | 0.30595222 |
| Com_12496_pos | 0.308361383 | -0.646620795 | 0.85999485 | -0.164451708 | -0.199175325 | 0.479388608 | -0.230566615 | 0.62539545 | 0.358053358 |
| Com_1089_neg | 0.252497356 | -0.508184723 | 0.514906266 | -0.202371264 | -0.142393333 | 0.447176411 | -0.428459074 | 0.43659489 | 0.439889116 |
| Com_20131_pos | -0.634574715 | 0.468440341 | -0.541182642 | 0.181741599 | 0.442098398 | -0.5924118 | 0.287750653 | -0.452223045 | -0.256986292 |
| Com_874_neg | -0.390697201 | 0.584722887 | -0.561849711 | 0.513072044 | 0.324088323 | -0.675922191 | 0.47894654 | -0.771078672 | -0.459894638 |
| Com_337_neg | -0.364231762 | 0.723079976 | -0.63262069 | 0.594015452 | 0.297459581 | -0.753808224 | 0.548714118 | -0.727855509 | -0.51019225 |
| Com_6829_pos | 0.443597625 | -0.676684285 | 0.4897976 | -0.23270023 | -0.715913142 | 0.777556292 | -0.46466833 | 0.623407722 | 0.180030278 |
| Com_1268_neg | 0.217902317 | -0.576094171 | 0.683482561 | -0.144009522 | -0.077068689 | 0.353387059 | -0.333235654 | 0.476502298 | 0.316582058 |
| Com_816_neg | -0.503670557 | 0.497048225 | -0.396045029 | 0.647153158 | 0.316776373 | -0.699314141 | 0.490319258 | -0.650402313 | -0.571518747 |
| Com_4404_neg | 0.429032617 | -0.613533407 | 0.628653748 | -0.318201711 | -0.607329769 | 0.757123142 | -0.184434642 | 0.591998083 | 0.422370294 |
| Com_777_neg | 0.423151452 | -0.759720808 | 0.79313813 | -0.277486716 | -0.313437558 | 0.542861469 | -0.318085372 | 0.564982537 | 0.378025193 |
| Com_2277_pos | 0.439066602 | -0.308422382 | 0.327058913 | -0.328414751 | -0.074257866 | 0.534618475 | -0.338842066 | 0.48672542 | 0.161167954 |
| Com_101_neg | 0.263949047 | -0.575985181 | 0.675926758 | -0.449990844 | -0.193396659 | 0.569367927 | -0.298742959 | 0.689644034 | 0.568542806 |
| Com_1024_neg | 0.449689629 | -0.613046375 | 0.578571954 | -0.580385704 | -0.366987222 | 0.738642196 | -0.461980508 | 0.678965933 | 0.509846655 |

Continued

| ID | Prot_A0A0G2JSZ0 | Prot_A0A0G2JT26 | Prot_A0A0G2JT50 | Prot_A0A0G2JTB5 | Prot_A0A0G2JTE4 | Prot_A0A0G2JTM0 | Prot_A0A0G2JTQ5 | Prot_A0A0G2JU15 | Prot_A0A0G2JUC3 |
| --- | --- | --- | --- | --- | --- | --- | --- | --- | --- |
| Com_4739_neg | 0.291667414 | 0.556302333 | -0.307203894 | -0.540406814 | -0.663352174 | -0.668663795 | 0.351584856 | 0.511865322 | 0.108002765 |
| Com_508_neg | -0.541926659 | -0.589859902 | 0.563046259 | 0.70238524 | 0.705452876 | 0.832867978 | -0.450662413 | -0.69019369 | 0.161561156 |
| Com_3647_pos | 0.399524639 | 0.518461112 | -0.396952843 | -0.522619054 | -0.675534127 | -0.683276866 | 0.513336818 | 0.621764191 | -0.054695558 |
| Com_4991_pos | -0.452696519 | -0.504267765 | 0.447336166 | 0.602747605 | 0.714540851 | 0.776032624 | -0.474617214 | -0.520765137 | -0.005492873 |
| Com_2609_pos | -0.588061652 | -0.674689031 | 0.550058131 | 0.640343396 | 0.727404967 | 0.701947403 | -0.418221458 | -0.765824337 | 0.16107963 |
| Com_586_pos | -0.522124701 | -0.555673031 | 0.519160113 | 0.631349359 | 0.704140352 | 0.745731348 | -0.355680854 | -0.593906972 | 0.025923042 |
| Com_165_neg | 0.674930181 | 0.513800207 | -0.539460532 | -0.624151754 | -0.722536192 | -0.631294224 | 0.448419088 | 0.743785886 | -0.260606352 |
| Com_6688_neg | 0.402871497 | 0.658325742 | -0.29083069 | -0.523436034 | -0.590249235 | -0.462318347 | 0.348495743 | 0.570407105 | -0.00302039 |
| Com_3685_neg | -0.584086022 | -0.529017622 | 0.510022022 | 0.695534098 | 0.769305427 | 0.653304285 | -0.381192954 | -0.56090522 | 0.065417011 |
| Com_313_pos | 0.339452498 | 0.611259104 | -0.241936955 | -0.376237767 | -0.580342082 | -0.39542898 | 0.380672054 | 0.473961544 | 0.069502407 |
| Com_15841_pos | 0.339452498 | -0.569252599 | 0.642618744 | 0.696037675 | 0.804902245 | 0.675775468 | -0.270147747 | -0.612010662 | 0.169275086 |
| Com_3856_pos | 0.339452498 | -0.554943163 | 0.521055257 | 0.687362253 | 0.782308547 | 0.683889541 | -0.319373806 | -0.643211831 | 0.152018475 |
| Com_1082_neg | 0.339452498 | 0.501068825 | -0.574567471 | -0.654166128 | -0.769425093 | -0.648884931 | 0.503082055 | 0.682245191 | -0.193788482 |
| Com_646_neg | 0.339452498 | 0.396849351 | -0.407386832 | -0.614573692 | -0.748615015 | -0.667183917 | 0.12764902 | 0.466208128 | 0.030400042 |
| Com_14903_pos | 0.339452498 | 0.508170526 | -0.406300396 | -0.294119589 | -0.360811593 | -0.46762112 | 0.649482184 | 0.810865468 | -0.407616037 |
| Com_2370_pos | 0.339452498 | -0.470192248 | 0.565439406 | 0.688639461 | 0.793000567 | 0.64922292 | -0.192928729 | -0.444745767 | -0.038157299 |
| Com_4083_neg | 0.339452498 | -0.360099914 | 0.362901563 | 0.518835644 | 0.629182859 | 0.619271042 | -0.373258646 | -0.380582261 | -0.063765105 |
| Com_3695_pos | 0.339452498 | 0.072392684 | -0.378710014 | -0.60137243 | -0.494561917 | -0.401804595 | 0.199105461 | 0.414293313 | -0.180741932 |
| Com_12307_pos | 0.339452498 | -0.678287382 | 0.431822184 | 0.481345104 | 0.729059683 | 0.643716134 | -0.464585786 | -0.67345822 | 0.024483527 |
| Com_8577_pos | 0.339452498 | 0.465386426 | -0.519130645 | -0.611054166 | -0.692777136 | -0.474568908 | 0.304661152 | 0.759617926 | -0.280539955 |
| Com_24285_pos | 0.339452498 | 0.583199251 | -0.371017851 | -0.645526357 | -0.664546404 | -0.537244154 | 0.336685958 | 0.475114206 | 0.018798007 |
| Com_6860_pos | 0.339452498 | -0.323051122 | 0.504949146 | 0.565950019 | 0.623973245 | 0.666328434 | -0.271866336 | -0.514955855 | 0.075963453 |
| Com_2781_pos | 0.339452498 | -0.482513586 | 0.414802967 | 0.500516863 | 0.639267527 | 0.769054131 | -0.351401941 | -0.444804382 | 0.248732038 |
| Com_6127_pos | 0.339452498 | -0.540300397 | 0.339380652 | 0.465653044 | 0.383133315 | 0.859424656 | -0.422867507 | -0.544044865 | 0.106402029 |
| Com_842_neg | 0.339452498 | 0.413794784 | -0.367963566 | -0.498681299 | -0.459539523 | -0.569624806 | 0.4932794 | 0.831187631 | -0.250171408 |
| Com_5298_pos | 0.339452498 | 0.410435645 | -0.567980647 | -0.534167388 | -0.680561143 | -0.812794008 | 0.532793463 | 0.71843657 | -0.180384137 |
| Com_1241_neg | 0.339452498 | -0.5209956 | 0.441640956 | 0.549320865 | 0.516749639 | 0.486715537 | -0.178579772 | -0.501356196 | 0.082535494 |
| Com_124_pos | 0.339452498 | 0.430415673 | -0.456781908 | -0.611910558 | -0.606750246 | -0.522154363 | 0.313348746 | 0.679505578 | -0.283620017 |
| Com_20491_pos | 0.339452498 | 0.747106112 | -0.316384427 | -0.441938933 | -0.582310302 | -0.691412308 | 0.463146342 | 0.654603146 | -0.157866902 |
| Com_941_neg | 0.339452498 | -0.363732867 | 0.437357226 | 0.631866893 | 0.670381227 | 0.527772505 | -0.273549273 | -0.434920263 | 0.057121546 |
| Com_8285_pos | 0.339452498 | 0.367029233 | -0.572725186 | -0.739228278 | -0.788367732 | -0.559791759 | 0.292288902 | 0.684501844 | -0.120955018 |
| Com_2128_neg | 0.339452498 | 0.234301992 | -0.505680207 | -0.340268816 | -0.574265764 | -0.551859452 | 0.756383189 | 0.613904601 | -0.197699838 |
| Com_4694_pos | 0.339452498 | -0.353296955 | 0.568670305 | 0.549065624 | 0.654016807 | 0.845759989 | -0.340865256 | -0.665875586 | 0.333599268 |
| Com_1660_neg | 0.339452498 | -0.508975274 | 0.450806327 | 0.579581699 | 0.67332059 | 0.56183359 | -0.27691688 | -0.587818811 | 0.056761067 |
| Com_11428_pos | 0.339452498 | -0.410930544 | 0.276058383 | 0.449486346 | 0.734717874 | 0.565083613 | -0.382365645 | -0.636263476 | 0.229994628 |
| Com_3979_pos | 0.339452498 | 0.535269458 | -0.603721078 | -0.380264671 | -0.522732813 | -0.530047698 | 0.762401549 | 0.857055671 | -0.535616929 |
| Com_5474_pos | 0.339452498 | 0.512252489 | -0.554480914 | -0.298076341 | -0.502421264 | -0.517524695 | 0.772691421 | 0.793256332 | -0.595380433 |
| Com_12496_pos | 0.339452498 | 0.42773136 | -0.439201199 | -0.383860362 | -0.393287868 | -0.417374921 | 0.554092463 | 0.793109824 | -0.400417769 |
| Com_1089_neg | 0.339452498 | 0.570870789 | -0.235917849 | -0.298099038 | -0.432816696 | -0.37468549 | 0.513933897 | 0.618333635 | -0.128505474 |
| Com_20131_pos | 0.339452498 | -0.204628391 | 0.335146124 | 0.439924534 | 0.553214373 | 0.611700877 | -0.340553272 | -0.531067215 | 0.364750108 |
| Com_874_neg | 0.339452498 | -0.443181745 | 0.514364453 | 0.463756 | 0.710427631 | 0.43788259 | -0.542273056 | -0.677791618 | 0.175580418 |
| Com_337_neg | 0.339452498 | -0.757715256 | 0.58463163 | 0.607360587 | 0.619174359 | 0.662730622 | -0.415555688 | -0.763162092 | 0.123596505 |
| Com_6829_pos | 0.339452498 | 0.40426942 | -0.601657393 | -0.605522607 | -0.694158161 | -0.676676469 | 0.250521628 | 0.64879187 | -0.378302752 |
| Com_1268_neg | 0.339452498 | 0.493805034 | -0.326405071 | -0.218986898 | -0.355012026 | -0.318407891 | 0.644888695 | 0.724555245 | -0.304479192 |
| Com_816_neg | 0.339452498 | -0.379798389 | 0.489784593 | 0.479432802 | 0.693293869 | 0.458128697 | -0.387269727 | -0.420568382 | 0.094049685 |
| Com_4404_neg | 0.339452498 | 0.275828785 | -0.459206775 | -0.597078258 | -0.695141189 | -0.671512187 | 0.347626539 | 0.701219465 | -0.32377793 |
| Com_777_neg | 0.339452498 | 0.402349284 | -0.54938648 | -0.606969908 | -0.490297732 | -0.52550208 | 0.451904196 | 0.77407074 | -0.447445039 |
| Com_2277_pos | 0.339452498 | 0.184304275 | -0.37046542 | -0.466428764 | -0.580274565 | -0.460376918 | 0.304862472 | 0.440876763 | 0.214695568 |
| Com_101_neg | 0.339452498 | 0.484940089 | -0.397415802 | -0.393004578 | -0.643053495 | -0.482917869 | 0.555941147 | 0.788748536 | -0.234137627 |
| Com_1024_neg | 0.339452498 | 0.62302706 | -0.375601513 | -0.562750081 | -0.702838613 | -0.63709091 | 0.475241979 | 0.744138562 | -0.150467556 |

Continued

| ID | Prot_A0A0G2JUG4 | Prot_A0A0G2JV12 | Prot_A0A0G2JV35 | Prot_A0A0G2JV54 | Prot_A0A0G2JVC2 | Prot_A0A0G2JVF1 | Prot_A0A0G2JVI9 | Prot_A0A0G2JWG1 | Prot_A0A0G2JWJ4 |
| --- | --- | --- | --- | --- | --- | --- | --- | --- | --- |
| Com_4739_neg | -0.58326741 | 0.34383791 | 0.383644783 | 0.309353819 | 0.523998611 | -0.589439813 | -0.428443753 | 0.391237207 | -0.607740111 |
| Com_508_neg | 0.82288081 | -0.523538112 | -0.58379301 | -0.404140215 | -0.627265053 | 0.55582421 | 0.585266244 | -0.579287458 | 0.506668113 |
| Com_3647_pos | -0.53620648 | 0.546338112 | 0.513093343 | 0.474739758 | 0.606025362 | -0.267808804 | -0.305624504 | 0.369036768 | -0.335055724 |
| Com_4991_pos | 0.597323271 | -0.471827757 | -0.451436158 | -0.49159412 | -0.551150643 | 0.475246924 | 0.452165972 | -0.434107717 | 0.491773963 |
| Com_2609_pos | 0.678641529 | -0.640786959 | -0.515591831 | -0.431462895 | -0.557721829 | 0.378581789 | 0.543158171 | -0.579491681 | 0.344954946 |
| Com_586_pos | 0.581405019 | -0.489987429 | -0.50849157 | -0.477628602 | -0.453157472 | 0.443056355 | 0.475828758 | -0.60759365 | 0.524275835 |
| Com_165_neg | -0.718756422 | 0.646933372 | 0.383424201 | 0.36243681 | 0.687599255 | -0.490339688 | -0.637609517 | 0.533828901 | -0.540587125 |
| Com_6688_neg | -0.490686475 | 0.748263297 | 0.320007713 | 0.142858639 | 0.595329276 | -0.351402998 | -0.299417487 | 0.371458497 | -0.269494184 |
| Com_3685_neg | 0.731091699 | -0.447761452 | -0.483592523 | -0.42395105 | -0.647818892 | 0.635094744 | 0.505635333 | -0.652639984 | 0.604501456 |
| Com_313_pos | -0.391099135 | 0.721024806 | 0.445958258 | 0.045116909 | 0.562743249 | -0.297168993 | -0.092313296 | 0.274888296 | -0.271802583 |
| Com_15841_pos | 0.78251136 | -0.473483431 | -0.58774 | -0.270891528 | -0.593356723 | 0.594713637 | 0.526032965 | -0.722781734 | 0.51950717 |
| Com_3856_pos | 0.713268334 | -0.50951288 | -0.502398063 | -0.42810306 | -0.650254109 | 0.533968204 | 0.517753828 | -0.685199424 | 0.499313799 |
| Com_1082_neg | -0.767825262 | 0.56759077 | 0.429895506 | 0.337954507 | 0.690849693 | -0.554747401 | -0.593853562 | 0.494635372 | -0.547815014 |
| Com_646_neg | -0.549206534 | 0.138327498 | 0.230502367 | 0.576201357 | 0.510973075 | -0.634281822 | -0.604920609 | 0.64694396 | -0.776068098 |
| Com_14903_pos | -0.597001258 | 0.838768314 | 0.486092325 | 0.087042568 | 0.540439955 | -0.080042294 | -0.422982693 | 0.11488327 | -0.072922799 |
| Com_2370_pos | 0.624514508 | -0.204833448 | -0.257188377 | -0.397021284 | -0.306587398 | 0.704132385 | 0.61923558 | -0.564524151 | 0.587868945 |
| Com_4083_neg | 0.514621408 | -0.286034627 | -0.408611961 | -0.506083068 | -0.557066108 | 0.553038758 | 0.396257768 | -0.531870241 | 0.760502359 |
| Com_3695_pos | -0.516870408 | 0.30114718 | 0.158133758 | -0.029552908 | 0.266368647 | -0.380401569 | -0.186961442 | 0.120269072 | -0.11251959 |
| Com_12307_pos | 0.476069875 | -0.675409312 | -0.45057569 | -0.377992388 | -0.503169511 | 0.269985767 | 0.396039132 | -0.372619276 | 0.305016841 |
| Com_8577_pos | -0.614857124 | 0.751411976 | 0.297772309 | 0.08291888 | 0.507341533 | -0.451946793 | -0.461742767 | 0.401053794 | -0.40726405 |
| Com_24285_pos | -0.564069513 | 0.612899351 | 0.368474535 | 0.299213828 | 0.570105817 | -0.457886139 | -0.317856615 | 0.499356021 | -0.273224988 |
| Com_6860_pos | 0.610924471 | -0.165903158 | -0.448415647 | -0.454388232 | -0.302088019 | 0.554611827 | 0.538018755 | -0.56754393 | 0.597085427 |
| Com_2781_pos | 0.673930099 | -0.305437237 | -0.313399304 | -0.468169227 | -0.700366233 | 0.505249775 | 0.64716921 | -0.416172397 | 0.530167762 |
| Com_6127_pos | 0.59238805 | -0.417934035 | -0.367899035 | -0.326615275 | -0.29277227 | 0.369050663 | 0.553001226 | -0.166082308 | 0.278525352 |
| Com_842_neg | -0.643976243 | 0.635455946 | 0.503748042 | 0.117439925 | 0.494398413 | -0.235039192 | -0.300591701 | 0.192495348 | -0.196991467 |
| Com_5298_pos | -0.686040466 | 0.478386797 | 0.609955118 | 0.339503073 | 0.502456195 | -0.404549587 | -0.472247711 | 0.346545145 | -0.536141407 |
| Com_1241_neg | 0.593259136 | -0.403498049 | -0.562392222 | -0.265453993 | -0.46562894 | 0.379898441 | 0.335579548 | -0.709651537 | 0.347436949 |
| Com_124_pos | -0.527179646 | 0.754206686 | 0.322299372 | 0.357283322 | 0.582235156 | -0.237830471 | -0.409983516 | 0.521946703 | -0.23380259 |
| Com_20491_pos | -0.543265342 | 0.613642123 | 0.334575676 | 0.450511578 | 0.563665559 | -0.234487798 | -0.513866898 | 0.262938865 | -0.119027528 |
| Com_941_neg | 0.515197525 | -0.461516678 | -0.392295738 | -0.321953293 | -0.491334458 | 0.46744577 | 0.298452508 | -0.570588214 | 0.478319991 |
| Com_8285_pos | -0.754805692 | 0.404486067 | 0.376862725 | 0.166825068 | 0.425513664 | -0.645627962 | -0.445660251 | 0.427889761 | -0.427275572 |
| Com_2128_neg | -0.583600025 | 0.528964841 | 0.641386108 | 0.336881421 | 0.46637141 | -0.29507878 | -0.339520859 | 0.217391572 | -0.378536205 |
| Com_4694_pos | 0.742548747 | -0.407637168 | -0.572418262 | -0.275962274 | -0.450575237 | 0.464725413 | 0.540175912 | -0.393823085 | 0.40713587 |
| Com_1660_neg | 0.500193052 | -0.525238305 | -0.488527103 | -0.462234944 | -0.475502699 | 0.333896712 | 0.364555901 | -0.66659091 | 0.367952827 |
| Com_11428_pos | 0.563282335 | -0.676938913 | -0.381166535 | -0.139281313 | -0.693799738 | 0.456567627 | 0.341347093 | -0.267947564 | 0.351448189 |
| Com_3979_pos | -0.750289604 | 0.792781673 | 0.514399321 | 0.286445572 | 0.615720442 | -0.145593796 | -0.615768128 | 0.21006324 | -0.051217474 |
| Com_5474_pos | -0.714171366 | 0.818071039 | 0.51928643 | 0.247610386 | 0.630118557 | -0.127411406 | -0.589415602 | 0.155040733 | -0.001533863 |
| Com_12496_pos | -0.553656121 | 0.891083786 | 0.466816129 | 0.056694957 | 0.527223536 | -0.041375877 | -0.313515336 | 0.190296702 | -0.022239432 |
| Com_1089_neg | -0.483929993 | 0.787077046 | 0.442215069 | -0.043762754 | 0.570599155 | -0.245923473 | -0.213802294 | 0.137614827 | -0.221231378 |
| Com_20131_pos | 0.568202521 | -0.635934568 | -0.564855146 | -0.027642864 | -0.521838412 | 0.396611614 | 0.204727484 | -0.299934362 | 0.301684082 |
| Com_874_neg | 0.466710077 | -0.717803448 | -0.366002142 | -0.366964844 | -0.440873875 | 0.232317718 | 0.394772993 | -0.322752701 | 0.188766038 |
| Com_337_neg | 0.715102451 | -0.637517052 | -0.614876566 | -0.353089549 | -0.536269881 | 0.31515684 | 0.525258116 | -0.624484016 | 0.284752023 |
| Com_6829_pos | -0.744291045 | 0.434852878 | 0.421779448 | 0.21374787 | 0.445228101 | -0.526641801 | -0.574776356 | 0.449320556 | -0.380307957 |
| Com_1268_neg | -0.499371735 | 0.871083845 | 0.497225741 | -0.050826841 | 0.497301121 | -0.074144128 | -0.240747859 | 0.04371623 | -0.01936867 |
| Com_816_neg | 0.369451684 | -0.52634269 | -0.295377019 | -0.547094028 | -0.411849663 | 0.209407024 | 0.392125046 | -0.483286096 | 0.150248274 |
| Com_4404_neg | -0.655843367 | 0.42112312 | 0.251786366 | 0.396342519 | 0.657978462 | -0.435182927 | -0.569443743 | 0.361668374 | -0.533485292 |
| Com_777_neg | -0.778527545 | 0.756232451 | 0.387221928 | 0.005016569 | 0.524997638 | -0.352074765 | -0.506487524 | 0.285846635 | -0.135660159 |
| Com_2277_pos | -0.379476218 | 0.355213904 | 0.540197934 | 0.013191645 | 0.157136506 | -0.447164615 | 0.002352918 | 0.263692837 | -0.512038563 |
| Com_101_neg | -0.55145619 | 0.726976945 | 0.429819738 | 0.311485496 | 0.583751105 | -0.297341474 | -0.473042303 | 0.329536352 | -0.330749491 |
| Com_1024_neg | -0.700309562 | 0.634675185 | 0.527249912 | 0.394425367 | 0.730836363 | -0.478564158 | -0.476817674 | 0.520164075 | -0.452273545 |

Continued

| ID | Prot_A0A0G2JWN8 | Prot_A0A0G2JWU7 | Prot_A0A0G2JWX4 | Prot_A0A0G2JX90 | Prot_A0A0G2JXB8 | Prot_A0A0G2JXE5 | Prot_A0A0G2JY12 | Prot_A0A0G2JY58 | Prot_A0A0G2JYU6 |
| --- | --- | --- | --- | --- | --- | --- | --- | --- | --- |
| Com_4739_neg | 0.311500993 | 0.643272944 | 0.580696617 | 0.213422043 | 0.476435326 | -0.459993635 | 0.243906806 | 0.533431127 | 0.692795726 |
| Com_508_neg | -0.653260334 | -0.722313424 | -0.355740174 | -0.170395273 | -0.669809396 | 0.599557644 | -0.555412246 | -0.402716211 | -0.781761162 |
| Com_3647_pos | 0.408304225 | 0.47357227 | 0.216305963 | 0.253015145 | 0.779293439 | -0.589885844 | 0.462615109 | 0.3147376 | 0.730506256 |
| Com_4991_pos | -0.339053728 | -0.571828401 | -0.31004046 | -0.336848605 | -0.659879789 | 0.587173192 | -0.395856503 | -0.422662626 | -0.778807961 |
| Com_2609_pos | -0.645687442 | -0.617909791 | -0.305865603 | -0.343156013 | -0.629671447 | 0.663418403 | -0.531071709 | -0.330394229 | -0.836074659 |
| Com_586_pos | -0.519252938 | -0.688618485 | -0.391744586 | -0.386386674 | -0.600039516 | 0.640217399 | -0.419058096 | -0.451858036 | -0.843761189 |
| Com_165_neg | 0.553991284 | 0.665700856 | 0.227819703 | 0.089762689 | 0.538276334 | -0.429530702 | 0.612700075 | 0.433220293 | 0.793740953 |
| Com_6688_neg | 0.331995617 | 0.411600608 | 0.338762864 | 0.298514714 | 0.667308977 | -0.652804377 | 0.37684049 | 0.374240281 | 0.787642684 |
| Com_3685_neg | -0.579736306 | -0.618692871 | -0.422698433 | -0.265508119 | -0.603214231 | 0.610292085 | -0.377164205 | -0.476424387 | -0.798987906 |
| Com_313_pos | 0.261690362 | 0.355404641 | 0.376965606 | 0.187938111 | 0.705522167 | -0.577539634 | 0.267389867 | 0.451994085 | 0.708253963 |
| Com_15841_pos | -0.686601192 | -0.6225957 | -0.430206947 | -0.185815568 | -0.56832105 | 0.542092967 | -0.402419191 | -0.411878199 | -0.756296955 |
| Com_3856_pos | -0.63758547 | -0.606921285 | -0.385770821 | -0.308993696 | -0.61290508 | 0.605481994 | -0.438000388 | -0.411123134 | -0.789591755 |
| Com_1082_neg | 0.514409976 | 0.59889657 | 0.249417902 | 0.075665344 | 0.562565976 | -0.444062966 | 0.555773765 | 0.370335984 | 0.7995002 |
| Com_646_neg | 0.276266082 | 0.658137624 | 0.501183924 | 0.29854423 | 0.387596235 | -0.310141793 | 0.177204957 | 0.696560546 | 0.504324368 |
| Com_14903_pos | 0.614943043 | 0.517242718 | -0.073769584 | -0.217840231 | 0.501515366 | -0.316482538 | 0.796248638 | 0.08832539 | 0.63773746 |
| Com_2370_pos | -0.326551341 | -0.533320348 | -0.52004016 | -0.506812283 | -0.276867873 | 0.573020636 | -0.184200389 | -0.502093497 | -0.704279094 |
| Com_4083_neg | -0.335554973 | -0.674859156 | -0.391276287 | -0.134000952 | -0.560505881 | 0.390560639 | -0.25407405 | -0.638444935 | -0.639984844 |
| Com_3695_pos | 0.211032586 | 0.033174718 | 0.13828361 | 0.324969782 | 0.380019249 | -0.634833348 | 0.52535499 | -0.035774965 | 0.594120449 |
| Com_12307_pos | -0.371919353 | -0.525442401 | -0.319710717 | -0.372073577 | -0.6236592 | 0.595768019 | -0.396791465 | -0.379109812 | -0.835295511 |
| Com_8577_pos | 0.462617223 | 0.500898963 | 0.306727967 | 0.225579969 | 0.536444247 | -0.658737294 | 0.639337139 | 0.547197224 | 0.805639294 |
| Com_24285_pos | 0.392220654 | 0.337916831 | 0.319696878 | 0.498738734 | 0.687353601 | -0.793888683 | 0.332176321 | 0.302161178 | 0.807226945 |
| Com_6860_pos | -0.538349462 | -0.661052066 | -0.406687972 | -0.274775493 | -0.243292766 | 0.394893341 | -0.366398409 | -0.388287968 | -0.614587573 |
| Com_2781_pos | -0.448887942 | -0.684479603 | -0.284900723 | 0.030537579 | -0.593415386 | 0.35680304 | -0.265821253 | -0.537848193 | -0.548250415 |
| Com_6127_pos | -0.376116318 | -0.694428534 | -0.309049186 | -0.216406795 | -0.48652286 | 0.582761116 | -0.464608707 | -0.381620376 | -0.609554775 |
| Com_842_neg | 0.548876926 | 0.464863358 | 0.170759531 | 0.014824771 | 0.585280039 | -0.513425602 | 0.793298843 | 0.149834412 | 0.641149943 |
| Com_5298_pos | 0.480603763 | 0.703976339 | 0.265372919 | -0.011052621 | 0.592428535 | -0.376769617 | 0.637171411 | 0.41656461 | 0.699726219 |
| Com_1241_neg | -0.707540603 | -0.527031347 | -0.340150916 | -0.179120188 | -0.507782172 | 0.485833144 | -0.327827167 | -0.236426623 | -0.585418783 |
| Com_124_pos | 0.499244725 | 0.401418923 | 0.047880144 | 0.354794237 | 0.690050694 | -0.664842162 | 0.654769445 | 0.282128712 | 0.763480646 |
| Com_20491_pos | 0.458420185 | 0.515220354 | 0.253256156 | 0.330375658 | 0.655888615 | -0.672381585 | 0.355441108 | 0.340306095 | 0.686837051 |
| Com_941_neg | -0.465243681 | -0.460241551 | -0.335750035 | -0.390419392 | -0.580735803 | 0.693954627 | -0.374288633 | -0.348061496 | -0.855081215 |
| Com_8285_pos | 0.411331904 | 0.340684982 | 0.398656134 | 0.357503225 | 0.431313638 | -0.634614313 | 0.534498489 | 0.375473028 | 0.656924931 |
| Com_2128_neg | 0.477970049 | 0.472525276 | -0.022677711 | -0.059090146 | 0.382921346 | -0.204363116 | 0.667533831 | 0.12097727 | 0.661388661 |
| Com_4694_pos | -0.560599655 | -0.63840962 | -0.27807239 | -0.076071595 | -0.495388986 | 0.414717685 | -0.604286207 | -0.423018026 | -0.551406726 |
| Com_1660_neg | -0.557645619 | -0.4966856 | -0.293417194 | -0.453800994 | -0.554248741 | 0.599490456 | -0.40185523 | -0.306098555 | -0.751183358 |
| Com_11428_pos | -0.347263843 | -0.412039913 | -0.344714054 | -0.207756773 | -0.504965272 | 0.449070454 | -0.495485893 | -0.44414271 | -0.704319901 |
| Com_3979_pos | 0.759936869 | 0.487887307 | -0.195067166 | -0.167909222 | 0.498672755 | -0.357545297 | 0.805224059 | 0.010436781 | 0.675576317 |
| Com_5474_pos | 0.77740152 | 0.478043789 | -0.206669981 | -0.185754524 | 0.465308091 | -0.349417966 | 0.762735612 | 0.009722416 | 0.685718435 |
| Com_12496_pos | 0.567394221 | 0.377040165 | -0.133919874 | -0.082429577 | 0.603834647 | -0.431552302 | 0.853162951 | 0.03862961 | 0.669263575 |
| Com_1089_neg | 0.419019508 | 0.463548827 | 0.252623206 | -0.077580618 | 0.557035321 | -0.432439397 | 0.492345286 | 0.321841752 | 0.691660335 |
| Com_20131_pos | -0.570476738 | -0.477996059 | -0.26690488 | -0.139423685 | -0.522790018 | 0.573130556 | -0.641108488 | -0.331863964 | -0.751852172 |
| Com_874_neg | -0.376683568 | -0.290057072 | -0.028229885 | -0.403009135 | -0.454389716 | 0.544862339 | -0.558338738 | -0.189824034 | -0.795883709 |
| Com_337_neg | -0.716380933 | -0.634865976 | -0.256704274 | -0.165641001 | -0.647377179 | 0.542450559 | -0.519963583 | -0.229632115 | -0.720777028 |
| Com_6829_pos | 0.61773649 | 0.55893927 | 0.327015458 | 0.176043334 | 0.464262042 | -0.59512683 | 0.517127396 | 0.487300504 | 0.62257911 |
| Com_1268_neg | 0.529928797 | 0.378311548 | -0.017170918 | -0.165282617 | 0.467848184 | -0.347742489 | 0.693841502 | 0.078307683 | 0.648724043 |
| Com_816_neg | -0.302003067 | -0.191166202 | 0.046894218 | -0.571447754 | -0.437844948 | 0.503971139 | -0.330626814 | -0.064532842 | -0.710361718 |
| Com_4404_neg | 0.368574755 | 0.546232702 | 0.175997858 | 0.032729239 | 0.558779476 | -0.348070259 | 0.621079981 | 0.460345467 | 0.579906714 |
| Com_777_neg | 0.628684521 | 0.414060373 | 0.015523424 | 0.01366245 | 0.511871468 | -0.565567131 | 0.855166548 | 0.078635085 | 0.722398216 |
| Com_2277_pos | 0.11773282 | 0.339916668 | 0.450886234 | 0.286910813 | 0.376031399 | -0.428179459 | 0.420031474 | 0.381466391 | 0.625261811 |
| Com_101_neg | 0.492823054 | 0.513490919 | 0.147441824 | 0.128147843 | 0.377421205 | -0.313443826 | 0.622926361 | 0.294387669 | 0.712859354 |
| Com_1024_neg | 0.634906169 | 0.634454608 | 0.381755041 | 0.19050035 | 0.618650194 | -0.547621182 | 0.502940106 | 0.450993114 | 0.753193065 |

Continued

| ID | Prot_A0A0G2JYV0 | Prot_A0A0G2K0K6 | Prot_A0A0G2K0Q7 | Prot_A0A0G2K0S7 | Prot_A0A0G2K0W1 | Prot_A0A0G2K1D0 | Prot_A0A0G2K1M4 | Prot_A0A0G2K1Q8 | Prot_A0A0G2K1S4 |
| --- | --- | --- | --- | --- | --- | --- | --- | --- | --- |
| Com_4739_neg | -0.472883756 | 0.497112067 | -0.436676284 | -0.613542206 | 0.30165058 | 0.283577698 | 0.572898079 | 0.514442349 | 0.208795202 |
| Com_508_neg | 0.431062865 | -0.5910583 | 0.485714287 | 0.698029264 | -0.503377753 | -0.507923847 | -0.646458469 | -0.713277674 | -0.451605291 |
| Com_3647_pos | -0.405035155 | 0.686119306 | -0.562525678 | -0.500764821 | 0.701537007 | 0.342127861 | 0.440775219 | 0.69254389 | 0.416087556 |
| Com_4991_pos | 0.371389747 | -0.642610017 | 0.570740555 | 0.617671585 | -0.558056932 | -0.476911097 | -0.532162255 | -0.71580205 | -0.258086286 |
| Com_2609_pos | 0.455851212 | -0.690669892 | 0.500339396 | 0.623169847 | -0.539652481 | -0.389106089 | -0.622575876 | -0.711468956 | -0.493883223 |
| Com_586_pos | 0.461462127 | -0.722167125 | 0.504466496 | 0.664218468 | -0.478755279 | -0.488760583 | -0.46043028 | -0.671770179 | -0.286057343 |
| Com_165_neg | -0.483342156 | 0.551911024 | -0.558255562 | -0.475478286 | 0.354183533 | 0.474944842 | 0.731814607 | 0.771956925 | 0.547195311 |
| Com_6688_neg | -0.161908933 | 0.412916199 | -0.695904387 | -0.357706643 | 0.511429827 | 0.191105063 | 0.701837583 | 0.670019487 | 0.385733973 |
| Com_3685_neg | 0.526054549 | -0.571053499 | 0.551161639 | 0.613433403 | -0.550018306 | -0.387949943 | -0.549774443 | -0.59687508 | -0.293702233 |
| Com_313_pos | -0.061922159 | 0.284581228 | -0.681090705 | -0.304107405 | 0.594619182 | 0.142031155 | 0.540263899 | 0.655515078 | 0.300054777 |
| Com_15841_pos | 0.526035556 | -0.444710521 | 0.469879744 | 0.740630018 | -0.439039159 | -0.383401433 | -0.531028838 | -0.625114229 | -0.379689479 |
| Com_3856_pos | 0.544902395 | -0.60330971 | 0.5134621 | 0.628036076 | -0.52279204 | -0.368123385 | -0.553712181 | -0.618470539 | -0.387535293 |
| Com_1082_neg | -0.548609421 | 0.516608285 | -0.57881142 | -0.573890646 | 0.39556215 | 0.480247041 | 0.676687732 | 0.731203627 | 0.507627159 |
| Com_646_neg | -0.557562337 | 0.557008001 | -0.334741262 | -0.560892686 | 0.358917996 | 0.231242237 | 0.509135291 | 0.536728282 | 0.080719184 |
| Com_14903_pos | -0.132012986 | 0.359622205 | -0.391507614 | -0.222179523 | 0.263823736 | 0.477300196 | 0.675650162 | 0.723781872 | 0.797051614 |
| Com_2370_pos | 0.569488023 | -0.632481762 | 0.414706539 | 0.806544311 | -0.373489878 | -0.365631344 | -0.511245875 | -0.54665714 | -0.091638366 |
| Com_4083_neg | 0.393928797 | -0.520252679 | 0.471420808 | 0.435409853 | -0.464111448 | -0.396083208 | -0.388273265 | -0.566802901 | -0.085639757 |
| Com_3695_pos | -0.563572439 | 0.607023691 | -0.589164743 | -0.556739107 | 0.476656248 | 0.427737649 | 0.264974899 | 0.428064934 | 0.509121843 |
| Com_12307_pos | 0.352561213 | -0.652718713 | 0.560437609 | 0.556732856 | -0.509009699 | -0.317639774 | -0.579625217 | -0.754395878 | -0.386796162 |
| Com_8577_pos | -0.351786779 | 0.649524774 | -0.717807141 | -0.431218276 | 0.597564756 | 0.402974951 | 0.69902884 | 0.872969779 | 0.606175215 |
| Com_24285_pos | -0.248412625 | 0.510625068 | -0.700259925 | -0.485771393 | 0.685938228 | 0.275031188 | 0.581373772 | 0.602941107 | 0.273664224 |
| Com_6860_pos | 0.64970213 | -0.61781229 | 0.211405717 | 0.676525486 | -0.229275285 | -0.530780894 | -0.309260611 | -0.407863621 | -0.233580427 |
| Com_2781_pos | 0.249753336 | -0.530338726 | 0.343557097 | 0.591633662 | -0.413643921 | -0.213893333 | -0.643655366 | -0.692224496 | -0.183749681 |
| Com_6127_pos | 0.039886516 | -0.649503641 | 0.300078632 | 0.634662932 | -0.35335438 | -0.483992538 | -0.655223125 | -0.692181015 | -0.289417421 |
| Com_842_neg | -0.442719591 | 0.578362571 | -0.467999965 | -0.399186465 | 0.472826411 | 0.468071015 | 0.527697108 | 0.616416399 | 0.801046993 |
| Com_5298_pos | -0.50733849 | 0.623227583 | -0.435411326 | -0.655469679 | 0.410382991 | 0.625470062 | 0.472394014 | 0.786005337 | 0.539591021 |
| Com_1241_neg | 0.37509999 | -0.30631226 | 0.301176707 | 0.483588048 | -0.35754016 | -0.258051287 | -0.350393539 | -0.346947594 | -0.306461758 |
| Com_124_pos | -0.28360113 | 0.660684895 | -0.683161581 | -0.313293409 | 0.660556601 | 0.413044451 | 0.574184969 | 0.781221052 | 0.562882869 |
| Com_20491_pos | -0.121437914 | 0.704815132 | -0.419752859 | -0.511769763 | 0.626630719 | 0.119648314 | 0.733389354 | 0.719179026 | 0.352706575 |
| Com_941_neg | 0.521428953 | -0.670216939 | 0.641563536 | 0.545542785 | -0.526393354 | -0.434958557 | -0.320137782 | -0.539728734 | -0.265230228 |
| Com_8285_pos | -0.637311525 | 0.591418252 | -0.574792778 | -0.66289167 | 0.649851634 | 0.42322693 | 0.542245154 | 0.628118064 | 0.52769275 |
| Com_2128_neg | -0.487566026 | 0.398850012 | -0.37755347 | -0.38130301 | 0.295925242 | 0.772101598 | 0.311986126 | 0.59258813 | 0.551654106 |
| Com_4694_pos | 0.363812541 | -0.555426895 | 0.319769068 | 0.705825155 | -0.44508139 | -0.57578588 | -0.507410172 | -0.753943823 | -0.496215687 |
| Com_1660_neg | 0.473911954 | -0.598461519 | 0.472505475 | 0.487239811 | -0.510034952 | -0.371140408 | -0.378832644 | -0.528887948 | -0.331942328 |
| Com_11428_pos | 0.371431349 | -0.436556171 | 0.604034843 | 0.407154462 | -0.429969814 | -0.35733905 | -0.652565366 | -0.694515511 | -0.506714969 |
| Com_3979_pos | -0.261731767 | 0.481010717 | -0.358320049 | -0.359664437 | 0.413519387 | 0.499997092 | 0.70321297 | 0.782040122 | 0.807103574 |
| Com_5474_pos | -0.178887483 | 0.454054404 | -0.338507432 | -0.342284185 | 0.373711873 | 0.500102152 | 0.68588966 | 0.772086691 | 0.757859391 |
| Com_12496_pos | -0.154786864 | 0.399025703 | -0.546991288 | -0.189298983 | 0.424361334 | 0.502812485 | 0.591550171 | 0.760643788 | 0.841795861 |
| Com_1089_neg | -0.092263441 | 0.259100281 | -0.547333878 | -0.225144085 | 0.33876557 | 0.271925887 | 0.641582317 | 0.629095648 | 0.531730222 |
| Com_20131_pos | 0.30421088 | -0.519307605 | 0.560532902 | 0.468964578 | -0.427313198 | -0.644007072 | -0.361103065 | -0.637122778 | -0.522217618 |
| Com_874_neg | 0.411513563 | -0.626112654 | 0.601364204 | 0.376704998 | -0.567527858 | -0.480120576 | -0.499927005 | -0.734291312 | -0.516111848 |
| Com_337_neg | 0.334911298 | -0.437746087 | 0.388561361 | 0.580381898 | -0.438497301 | -0.336880903 | -0.630889191 | -0.64364344 | -0.516626353 |
| Com_6829_pos | -0.373186257 | 0.658196017 | -0.413079278 | -0.688409148 | 0.590114677 | 0.402386093 | 0.559620687 | 0.751763951 | 0.455036801 |
| Com_1268_neg | -0.089406053 | 0.248708954 | -0.455555337 | -0.149085739 | 0.298607421 | 0.414958147 | 0.594228856 | 0.644041859 | 0.734638585 |
| Com_816_neg | 0.363349569 | -0.542579932 | 0.516945888 | 0.410561238 | -0.501714717 | -0.414092139 | -0.348036438 | -0.57662181 | -0.235143791 |
| Com_4404_neg | -0.57084866 | 0.697055456 | -0.507965123 | -0.469650426 | 0.484655878 | 0.395123046 | 0.595082318 | 0.769649322 | 0.566631694 |
| Com_777_neg | -0.297781886 | 0.464167248 | -0.574888195 | -0.437382394 | 0.40352786 | 0.57128481 | 0.714412881 | 0.743413806 | 0.815852497 |
| Com_2277_pos | -0.515576412 | 0.369958839 | -0.553135025 | -0.496137942 | 0.384097225 | 0.617912133 | 0.129252666 | 0.474374186 | 0.331034644 |
| Com_101_neg | -0.440634219 | 0.454166671 | -0.443894236 | -0.29270354 | 0.284400491 | 0.465582448 | 0.639552212 | 0.658243007 | 0.61567935 |
| Com_1024_neg | -0.431492991 | 0.535992244 | -0.491166222 | -0.447672817 | 0.531350993 | 0.322498286 | 0.678930325 | 0.62632808 | 0.485837446 |

Continued

| ID | Prot_A0A0G2K1W9 | Prot_A0A0G2K2B0 | Prot_A0A0G2K2B4 | Prot_A0A0G2K2K3 | Prot_A0A0G2K2U7 | Prot_A0A0G2K2X9 | Prot_A0A0G2K394 | Prot_A0A0G2K3H2 | Prot_A0A0G2K3H5 |
| --- | --- | --- | --- | --- | --- | --- | --- | --- | --- |
| Com_4739_neg | 0.523843002 | 0.308184128 | -0.357629005 | 0.481101876 | -0.467366217 | -0.055836156 | -0.385045133 | -0.329169718 | 0.619099776 |
| Com_508_neg | -0.60989207 | -0.416739955 | 0.39940747 | -0.611181861 | 0.513106892 | -0.164587804 | 0.430403584 | 0.472841969 | -0.659609702 |
| Com_3647_pos | 0.706459834 | 0.489664537 | -0.438041824 | 0.430929343 | -0.551925237 | 0.097491377 | -0.625416929 | -0.555528575 | 0.575639914 |
| Com_4991_pos | -0.769631248 | -0.501490417 | 0.40846235 | -0.471243412 | 0.556809644 | -0.113051905 | 0.553693273 | 0.567741872 | -0.61696448 |
| Com_2609_pos | -0.544043386 | -0.382294435 | 0.600916746 | -0.424928483 | 0.528391181 | -0.104484082 | 0.442857756 | 0.363213241 | -0.692888275 |
| Com_586_pos | -0.668972484 | -0.395968431 | 0.576290909 | -0.413652833 | 0.613630874 | -0.023894171 | 0.455118594 | 0.518876927 | -0.682474065 |
| Com_165_neg | 0.472094899 | 0.349506623 | -0.578805855 | 0.546588519 | -0.437250248 | 0.129212671 | -0.428017001 | -0.264279851 | 0.676197663 |
| Com_6688_neg | 0.388556328 | 0.308896358 | -0.444106837 | 0.279138633 | -0.27953438 | 0.036439449 | -0.608965912 | -0.082169413 | 0.460439659 |
| Com_3685_neg | -0.540493366 | -0.222775832 | 0.505231 | -0.496591874 | 0.54144449 | 0.053576435 | 0.440159204 | 0.491347982 | -0.70878008 |
| Com_313_pos | 0.467857225 | 0.319915512 | -0.399285714 | 0.257705905 | -0.405737865 | -0.145897622 | -0.821338539 | -0.11084222 | 0.291546844 |
| Com_15841_pos | -0.448580729 | -0.268914702 | 0.446505141 | -0.418222741 | 0.53599153 | 0.025826602 | 0.420379905 | 0.326895482 | -0.56425613 |
| Com_3856_pos | -0.481946459 | -0.31585321 | 0.521438443 | -0.456558157 | 0.494593039 | -0.034235315 | 0.408487357 | 0.412222687 | -0.730257602 |
| Com_1082_neg | 0.544794419 | 0.322167653 | -0.463688137 | 0.522881281 | -0.464886794 | 0.130595048 | -0.442221283 | -0.319599685 | 0.586369232 |
| Com_646_neg | 0.475347874 | 0.382708659 | -0.588101894 | 0.455069375 | -0.591495544 | -0.172355014 | -0.282032105 | -0.605399606 | 0.892169996 |
| Com_14903_pos | 0.363986553 | 0.31662278 | -0.346524329 | 0.553773625 | -0.244152586 | 0.334738883 | -0.449360185 | 0.066680252 | 0.255620525 |
| Com_2370_pos | -0.664598335 | -0.351695603 | 0.56947874 | -0.25737134 | 0.589713919 | 0.127608203 | 0.258194347 | 0.548676297 | -0.706996489 |
| Com_4083_neg | -0.602634383 | -0.243106289 | 0.475932697 | -0.554266978 | 0.626455471 | 0.169695553 | 0.504052188 | 0.622973207 | -0.667302957 |
| Com_3695_pos | 0.677067964 | 0.543846226 | -0.197787407 | 0.022927352 | -0.084636475 | 0.318220571 | -0.214774217 | -0.445214372 | 0.267402361 |
| Com_12307_pos | -0.646569825 | -0.467723195 | 0.574638806 | -0.318955042 | 0.566123492 | -0.023385148 | 0.6271666 | 0.285129817 | -0.556605698 |
| Com_8577_pos | 0.662011607 | 0.551696725 | -0.793191523 | 0.308604798 | -0.462697309 | -0.076868327 | -0.645918403 | -0.362320435 | 0.606201151 |
| Com_24285_pos | 0.520156095 | 0.318918968 | -0.423770178 | 0.253170012 | -0.340014805 | 0.070792142 | -0.546454341 | -0.345791628 | 0.547391722 |
| Com_6860_pos | -0.559600701 | -0.284678507 | 0.497823357 | -0.448857662 | 0.561168656 | -0.019000707 | 0.09932875 | 0.527624897 | -0.693373091 |
| Com_2781_pos | -0.52406564 | -0.325627429 | 0.205514979 | -0.669163 | 0.471625729 | 0.051550851 | 0.472477145 | 0.500068112 | -0.622674439 |
| Com_6127_pos | -0.752901168 | -0.598116223 | 0.232546212 | -0.58014913 | 0.394685116 | -0.232277028 | 0.365558028 | 0.476285501 | -0.525232654 |
| Com_842_neg | 0.547083551 | 0.534110631 | -0.39987678 | 0.463456115 | -0.280170217 | 0.325824558 | -0.377324724 | -0.28504694 | 0.453246361 |
| Com_5298_pos | 0.814172213 | 0.619340598 | -0.431813127 | 0.585586542 | -0.637646621 | 0.155430032 | -0.531232899 | -0.534932932 | 0.509246827 |
| Com_1241_neg | -0.137275173 | -0.030268852 | 0.353211567 | -0.345133817 | 0.360203852 | -0.020095302 | 0.217703933 | 0.181563606 | -0.483226471 |
| Com_124_pos | 0.518182316 | 0.509841884 | -0.642300904 | 0.297720808 | -0.331112063 | 0.207657489 | -0.535226068 | -0.387917827 | 0.63497772 |
| Com_20491_pos | 0.568823465 | 0.429290246 | -0.416191931 | 0.466496029 | -0.432862239 | 0.045742499 | -0.549306968 | -0.358712518 | 0.646678869 |
| Com_941_neg | -0.595234384 | -0.254887306 | 0.491633347 | -0.261949369 | 0.435582769 | 0.010522068 | 0.442867752 | 0.47751256 | -0.562720213 |
| Com_8285_pos | 0.731602945 | 0.589841452 | -0.616340906 | 0.259615388 | -0.48672983 | 0.032080294 | -0.394777396 | -0.573720168 | 0.633104065 |
| Com_2128_neg | 0.691779995 | 0.362410301 | -0.371520373 | 0.549498503 | -0.531744406 | 0.286959697 | -0.426154591 | -0.308722982 | 0.310065159 |
| Com_4694_pos | -0.764472676 | -0.742285169 | 0.360951491 | -0.570319446 | 0.535060216 | -0.173615754 | 0.452690881 | 0.550540012 | -0.547423829 |
| Com_1660_neg | -0.436222469 | -0.296482714 | 0.60848544 | -0.287920482 | 0.489968132 | -0.054129907 | 0.374156284 | 0.367655919 | -0.67132413 |
| Com_11428_pos | -0.559308168 | -0.606218152 | 0.412647054 | -0.4378209 | 0.293498849 | -0.107329234 | 0.609944274 | 0.142297791 | -0.549209032 |
| Com_3979_pos | 0.465537301 | 0.264772818 | -0.412930196 | 0.59815018 | -0.385239647 | 0.318874244 | -0.42976374 | -0.114443393 | 0.35845496 |
| Com_5474_pos | 0.467382899 | 0.241535056 | -0.338196116 | 0.621810843 | -0.348799056 | 0.296489971 | -0.471997508 | -0.043152038 | 0.300841362 |
| Com_12496_pos | 0.412273131 | 0.428641612 | -0.416600339 | 0.39193098 | -0.21059512 | 0.383140063 | -0.51767199 | -0.023583599 | 0.242000572 |
| Com_1089_neg | 0.349182932 | 0.22771821 | -0.32989644 | 0.433283756 | -0.260616305 | 0.036201765 | -0.623664987 | 0.096749353 | 0.241399281 |
| Com_20131_pos | -0.695538056 | -0.603211077 | 0.28616731 | -0.462132108 | 0.273873256 | -0.19597468 | 0.567214924 | 0.291055985 | -0.378673062 |
| Com_874_neg | -0.677907703 | -0.453051469 | 0.712687432 | -0.214837079 | 0.50849152 | -0.128937863 | 0.550125063 | 0.310085463 | -0.51136191 |
| Com_337_neg | -0.318231741 | -0.230009956 | 0.46878398 | -0.441077451 | 0.483097909 | -0.157209164 | 0.371614204 | 0.183203587 | -0.530370243 |
| Com_6829_pos | 0.723878999 | 0.567898343 | -0.541487482 | 0.440387643 | -0.544425381 | -0.069417735 | -0.483036569 | -0.584155053 | 0.616376485 |
| Com_1268_neg | 0.3474064 | 0.246186276 | -0.34387465 | 0.427892099 | -0.226293358 | 0.226640174 | -0.539372041 | 0.169933713 | 0.130929872 |
| Com_816_neg | -0.533911554 | -0.33576722 | 0.517760373 | -0.1089213 | 0.424951289 | -0.204586242 | 0.396114363 | 0.328993197 | -0.494115156 |
| Com_4404_neg | 0.648081428 | 0.63315437 | -0.508440201 | 0.528287683 | -0.428144414 | 0.135369116 | -0.430098749 | -0.582511155 | 0.724699333 |
| Com_777_neg | 0.48260154 | 0.455987577 | -0.3639145 | 0.414580337 | -0.147954659 | 0.414932123 | -0.350711336 | -0.137387504 | 0.353995496 |
| Com_2277_pos | 0.788435367 | 0.594351304 | -0.482214508 | 0.135861208 | -0.533633322 | 0.025387485 | -0.501150592 | -0.427493027 | 0.274444696 |
| Com_101_neg | 0.422584414 | 0.368931953 | -0.617773537 | 0.478610413 | -0.401128467 | 0.174936963 | -0.415854319 | -0.054129447 | 0.581012941 |
| Com_1024_neg | 0.435323877 | 0.308124139 | -0.537279392 | 0.599819518 | -0.458290856 | 0.034632693 | -0.480155294 | -0.289949186 | 0.746510111 |

Continued

| ID | Prot_A0A0G2K4G0 | Prot_A0A0G2K4M7 | Prot_A0A0G2K5D5 | Prot_A0A0G2K613 | Prot_A0A0G2K707 | Prot_A0A0G2K7B9 | Prot_A0A0G2K7G2 | Prot_A0A0G2K7I9 | Prot_A0A0G2K7K2 |
| --- | --- | --- | --- | --- | --- | --- | --- | --- | --- |
| Com_4739_neg | -0.338098447 | -0.56284611 | 0.338540055 | -0.40797727 | 0.550900977 | -0.264497565 | 0.427444004 | 0.663121723 | 0.375334503 |
| Com_508_neg | 0.591895762 | 0.749105095 | -0.633005769 | 0.677338816 | -0.741478338 | 0.476810796 | -0.685744877 | -0.652833748 | -0.674693486 |
| Com_3647_pos | -0.649068305 | -0.618124792 | 0.583929022 | -0.534145245 | 0.580961235 | -0.51917427 | 0.530467828 | 0.654101487 | 0.710818191 |
| Com_4991_pos | 0.476919342 | 0.678934934 | -0.425656081 | 0.472160797 | -0.625126055 | 0.477541074 | -0.603830197 | -0.748112933 | -0.641392077 |
| Com_2609_pos | 0.632648912 | 0.617315143 | -0.739062307 | 0.695463641 | -0.816883312 | 0.505789782 | -0.599820517 | -0.566628002 | -0.747461862 |
| Com_586_pos | 0.483718616 | 0.646619635 | -0.602347277 | 0.560851263 | -0.771195339 | 0.399233247 | -0.515733063 | -0.6554587 | -0.723252693 |
| Com_165_neg | -0.628012755 | -0.657094043 | 0.463327239 | -0.706183867 | 0.702578525 | -0.382546224 | 0.690469793 | 0.522901418 | 0.579694961 |
| Com_6688_neg | -0.464658948 | -0.324669105 | 0.596249616 | -0.500017823 | 0.608301763 | -0.358448627 | 0.469282263 | 0.664568076 | 0.406347643 |
| Com_3685_neg | 0.449280782 | 0.688230982 | -0.552901565 | 0.601800687 | -0.680123745 | 0.406836652 | -0.689931792 | -0.644817544 | -0.636164115 |
| Com_313_pos | -0.367999449 | -0.328401331 | 0.479418544 | -0.383761882 | 0.509678568 | -0.409154265 | 0.391476511 | 0.712768587 | 0.376409862 |
| Com_15841_pos | 0.472190118 | 0.685466465 | -0.680468334 | 0.696974308 | -0.785534968 | 0.496798796 | -0.573361823 | -0.599347593 | -0.697834208 |
| Com_3856_pos | 0.556257404 | 0.669076819 | -0.710328831 | 0.672116643 | -0.743862463 | 0.420488796 | -0.622700046 | -0.590819296 | -0.720398949 |
| Com_1082_neg | -0.585187378 | -0.665669003 | 0.425910749 | -0.653474618 | 0.659925437 | -0.455291607 | 0.68945874 | 0.582469187 | 0.556885005 |
| Com_646_neg | -0.317766379 | -0.815188157 | 0.246566037 | -0.537745431 | 0.569114131 | -0.287904804 | 0.519666707 | 0.518874738 | 0.649428486 |
| Com_14903_pos | -0.724138995 | -0.369028308 | 0.435713033 | -0.589375859 | 0.54187936 | -0.38798982 | 0.567119205 | 0.310017864 | 0.329357207 |
| Com_2370_pos | 0.145281611 | 0.667851415 | -0.330903853 | 0.453364826 | -0.669727085 | 0.451358004 | -0.519852433 | -0.528479867 | -0.650024295 |
| Com_4083_neg | 0.333771783 | 0.72151395 | -0.197574622 | 0.426003675 | -0.502875022 | 0.265634066 | -0.592021781 | -0.672276549 | -0.521162023 |
| Com_3695_pos | -0.339439796 | -0.257030205 | 0.622909683 | -0.314791408 | 0.261283009 | -0.326935019 | 0.268377173 | 0.176316013 | 0.509869161 |
| Com_12307_pos | 0.543920964 | 0.513406499 | -0.535436551 | 0.521677572 | -0.731293632 | 0.521457852 | -0.450267739 | -0.682324798 | -0.637129213 |
| Com_8577_pos | -0.462326081 | -0.559160733 | 0.575631553 | -0.660358022 | 0.642642346 | -0.454188248 | 0.578709088 | 0.392641217 | 0.698175503 |
| Com_24285_pos | -0.41083858 | -0.425385893 | 0.689402306 | -0.47718274 | 0.619649186 | -0.440834046 | 0.593885334 | 0.677688404 | 0.573948121 |
| Com_6860_pos | 0.322923247 | 0.671194097 | -0.394610861 | 0.449260978 | -0.599980997 | 0.241413831 | -0.483824197 | -0.33024846 | -0.597216876 |
| Com_2781_pos | 0.426040357 | 0.794173703 | -0.228594405 | 0.616827515 | -0.577448025 | 0.531102178 | -0.645704603 | -0.782508816 | -0.576542788 |
| Com_6127_pos | 0.359240075 | 0.593980637 | -0.349333288 | 0.409102245 | -0.575182415 | 0.417288929 | -0.501263904 | -0.611656778 | -0.480496906 |
| Com_842_neg | -0.725237674 | -0.447840302 | 0.703769734 | -0.563721575 | 0.46679695 | -0.311641602 | 0.444129351 | 0.233102867 | 0.512076341 |
| Com_5298_pos | -0.614656767 | -0.775220679 | 0.40133714 | -0.574242324 | 0.611190258 | -0.481461579 | 0.516796713 | 0.537218402 | 0.670838835 |
| Com_1241_neg | 0.455083565 | 0.448502997 | -0.753180495 | 0.57968544 | -0.663122886 | 0.256069658 | -0.435015194 | -0.414069948 | -0.520254747 |
| Com_124_pos | -0.671196646 | -0.506382456 | 0.766548535 | -0.673936198 | 0.654331475 | -0.415544382 | 0.610367913 | 0.432866324 | 0.77444481 |
| Com_20491_pos | -0.536155279 | -0.547268804 | 0.539094334 | -0.574023048 | 0.672309341 | -0.608636078 | 0.584409203 | 0.692176078 | 0.63126111 |
| Com_941_neg | 0.395294736 | 0.46322315 | -0.647810547 | 0.46921932 | -0.608915415 | 0.295230566 | -0.471381524 | -0.582556525 | -0.630701774 |
| Com_8285_pos | -0.384342034 | -0.660713514 | 0.590305773 | -0.550135288 | 0.519478588 | -0.502662406 | 0.584615156 | 0.262158358 | 0.733513454 |
| Com_2128_neg | -0.576193058 | -0.574187304 | 0.192017087 | -0.356975046 | 0.437788485 | -0.36925994 | 0.639734156 | 0.324511662 | 0.424694969 |
| Com_4694_pos | 0.503295028 | 0.827901915 | -0.504133132 | 0.599021487 | -0.602814516 | 0.534453532 | -0.559437449 | -0.433569234 | -0.766530104 |
| Com_1660_neg | 0.532938449 | 0.518076765 | -0.739221043 | 0.555170813 | -0.714233958 | 0.329404947 | -0.493881946 | -0.471242073 | -0.711680001 |
| Com_11428_pos | 0.498810547 | 0.506801018 | -0.493940903 | 0.457812018 | -0.483367272 | 0.342039122 | -0.525985886 | -0.514974638 | -0.475200676 |
| Com_3979_pos | -0.771152595 | -0.55232272 | 0.416228488 | -0.714479985 | 0.639860177 | -0.645362217 | 0.802374924 | 0.325727578 | 0.541272115 |
| Com_5474_pos | -0.71476289 | -0.510145847 | 0.37643785 | -0.659056211 | 0.622442867 | -0.641891415 | 0.803497335 | 0.374632627 | 0.490370526 |
| Com_12496_pos | -0.766954235 | -0.33189217 | 0.620823482 | -0.612408526 | 0.533839274 | -0.401699062 | 0.530325387 | 0.268422427 | 0.466647253 |
| Com_1089_neg | -0.478513228 | -0.264729016 | 0.406647217 | -0.426169192 | 0.474912248 | -0.288303134 | 0.437441174 | 0.523136247 | 0.193263105 |
| Com_20131_pos | 0.452691579 | 0.462093894 | -0.679109367 | 0.404079245 | -0.475432991 | 0.252772359 | -0.465476143 | -0.446596912 | -0.54227764 |
| Com_874_neg | 0.537133032 | 0.467904541 | -0.450918508 | 0.480749954 | -0.612940832 | 0.532984791 | -0.617258718 | -0.370596358 | -0.67072175 |
| Com_337_neg | 0.667829266 | 0.570722255 | -0.74445422 | 0.736235017 | -0.834222578 | 0.502191171 | -0.564037917 | -0.538854399 | -0.631819002 |
| Com_6829_pos | -0.377752538 | -0.768021904 | 0.554244412 | -0.665400222 | 0.643541061 | -0.603208162 | 0.617729536 | 0.405535614 | 0.830793651 |
| Com_1268_neg | -0.601967401 | -0.227104911 | 0.411334768 | -0.452471084 | 0.461734862 | -0.351132512 | 0.496663442 | 0.301420101 | 0.221416259 |
| Com_816_neg | 0.457068436 | 0.431891134 | -0.456887551 | 0.409344881 | -0.618890669 | 0.505844909 | -0.57092333 | -0.474666549 | -0.684095025 |
| Com_4404_neg | -0.646923983 | -0.771051599 | 0.397255849 | -0.673773891 | 0.483293892 | -0.41738393 | 0.580436069 | 0.399015745 | 0.717847725 |
| Com_777_neg | -0.638805368 | -0.426533028 | 0.675004408 | -0.664837395 | 0.572728952 | -0.427908749 | 0.633073151 | 0.26656332 | 0.506981606 |
| Com_2277_pos | -0.234379326 | -0.411913997 | 0.38121349 | -0.150579785 | 0.340877513 | -0.171380062 | 0.192141303 | 0.296240641 | 0.428138619 |
| Com_101_neg | -0.629346479 | -0.491615454 | 0.39392443 | -0.52954547 | 0.587726427 | -0.308131079 | 0.610670134 | 0.318084889 | 0.445626567 |
| Com_1024_neg | -0.615930472 | -0.631628972 | 0.618274495 | -0.630614788 | 0.658697903 | -0.360701342 | 0.691583243 | 0.551843948 | 0.566455537 |

Continued

| ID | Prot_A0A0G2K7N8 | Prot_A0A0G2K7P9 | Prot_A0A0G2K7W2 | Prot_A0A0G2K808 | Prot_A0A0G2K8K1 | Prot_A0A0G2K8M3 | Prot_A0A0G2K8Q2 | Prot_A0A0G2K8R3 | Prot_A0A0G2K948 |
| --- | --- | --- | --- | --- | --- | --- | --- | --- | --- |
| Com_4739_neg | 0.245132633 | -0.392437539 | 0.113049983 | 0.613253867 | 0.19038373 | 0.304605776 | -0.145824815 | -0.663085103 | 0.398458127 |
| Com_508_neg | -0.398576463 | 0.653045665 | -0.463661299 | -0.549436701 | -0.191138382 | -0.360920542 | -0.153630117 | 0.66264637 | -0.576844728 |
| Com_3647_pos | 0.309761334 | -0.439194181 | 0.45530762 | 0.519628805 | 0.041167445 | 0.354438849 | 0.218906076 | -0.490009695 | 0.456929013 |
| Com_4991_pos | -0.362442463 | 0.493294741 | -0.293677198 | -0.491764523 | -0.065847604 | -0.28483991 | -0.288128532 | 0.677159996 | -0.349504283 |
| Com_2609_pos | -0.233895741 | 0.71603608 | -0.419107909 | -0.560929944 | -0.061688018 | -0.265915126 | -0.273375411 | 0.473811272 | -0.586120692 |
| Com_586_pos | -0.307992668 | 0.615068475 | -0.419283398 | -0.53239951 | -0.057348796 | -0.287249155 | -0.186295408 | 0.45247303 | -0.43722956 |
| Com_165_neg | 0.253818408 | -0.636285496 | 0.280106637 | 0.285981716 | 0.243489328 | 0.056061001 | 0.155895306 | -0.547990182 | 0.696740559 |
| Com_6688_neg | 0.064981333 | -0.51233884 | 0.054056104 | 0.542913469 | 0.085467112 | 0.238880117 | 0.236529553 | -0.567351591 | 0.446978971 |
| Com_3685_neg | -0.371131866 | 0.578232097 | -0.337566665 | -0.525743851 | -0.109323495 | -0.294105711 | -0.121034558 | 0.505665368 | -0.458648784 |
| Com_313_pos | 0.088259536 | -0.388272434 | 0.111917316 | 0.58616666 | 0.15241176 | 0.215035225 | 0.165354803 | -0.503451297 | 0.397296129 |
| Com_15841_pos | -0.400511003 | 0.647961225 | -0.433208138 | -0.610167211 | -0.204732627 | -0.277450405 | -0.072415475 | 0.520836739 | -0.535035175 |
| Com_3856_pos | -0.346775081 | 0.614533025 | -0.383491017 | -0.553141355 | -0.074554875 | -0.321852093 | -0.148759545 | 0.436313186 | -0.522327733 |
| Com_1082_neg | 0.329435964 | -0.569615107 | 0.285731738 | 0.382788627 | 0.295983836 | 0.107488515 | 0.118076757 | -0.699366882 | 0.634558614 |
| Com_646_neg | 0.290566082 | -0.460482123 | 0.190122068 | 0.346133506 | -0.159076966 | 0.238954859 | 0.033613055 | -0.338360918 | 0.286669133 |
| Com_14903_pos | 0.083804872 | -0.568330305 | 0.367497604 | 0.168369889 | 0.490677444 | -0.063218455 | 0.177896589 | -0.517744326 | 0.855874605 |
| Com_2370_pos | -0.357185978 | 0.584638006 | -0.166494286 | -0.577956842 | 0.083314376 | -0.122815673 | -0.234751168 | 0.570786814 | -0.189991193 |
| Com_4083_neg | -0.346535732 | 0.389129293 | -0.302405168 | -0.284999526 | -0.097313265 | -0.224128144 | -0.010779131 | 0.379860657 | -0.328316566 |
| Com_3695_pos | 0.402168888 | -0.203696046 | 0.326512854 | 0.57466834 | 0.002722207 | 0.245710012 | 0.175963103 | -0.557735747 | 0.27793895 |
| Com_12307_pos | -0.162885371 | 0.569963694 | -0.245449898 | -0.57229478 | -0.094564021 | -0.155458053 | -0.267790751 | 0.550063733 | -0.47441497 |
| Com_8577_pos | 0.23634952 | -0.653667759 | 0.358085948 | 0.45121859 | 0.039593503 | -0.019402608 | 0.302010157 | -0.367773568 | 0.620684229 |
| Com_24285_pos | 0.211785036 | -0.536027189 | 0.149142278 | 0.604685422 | -0.080050775 | 0.355064392 | 0.433979495 | -0.559693728 | 0.289082987 |
| Com_6860_pos | -0.327822337 | 0.501004207 | -0.39642541 | -0.418030001 | -0.092696717 | -0.193922181 | -0.004700904 | 0.353533592 | -0.425485399 |
| Com_2781_pos | -0.644277798 | 0.502346567 | -0.355037965 | -0.304184656 | -0.225877423 | -0.144527578 | -0.029701808 | 0.521113258 | -0.27663806 |
| Com_6127_pos | -0.369537985 | 0.570916326 | -0.335022978 | -0.471927019 | -0.117772837 | -0.245276332 | -0.256024118 | 0.69354087 | -0.304781756 |
| Com_842_neg | 0.144136105 | -0.434092083 | 0.501238448 | 0.503397368 | 0.211399783 | 0.313521076 | 0.031765511 | -0.501489817 | 0.794572498 |
| Com_5298_pos | 0.399739239 | -0.485432586 | 0.569880538 | 0.445712911 | 0.315556027 | 0.187267196 | 0.020948654 | -0.644822623 | 0.662765008 |
| Com_1241_neg | -0.163319251 | 0.551879315 | -0.399996578 | -0.487484454 | -0.102656194 | -0.410863481 | -0.019317165 | 0.233962439 | -0.48568214 |
| Com_124_pos | 0.195569922 | -0.620393067 | 0.393796969 | 0.2924218 | -0.112521469 | 0.186956464 | 0.517007986 | -0.265154924 | 0.539297204 |
| Com_20491_pos | 0.297879662 | -0.634960366 | 0.280352848 | 0.536407012 | -0.011609982 | 0.214180998 | 0.365492791 | -0.483594192 | 0.333446442 |
| Com_941_neg | -0.378998381 | 0.448938599 | -0.360536399 | -0.510609743 | -0.07468242 | -0.260537232 | -0.13027239 | 0.343545265 | -0.34573688 |
| Com_8285_pos | 0.278730063 | -0.519081771 | 0.35812209 | 0.669726916 | -0.111869797 | 0.238011293 | 0.288391872 | -0.581046546 | 0.501218599 |
| Com_2128_neg | 0.208258959 | -0.370112671 | 0.383735707 | 0.162943918 | 0.46230534 | -0.055070493 | 0.207618953 | -0.608378292 | 0.720128795 |
| Com_4694_pos | -0.516460569 | 0.554961778 | -0.58984807 | -0.481472835 | -0.161639514 | -0.203544394 | -0.201597933 | 0.540683693 | -0.542597049 |
| Com_1660_neg | -0.142075696 | 0.573549802 | -0.352164878 | -0.476904221 | 0.069452626 | -0.312510601 | -0.279222115 | 0.244733639 | -0.467553688 |
| Com_11428_pos | -0.267725698 | 0.373076646 | -0.088877021 | -0.517452774 | -0.212730388 | -0.097085382 | -0.160301169 | 0.565695367 | -0.576358366 |
| Com_3979_pos | 0.262696938 | -0.713022915 | 0.489994322 | 0.128377293 | 0.448053209 | -0.166313697 | 0.391566902 | -0.537776727 | 0.820559733 |
| Com_5474_pos | 0.342438445 | -0.688816624 | 0.456008907 | 0.111773307 | 0.541614522 | -0.248067199 | 0.393306667 | -0.510135589 | 0.772845427 |
| Com_12496_pos | 0.065406771 | -0.547084071 | 0.428053494 | 0.199477918 | 0.317825864 | 0.026055699 | 0.327241767 | -0.443622995 | 0.82386413 |
| Com_1089_neg | 0.052734131 | -0.419734892 | 0.13623552 | 0.417467186 | 0.431836863 | 0.060824269 | 0.005029506 | -0.541107999 | 0.651282386 |
| Com_20131_pos | -0.496397599 | 0.365846103 | -0.449972646 | -0.507593276 | -0.353629177 | -0.160511744 | -0.112346535 | 0.388157365 | -0.565810484 |
| Com_874_neg | -0.091379621 | 0.573864471 | -0.233284251 | -0.313239894 | -0.013410867 | 0.093629836 | -0.591813088 | 0.435212283 | -0.531116457 |
| Com_337_neg | -0.102785328 | 0.747594663 | -0.431635494 | -0.516864147 | -0.145642539 | -0.342412246 | -0.211625472 | 0.526463524 | -0.656868264 |
| Com_6829_pos | 0.561948877 | -0.66202181 | 0.582507211 | 0.521895288 | 0.046107382 | 0.086032052 | 0.264834132 | -0.357656437 | 0.444122491 |
| Com_1268_neg | -0.005830107 | -0.47440303 | 0.249265385 | 0.251630667 | 0.500305095 | -0.0918168 | 0.175377649 | -0.499715198 | 0.800719799 |
| Com_816_neg | -0.158296043 | 0.514156113 | -0.135740632 | -0.22877453 | 0.125196572 | -0.014701389 | -0.709442332 | 0.387692967 | -0.242730891 |
| Com_4404_neg | 0.436266128 | -0.42854715 | 0.440020219 | 0.267737397 | 0.063753176 | 0.132278963 | 0.076061148 | -0.438434403 | 0.578255111 |
| Com_777_neg | 0.260295225 | -0.626275044 | 0.403103645 | 0.348518795 | 0.290013581 | 0.075125812 | 0.320956485 | -0.632395434 | 0.746007966 |
| Com_2277_pos | 0.03829213 | -0.180344789 | 0.260466334 | 0.619728559 | 0.08475097 | 0.27336309 | 0.030266358 | -0.561038291 | 0.464380036 |
| Com_101_neg | 0.000803785 | -0.536773443 | 0.145910942 | 0.253693684 | 0.255452399 | -0.060877876 | 0.231923799 | -0.458635801 | 0.782587607 |
| Com_1024_neg | 0.195786257 | -0.585789645 | 0.288701868 | 0.510915528 | 0.148218779 | 0.284816833 | 0.115710194 | -0.453241179 | 0.66201011 |

Continued

| ID | Prot_A0A0G2K952 | Prot_A0A0G2K9C0 | Prot_A0A0G2K9C8 | Prot_A0A0G2K9G3 | Prot_A0A0G2K9I6 | Prot_A0A0G2KBB3 | Prot_A0A0H2UHA8 | Prot_A0A0H2UHL2 | Prot_A0A0H2UHM0 |
| --- | --- | --- | --- | --- | --- | --- | --- | --- | --- |
| Com_4739_neg | -0.57037661 | 0.519796147 | 0.354316819 | 0.670942426 | 0.357366391 | -0.039701237 | 0.334367782 | 0.424071539 | -0.681349089 |
| Com_508_neg | 0.501243169 | -0.712575151 | -0.595210605 | -0.689264841 | -0.534451996 | -0.280274725 | -0.548689937 | -0.4663084 | 0.775198506 |
| Com_3647_pos | -0.666386923 | 0.737966142 | 0.540798122 | 0.735593238 | 0.37559111 | 0.488588665 | 0.617610447 | 0.316892141 | -0.642862954 |
| Com_4991_pos | 0.637797455 | -0.605129511 | -0.400576432 | -0.637507867 | -0.359894477 | -0.336053115 | -0.51501096 | -0.517126918 | 0.664699406 |
| Com_2609_pos | 0.547700911 | -0.778618762 | -0.582309981 | -0.66935646 | -0.516655427 | -0.297910053 | -0.615713133 | -0.38042076 | 0.680989852 |
| Com_586_pos | 0.706723818 | -0.720650681 | -0.459352213 | -0.638053252 | -0.367020883 | -0.336040052 | -0.529521481 | -0.445052069 | 0.633287348 |
| Com_165_neg | -0.354080112 | 0.611984302 | 0.655136712 | 0.647017429 | 0.705991868 | 0.088469536 | 0.348223724 | 0.535587482 | -0.566887077 |
| Com_6688_neg | -0.289298941 | 0.525105951 | 0.452278229 | 0.693078472 | 0.524165301 | 0.157460403 | 0.255043211 | 0.29004186 | -0.481889436 |
| Com_3685_neg | 0.666504304 | -0.709836641 | -0.441778322 | -0.65066261 | -0.38813968 | -0.204233336 | -0.53925662 | -0.467337851 | 0.627801245 |
| Com_313_pos | -0.350588887 | 0.532855685 | 0.327718844 | 0.638007642 | 0.461915949 | 0.169265601 | 0.218197859 | 0.188902154 | -0.389451259 |
| Com_15841_pos | 0.568098934 | -0.807187276 | -0.491783239 | -0.528433551 | -0.435854922 | -0.145772276 | -0.54713502 | -0.413291643 | 0.646526432 |
| Com_3856_pos | 0.623343734 | -0.761802436 | -0.525215284 | -0.642353701 | -0.40587361 | -0.26513026 | -0.576103125 | -0.414879025 | 0.687291203 |
| Com_1082_neg | -0.453157704 | 0.677784075 | 0.619430786 | 0.622137174 | 0.639815516 | 0.061249589 | 0.438402158 | 0.567330974 | -0.591916091 |
| Com_646_neg | -0.632614481 | 0.404873856 | 0.195697268 | 0.53573833 | 0.173697871 | 0.135830197 | 0.355960823 | 0.44487528 | -0.628448048 |
| Com_14903_pos | 0.079784625 | 0.523646067 | 0.806543677 | 0.558362911 | 0.947401716 | 0.068263401 | 0.246277602 | 0.313013806 | -0.41838811 |
| Com_2370_pos | 0.706555446 | -0.540589006 | -0.103285311 | -0.357640997 | -0.163983278 | 0.003699104 | -0.553199537 | -0.456768691 | 0.536854772 |
| Com_4083_neg | 0.675668159 | -0.486161664 | -0.300543237 | -0.636184239 | -0.288532151 | -0.248899238 | -0.313126582 | -0.492573954 | 0.504011698 |
| Com_3695_pos | -0.43319754 | 0.56279995 | 0.432474423 | 0.303214327 | 0.211822751 | 0.240386072 | 0.523441114 | 0.036213117 | -0.318425844 |
| Com_12307_pos | 0.580581092 | -0.698471709 | -0.45755344 | -0.653737208 | -0.472085177 | -0.220832578 | -0.509183327 | -0.389108356 | 0.58919659 |
| Com_8577_pos | -0.330419137 | 0.580586443 | 0.491552587 | 0.57474751 | 0.654821992 | 0.166788441 | 0.333776428 | 0.147970807 | -0.319825152 |
| Com_24285_pos | -0.498838133 | 0.601453468 | 0.339957137 | 0.604015712 | 0.316719387 | 0.340130924 | 0.490869389 | 0.333718271 | -0.540914318 |
| Com_6860_pos | 0.64998221 | -0.57696862 | -0.336793541 | -0.376225149 | -0.269354267 | -0.082167535 | -0.499476003 | -0.429830372 | 0.592720413 |
| Com_2781_pos | 0.509565185 | -0.528953849 | -0.406750042 | -0.675632031 | -0.320107481 | -0.205221038 | -0.518194438 | -0.477669406 | 0.641175589 |
| Com_6127_pos | 0.276750269 | -0.352674678 | -0.363878685 | -0.583874942 | -0.421192744 | -0.247055373 | -0.413223183 | -0.327101354 | 0.679585588 |
| Com_842_neg | -0.221322428 | 0.626702177 | 0.775951973 | 0.653113745 | 0.693942446 | 0.26042862 | 0.403258373 | 0.091314151 | -0.5867618 |
| Com_5298_pos | -0.525204048 | 0.692914651 | 0.626147772 | 0.602384892 | 0.597770244 | 0.241634874 | 0.464930982 | 0.41462349 | -0.654382337 |
| Com_1241_neg | 0.409346986 | -0.671690648 | -0.468102723 | -0.489697878 | -0.332544886 | -0.240749616 | -0.414614818 | -0.264423952 | 0.567122964 |
| Com_124_pos | -0.338127959 | 0.604575905 | 0.600673596 | 0.604244835 | 0.527272233 | 0.535562865 | 0.428618236 | 0.258383091 | -0.434718816 |
| Com_20491_pos | -0.450311786 | 0.584766976 | 0.412293125 | 0.738766227 | 0.385143314 | 0.322012357 | 0.658043974 | 0.264373171 | -0.667427929 |
| Com_941_neg | 0.749391676 | -0.738735507 | -0.445757277 | -0.579543025 | -0.261566154 | -0.329732321 | -0.505323824 | -0.349687027 | 0.43205268 |
| Com_8285_pos | -0.496365535 | 0.609488167 | 0.332617359 | 0.39599672 | 0.399634777 | 0.136784908 | 0.563319815 | 0.158025844 | -0.525652989 |
| Com_2128_neg | -0.374443985 | 0.589365448 | 0.581713348 | 0.330759444 | 0.696547071 | 0.077577777 | 0.380481061 | 0.582315892 | -0.449388458 |
| Com_4694_pos | 0.375520187 | -0.581429171 | -0.469584997 | -0.421563907 | -0.476928261 | -0.284008869 | -0.516522604 | -0.279550536 | 0.683393787 |
| Com_1660_neg | 0.60841031 | -0.70181859 | -0.446583563 | -0.53571642 | -0.326029145 | -0.371972017 | -0.513275756 | -0.348614685 | 0.57259951 |
| Com_11428_pos | 0.312728152 | -0.503097328 | -0.467654868 | -0.541128468 | -0.564145485 | -0.023146318 | -0.292236034 | -0.313724922 | 0.57338948 |
| Com_3979_pos | -0.096658245 | 0.682561336 | 0.787801817 | 0.494863925 | 0.903652175 | 0.122305614 | 0.578229622 | 0.43972802 | -0.453326396 |
| Com_5474_pos | -0.089675054 | 0.670630342 | 0.753897713 | 0.457615848 | 0.887730128 | 0.077174295 | 0.576155291 | 0.454607216 | -0.417738576 |
| Com_12496_pos | 0.044027561 | 0.570955988 | 0.817168454 | 0.531651561 | 0.882390637 | 0.279254148 | 0.261378299 | 0.216336869 | -0.352213249 |
| Com_1089_neg | -0.094243751 | 0.483742665 | 0.565863869 | 0.640150776 | 0.745067053 | -0.042213584 | 0.121326108 | 0.235468995 | -0.379923268 |
| Com_20131_pos | 0.377459004 | -0.617811322 | -0.563539819 | -0.454872674 | -0.512346984 | -0.258827851 | -0.345700626 | -0.19919353 | 0.472984671 |
| Com_874_neg | 0.452982155 | -0.621732426 | -0.424167483 | -0.384989782 | -0.552457219 | -0.217173776 | -0.520952087 | -0.405001575 | 0.347296133 |
| Com_337_neg | 0.334526092 | -0.747103069 | -0.628342076 | -0.632840116 | -0.601195095 | -0.258835099 | -0.507433992 | -0.387649103 | 0.702441981 |
| Com_6829_pos | -0.473437927 | 0.642066997 | 0.370730194 | 0.436861048 | 0.401591407 | 0.245523608 | 0.627314301 | 0.14826643 | -0.498370039 |
| Com_1268_neg | 0.067285807 | 0.504956862 | 0.685440197 | 0.487208505 | 0.900578586 | -0.020492677 | 0.18293374 | 0.239320954 | -0.303728714 |
| Com_816_neg | 0.545743555 | -0.579170387 | -0.279132753 | -0.262196704 | -0.252557068 | -0.356337776 | -0.575029134 | -0.552670242 | 0.385870437 |
| Com_4404_neg | -0.44431183 | 0.548087179 | 0.616263623 | 0.646428187 | 0.503556018 | 0.28324983 | 0.441239499 | 0.312033863 | -0.564571893 |
| Com_777_neg | -0.001439343 | 0.576814143 | 0.758763227 | 0.479783408 | 0.816550879 | 0.155268746 | 0.364929996 | 0.258324263 | -0.439738972 |
| Com_2277_pos | -0.507622012 | 0.473795333 | 0.241621638 | 0.282943275 | 0.350997529 | 0.105861816 | 0.144529291 | 0.182004956 | -0.369125476 |
| Com_101_neg | -0.225146162 | 0.517803554 | 0.590195633 | 0.494858473 | 0.76604604 | -0.038025315 | 0.281741202 | 0.462490043 | -0.506733807 |
| Com_1024_neg | -0.448374887 | 0.648722067 | 0.567334536 | 0.737016584 | 0.580313775 | 0.164360221 | 0.461434093 | 0.375171697 | -0.711419696 |

Continued

| ID | Prot_A0A0H2UHM3 | Prot_A0A0H2UHT3 | Prot_A0A0H2UHV6 | Prot_A0A0H2UI34 | Prot_A0A140TAC4 | Prot_A0A140TAI2 | Prot_A0A1K0FUA6 | Prot_A0A1W2Q680 | Prot_A0A5D0 |
| --- | --- | --- | --- | --- | --- | --- | --- | --- | --- |
| Com_4739_neg | 0.389116378 | 0.346963675 | 0.530119674 | -0.494251655 | -0.506456957 | 0.136737202 | 0.458433125 | 0.352695042 | -0.415808331 |
| Com_508_neg | -0.552205921 | -0.55377439 | -0.721260194 | 0.427999985 | 0.521728468 | -0.276735803 | -0.596234353 | -0.500615388 | 0.573496529 |
| Com_3647_pos | 0.467294415 | 0.607902636 | 0.557805247 | -0.37479007 | -0.632559031 | 0.43978807 | 0.597948868 | 0.574880379 | -0.369370275 |
| Com_4991_pos | -0.416017907 | -0.43259645 | -0.625365504 | 0.380841817 | 0.700504179 | -0.377849697 | -0.468323835 | -0.563021921 | 0.294410998 |
| Com_2609_pos | -0.578124265 | -0.670904325 | -0.649230505 | 0.503545987 | 0.42717379 | -0.4654399 | -0.65906713 | -0.590576326 | 0.508894028 |
| Com_586_pos | -0.466331804 | -0.495843904 | -0.627900839 | 0.517711364 | 0.566668084 | -0.338864993 | -0.55234897 | -0.543388859 | 0.357676029 |
| Com_165_neg | 0.736327125 | 0.485184439 | 0.459223988 | -0.608386409 | -0.352451213 | 0.424503854 | 0.450822176 | 0.591188891 | -0.695851887 |
| Com_6688_neg | 0.507070856 | 0.383839142 | 0.435854184 | -0.4577633 | -0.537399691 | 0.482922458 | 0.59493038 | 0.423614375 | -0.445757041 |
| Com_3685_neg | -0.45889381 | -0.467155167 | -0.687307246 | 0.556985173 | 0.588252431 | -0.288591876 | -0.565067433 | -0.559287401 | 0.405586664 |
| Com_313_pos | 0.462775648 | 0.290416641 | 0.312171839 | -0.414415428 | -0.670492274 | 0.430406693 | 0.471214065 | 0.438391139 | -0.344575572 |
| Com_15841_pos | -0.464026028 | -0.546615717 | -0.716466983 | 0.517311594 | 0.379210187 | -0.293832756 | -0.614705074 | -0.491751517 | 0.508883266 |
| Com_3856_pos | -0.47753903 | -0.576221472 | -0.690495321 | 0.505442398 | 0.408453446 | -0.361341934 | -0.671421466 | -0.522480705 | 0.459564551 |
| Com_1082_neg | 0.659757867 | 0.491870356 | 0.562063941 | -0.606026788 | -0.471669915 | 0.373092157 | 0.494287872 | 0.615309628 | -0.641898017 |
| Com_646_neg | 0.2757142 | 0.385861631 | 0.516073078 | -0.390180654 | -0.27149953 | 0.330516983 | 0.218057241 | 0.38546219 | -0.234579764 |
| Com_14903_pos | 0.9017794 | 0.464621727 | 0.173335705 | -0.429523056 | -0.277872716 | 0.316870106 | 0.382127153 | 0.525148329 | -0.875926788 |
| Com_2370_pos | -0.225827893 | -0.423432517 | -0.749347856 | 0.526440067 | 0.445733846 | -0.319122773 | -0.364749726 | -0.481138774 | 0.120455012 |
| Com_4083_neg | -0.398072157 | -0.228425808 | -0.417508522 | 0.46629037 | 0.679606604 | -0.182077839 | -0.211933374 | -0.502502783 | 0.278336371 |
| Com_3695_pos | 0.232611637 | 0.480431683 | 0.712849798 | -0.442822658 | -0.295095894 | 0.287429045 | 0.81530427 | 0.22403572 | -0.256689708 |
| Com_12307_pos | -0.533683304 | -0.562328156 | -0.476738657 | 0.491696884 | 0.546447117 | -0.515566609 | -0.525333807 | -0.608438356 | 0.389377335 |
| Com_8577_pos | 0.697444114 | 0.503763933 | 0.445008175 | -0.697592701 | -0.387166793 | 0.572777829 | 0.482917352 | 0.516563955 | -0.549071768 |
| Com_24285_pos | 0.338369737 | 0.428720619 | 0.682857681 | -0.380963822 | -0.660732387 | 0.497641991 | 0.694553533 | 0.497683769 | -0.212026425 |
| Com_6860_pos | -0.386143046 | -0.401754728 | -0.597049944 | 0.49598967 | 0.290450551 | -0.104466488 | -0.410377859 | -0.443583295 | 0.317399482 |
| Com_2781_pos | -0.298544078 | -0.480708504 | -0.544270832 | 0.263944263 | 0.462023452 | -0.079732428 | -0.16414325 | -0.355070677 | 0.4430014 |
| Com_6127_pos | -0.375140946 | -0.395260395 | -0.565160592 | 0.17043557 | 0.52817387 | -0.106545817 | -0.285387652 | -0.260865795 | 0.37749177 |
| Com_842_neg | 0.725135877 | 0.597449797 | 0.454620581 | -0.461153365 | -0.270544155 | 0.328009634 | 0.740897178 | 0.378157115 | -0.73794846 |
| Com_5298_pos | 0.658168245 | 0.520077375 | 0.496673256 | -0.484754716 | -0.480925809 | 0.273291514 | 0.419926788 | 0.537787412 | -0.620774807 |
| Com_1241_neg | -0.378457598 | -0.439845557 | -0.561293815 | 0.35155488 | 0.257294858 | -0.192726579 | -0.635521266 | -0.345714979 | 0.428851554 |
| Com_124_pos | 0.594693626 | 0.555477053 | 0.490039198 | -0.425255049 | -0.365658463 | 0.671395101 | 0.632485161 | 0.542852425 | -0.439295056 |
| Com_20491_pos | 0.383153349 | 0.674891115 | 0.560134649 | -0.248175308 | -0.508490083 | 0.388374553 | 0.440783328 | 0.465579381 | -0.350908519 |
| Com_941_neg | -0.371629267 | -0.407485959 | -0.631171745 | 0.613055177 | 0.590979822 | -0.278336482 | -0.686445098 | -0.48471262 | 0.278481855 |
| Com_8285_pos | 0.459174229 | 0.571598538 | 0.753727935 | -0.547072895 | -0.356567256 | 0.527309978 | 0.649412646 | 0.495816877 | -0.337488716 |
| Com_2128_neg | 0.782009825 | 0.297502711 | 0.284740336 | -0.490576884 | -0.531495985 | 0.285408743 | 0.323706927 | 0.774789236 | -0.538451086 |
| Com_4694_pos | -0.492279934 | -0.534668923 | -0.606142415 | 0.263167973 | 0.271735701 | -0.260686718 | -0.402011237 | -0.382037279 | 0.493588577 |
| Com_1660_neg | -0.449047381 | -0.511856763 | -0.561781904 | 0.447281148 | 0.37905122 | -0.469277226 | -0.663435305 | -0.55187746 | 0.297771626 |
| Com_11428_pos | -0.578447599 | -0.383105513 | -0.39304138 | 0.449597772 | 0.309426771 | -0.413683423 | -0.57328099 | -0.430119152 | 0.503134297 |
| Com_3979_pos | 0.877971995 | 0.659253069 | 0.359903667 | -0.446016176 | -0.338308881 | 0.400615436 | 0.342511319 | 0.753348749 | -0.806561235 |
| Com_5474_pos | 0.848339711 | 0.602376911 | 0.317337649 | -0.423745169 | -0.357363768 | 0.307495125 | 0.299990177 | 0.736003422 | -0.778158646 |
| Com_12496_pos | 0.864196982 | 0.496177608 | 0.238944392 | -0.423394511 | -0.28581721 | 0.505113925 | 0.544453091 | 0.529712147 | -0.784988495 |
| Com_1089_neg | 0.705503604 | 0.284498378 | 0.18176476 | -0.501461551 | -0.485888801 | 0.249843593 | 0.43179381 | 0.411199835 | -0.673550599 |
| Com_20131_pos | -0.55157587 | -0.306837749 | -0.466537091 | 0.449557714 | 0.396231824 | -0.129734724 | -0.683984184 | -0.327534412 | 0.495217835 |
| Com_874_neg | -0.649831207 | -0.511324194 | -0.407510281 | 0.548266956 | 0.484457774 | -0.711081695 | -0.462496098 | -0.811802386 | 0.309217567 |
| Com_337_neg | -0.60725331 | -0.634180485 | -0.592772933 | 0.381929303 | 0.358475208 | -0.43018786 | -0.60915144 | -0.539979505 | 0.61843135 |
| Com_6829_pos | 0.429189479 | 0.611354244 | 0.672327083 | -0.438287899 | -0.313796198 | 0.301414917 | 0.40432661 | 0.414907946 | -0.407776621 |
| Com_1268_neg | 0.862140019 | 0.355834836 | 0.10904388 | -0.472234138 | -0.366637998 | 0.327707879 | 0.409564461 | 0.537934203 | -0.76305747 |
| Com_816_neg | -0.348952052 | -0.439979234 | -0.506502813 | 0.314081271 | 0.458136448 | -0.681083588 | -0.450882909 | -0.7492657 | 0.041677884 |
| Com_4404_neg | 0.566110408 | 0.594243039 | 0.4772401 | -0.468723339 | -0.227836785 | 0.409730972 | 0.410101101 | 0.428564133 | -0.593009849 |
| Com_777_neg | 0.754394748 | 0.524914782 | 0.537436551 | -0.486867691 | -0.256942561 | 0.402244646 | 0.650235264 | 0.437806202 | -0.773116499 |
| Com_2277_pos | 0.466575217 | 0.134677323 | 0.386686343 | -0.530869968 | -0.534567862 | 0.356913503 | 0.54223619 | 0.415438617 | -0.214940674 |
| Com_101_neg | 0.82620969 | 0.425095215 | 0.238673616 | -0.555170943 | -0.247182405 | 0.496180293 | 0.425878681 | 0.66810104 | -0.636812087 |
| Com_1024_neg | 0.637449232 | 0.527815672 | 0.515786888 | -0.490460909 | -0.430525561 | 0.355697526 | 0.601899849 | 0.546837228 | -0.577923506 |

Continued

| ID | Prot_A0A5H1ZRU5 | Prot_A1A5S1 | Prot_A1L1K8 | Prot_A2IBE0 | Prot_A9CMB7 | Prot_A9UK05 | Prot_B0BMT8 | Prot_B0BMX3 | Prot_B0BN35 | Prot_B0BN59 |
| --- | --- | --- | --- | --- | --- | --- | --- | --- | --- | --- |
| Com_4739_neg | 0.574948989 | 0.626119795 | -0.655404138 | 0.756507752 | -0.574633107 | 0.345283721 | -0.549311084 | -0.720473002 | -0.502435888 | -0.639757862 |
| Com_508_neg | -0.7379755 | -0.813551589 | 0.750181214 | -0.632863681 | 0.472590822 | -0.392588155 | 0.755917982 | 0.637485482 | 0.491576495 | 0.629459628 |
| Com_3647_pos | 0.501501408 | 0.742286987 | -0.690334031 | 0.548732433 | -0.496055679 | 0.410901065 | -0.619494709 | -0.75115981 | -0.420798582 | -0.646021472 |
| Com_4991_pos | -0.575643541 | -0.717066461 | 0.751522103 | -0.633267299 | 0.519000376 | -0.313421154 | 0.571596737 | 0.737609722 | 0.404181894 | 0.662953627 |
| Com_2609_pos | -0.719466163 | -0.799230831 | 0.767442719 | -0.597415882 | 0.512835195 | -0.574887847 | 0.739371311 | 0.648317414 | 0.580398348 | 0.668206286 |
| Com_586_pos | -0.66821904 | -0.695177309 | 0.779114624 | -0.648302877 | 0.596697141 | -0.387765576 | 0.634201175 | 0.70035328 | 0.54636418 | 0.699691337 |
| Com_165_neg | 0.742201702 | 0.773826637 | -0.714365626 | 0.575522007 | -0.44305189 | 0.598375975 | -0.704632017 | -0.463615203 | -0.625315419 | -0.691427777 |
| Com_6688_neg | 0.505716586 | 0.718828951 | -0.555943336 | 0.494081725 | -0.342996065 | 0.508669562 | -0.558461792 | -0.490269273 | -0.470795501 | -0.524715456 |
| Com_3685_neg | -0.715727563 | -0.737117407 | 0.716139394 | -0.598913852 | 0.716147753 | -0.321226125 | 0.708478478 | 0.678510034 | 0.651301881 | 0.803905559 |
| Com_313_pos | 0.345285203 | 0.683652826 | -0.449852915 | 0.361014052 | -0.354728309 | 0.433186493 | -0.422118342 | -0.485637828 | -0.368691467 | -0.537373425 |
| Com_15841_pos | -0.78044186 | -0.697135629 | 0.729799294 | -0.586049646 | 0.614925587 | -0.406235761 | 0.737517345 | 0.589438551 | 0.575120279 | 0.715049426 |
| Com_3856_pos | -0.748805171 | -0.728085139 | 0.721658936 | -0.604599079 | 0.650134132 | -0.446321804 | 0.736708781 | 0.678217677 | 0.638725015 | 0.756863122 |
| Com_1082_neg | 0.728088351 | 0.789080555 | -0.776778214 | 0.615715063 | -0.513749984 | 0.486414412 | -0.715463524 | -0.550269105 | -0.580962688 | -0.718815494 |
| Com_646_neg | 0.690599344 | 0.407762308 | -0.619579838 | 0.69291735 | -0.687255122 | 0.411904273 | -0.555086253 | -0.628484073 | -0.644649371 | -0.750578964 |
| Com_14903_pos | 0.442188138 | 0.835870901 | -0.465984722 | 0.234795694 | 0.05960495 | 0.618019251 | -0.538007239 | -0.162257152 | -0.268975847 | -0.290619989 |
| Com_2370_pos | -0.641140122 | -0.523804593 | 0.800824057 | -0.71785771 | 0.688709425 | -0.281683503 | 0.593906892 | 0.699401704 | 0.543246091 | 0.693437324 |
| Com_4083_neg | -0.553914305 | -0.558405508 | 0.567245556 | -0.538143034 | 0.662079137 | -0.193086037 | 0.480858894 | 0.603477377 | 0.543227596 | 0.763222741 |
| Com_3695_pos | 0.23032458 | 0.53635974 | -0.675392307 | 0.440415153 | -0.232230156 | 0.129285131 | -0.615256036 | -0.509030857 | -0.25139831 | -0.254325319 |
| Com_12307_pos | -0.552318512 | -0.741262175 | 0.739790774 | -0.624209867 | 0.472008895 | -0.586574463 | 0.55742017 | 0.711244868 | 0.451052619 | 0.649972319 |
| Com_8577_pos | 0.483166777 | 0.765154044 | -0.654058977 | 0.436682982 | -0.319654455 | 0.62092691 | -0.707325009 | -0.393598919 | -0.58016702 | -0.564786426 |
| Com_24285_pos | 0.541407277 | 0.725616298 | -0.637776156 | 0.462385001 | -0.51117686 | 0.328372213 | -0.603291635 | -0.618369574 | -0.488613889 | -0.614085732 |
| Com_6860_pos | -0.634681592 | -0.54455215 | 0.700656321 | -0.562617524 | 0.620946001 | -0.287517458 | 0.557902279 | 0.580957597 | 0.565159402 | 0.648480529 |
| Com_2781_pos | -0.570267109 | -0.534598542 | 0.495207774 | -0.732373398 | 0.420118859 | -0.120006382 | 0.605496559 | 0.721249058 | 0.199556464 | 0.543459014 |
| Com_6127_pos | -0.38940872 | -0.652815417 | 0.56112796 | -0.626146971 | 0.058023153 | -0.198168158 | 0.503610913 | 0.582527097 | 0.046176374 | 0.206810887 |
| Com_842_neg | 0.430908454 | 0.821864753 | -0.614282572 | 0.389922587 | -0.139290477 | 0.584892949 | -0.69281964 | -0.401711633 | -0.43550353 | -0.353699166 |
| Com_5298_pos | 0.566308979 | 0.758338663 | -0.772297529 | 0.595322508 | -0.356317086 | 0.46167614 | -0.631583387 | -0.590986858 | -0.379959013 | -0.574492798 |
| Com_1241_neg | -0.684707277 | -0.562855669 | 0.495669761 | -0.352561757 | 0.513457476 | -0.344365207 | 0.597134161 | 0.386223326 | 0.565449347 | 0.557446653 |
| Com_124_pos | 0.557178364 | 0.717844955 | -0.615704929 | 0.335495102 | -0.301973594 | 0.570432803 | -0.668115688 | -0.401897794 | -0.533031853 | -0.542611036 |
| Com_20491_pos | 0.463546382 | 0.715956359 | -0.545016646 | 0.628778039 | -0.312943286 | 0.414798703 | -0.606372269 | -0.76609992 | -0.24014422 | -0.461137463 |
| Com_941_neg | -0.542251487 | -0.638926069 | 0.703480684 | -0.546264641 | 0.661109846 | -0.199867755 | 0.621578744 | 0.673271945 | 0.56428283 | 0.703968088 |
| Com_8285_pos | 0.533581194 | 0.726503875 | -0.788471575 | 0.461132336 | -0.501918265 | 0.511631955 | -0.743445007 | -0.528540216 | -0.622738228 | -0.612475344 |
| Com_2128_neg | 0.469253761 | 0.808457156 | -0.675693859 | 0.203615558 | -0.357080905 | 0.438266311 | -0.403625277 | -0.354344425 | -0.40649786 | -0.628101863 |
| Com_4694_pos | -0.520036318 | -0.674195175 | 0.63615407 | -0.481927292 | 0.223743977 | -0.392778944 | 0.622138813 | 0.524092677 | 0.23987474 | 0.423612275 |
| Com_1660_neg | -0.660887843 | -0.648172637 | 0.670226506 | -0.431790509 | 0.611691014 | -0.503589019 | 0.589840028 | 0.568827683 | 0.650252499 | 0.711609249 |
| Com_11428_pos | -0.435845994 | -0.748528381 | 0.551255499 | -0.46696441 | 0.347896089 | -0.575413283 | 0.517252761 | 0.577914551 | 0.427982991 | 0.587636478 |
| Com_3979_pos | 0.556896632 | 0.902181105 | -0.587510922 | 0.258576322 | -0.109374218 | 0.575434803 | -0.655416699 | -0.293874938 | -0.299842393 | -0.446822927 |
| Com_5474_pos | 0.485939121 | 0.89570636 | -0.513896673 | 0.232558731 | -0.083086371 | 0.484447593 | -0.587472511 | -0.325151236 | -0.19298332 | -0.422309228 |
| Com_12496_pos | 0.423213722 | 0.823061413 | -0.509232923 | 0.148310666 | 0.057279378 | 0.647336526 | -0.577163697 | -0.122104646 | -0.32440779 | -0.29393897 |
| Com_1089_neg | 0.356225957 | 0.768708879 | -0.406145353 | 0.329077663 | -0.155345405 | 0.495339092 | -0.454068917 | -0.317355167 | -0.339400774 | -0.406334155 |
| Com_20131_pos | -0.314478616 | -0.754494286 | 0.504582644 | -0.315217764 | 0.239801017 | -0.246112895 | 0.512341689 | 0.510755091 | 0.254987265 | 0.431451247 |
| Com_874_neg | -0.482235036 | -0.772842499 | 0.736060233 | -0.293674831 | 0.427531053 | -0.638169928 | 0.510923265 | 0.465170264 | 0.531560667 | 0.671864958 |
| Com_337_neg | -0.792650733 | -0.768027023 | 0.687806262 | -0.488165097 | 0.391271895 | -0.610826581 | 0.712887281 | 0.440031474 | 0.543426278 | 0.565215899 |
| Com_6829_pos | 0.492337862 | 0.659284017 | -0.612537044 | 0.491099812 | -0.353855834 | 0.329610992 | -0.719113716 | -0.558266958 | -0.343390062 | -0.491316843 |
| Com_1268_neg | 0.326212552 | 0.831766993 | -0.409727376 | 0.123179198 | 0.013047534 | 0.592689188 | -0.436183255 | -0.139388874 | -0.279689709 | -0.314315647 |
| Com_816_neg | -0.547843237 | -0.571369563 | 0.694313455 | -0.302136057 | 0.517934527 | -0.424664189 | 0.406520848 | 0.518097721 | 0.437494221 | 0.670679779 |
| Com_4404_neg | 0.549691327 | 0.621925525 | -0.660866918 | 0.621782116 | -0.356499168 | 0.499899726 | -0.716936684 | -0.565086929 | -0.476450429 | -0.568625023 |
| Com_777_neg | 0.524852574 | 0.842683878 | -0.630690133 | 0.319681312 | -0.019564212 | 0.50529209 | -0.738777519 | -0.212975578 | -0.358353057 | -0.294907988 |
| Com_2277_pos | 0.301787272 | 0.594707132 | -0.699506088 | 0.287851501 | -0.420506366 | 0.420383322 | -0.344944948 | -0.375574963 | -0.507688266 | -0.532863132 |
| Com_101_neg | 0.583640802 | 0.799274641 | -0.614786762 | 0.34194907 | -0.355246738 | 0.779041545 | -0.507265485 | -0.362825674 | -0.608556878 | -0.650249073 |
| Com_1024_neg | 0.668393808 | 0.83506299 | -0.605396549 | 0.509430486 | -0.548088293 | 0.576474586 | -0.679708264 | -0.611442906 | -0.649069162 | -0.730978166 |

**Supplement Table 2** The Detailed List of DEMs and DEPs Correlations between B3 and B2

| ID | Prot_A0A023ILN9 | Prot_A0A096MJP9 | Prot_A0A096MK73 | Prot_A0A096MK77 | Prot_A0A096XKT2 | Prot_A0A096XNM4 | Prot_A0A0A0MY35 | Prot_A0A0G2JSM8 | Prot_A0A0G2JSS1 |
| --- | --- | --- | --- | --- | --- | --- | --- | --- | --- |
| Com_2013_pos | -0.50858 | 0.291251 | 0.673523 | -0.60346 | -0.46679 | -0.80223 | -0.67292 | -0.65136 | -0.34389306 |
| Com_23145_pos | -0.6273 | 0.336771 | 0.54692 | -0.59236 | -0.46041 | -0.69208 | -0.56674 | -0.555 | -0.42088101 |
| Com_3764_pos | -0.47785 | 0.247647 | 0.49123 | -0.47644 | -0.41075 | -0.75179 | -0.57667 | -0.69683 | -0.51201344 |
| Com_692_pos | 0.359929 | -0.44948 | -0.42628 | 0.352663 | 0.63728 | 0.747406 | 0.249437 | 0.47051 | 0.610917854 |
| Com_9745_pos | 0.385548 | -0.44852 | -0.39044 | 0.233592 | 0.715152 | 0.755185 | 0.177255 | 0.288751 | 0.589919416 |
| Com_6269_pos | -0.50665 | 0.138484 | 0.634342 | -0.60877 | -0.4377 | -0.76881 | -0.61154 | -0.57846 | -0.33606086 |
| Com_165_neg | 0.656786 | -0.20962 | -0.67016 | 0.67569 | 0.459063 | 0.705636 | 0.614663 | 0.490419 | 0.395567382 |
| Com_1512_pos | 0.756916 | -0.23145 | -0.52156 | 0.547065 | 0.300235 | 0.562976 | 0.503757 | 0.446158 | 0.551152415 |
| Com_1241_neg | -0.49489 | 0.220755 | 0.573108 | -0.60056 | -0.46506 | -0.76969 | -0.71968 | -0.56358 | -0.32159506 |
| Com_780_pos | -0.53962 | 0.215341 | 0.716179 | -0.6567 | -0.32358 | -0.68414 | -0.61857 | -0.62007 | -0.32981484 |
| Com_9406_pos | -0.48088 | 0.179276 | 0.473513 | -0.5807 | -0.41564 | -0.80736 | -0.79983 | -0.66412 | -0.33514355 |
| Com_308_pos | -0.49722 | 0.188667 | 0.563625 | -0.63226 | -0.40717 | -0.76963 | -0.78766 | -0.59498 | -0.32635817 |
| Com_8285_pos | 0.420347 | -0.44224 | -0.42984 | 0.415038 | 0.233221 | 0.61072 | 0.553462 | 0.408609 | 0.211073169 |
| Com_1214_neg | -0.47725 | 0.23557 | 0.542198 | -0.59755 | -0.42165 | -0.76167 | -0.75402 | -0.5883 | -0.35034378 |
| Com_3075_neg | -0.42142 | 0.116814 | 0.549999 | -0.5948 | -0.41917 | -0.70101 | -0.69369 | -0.54225 | -0.28068039 |
| Com_619_pos | -0.66091 | 0.280843 | 0.66875 | -0.65887 | -0.31987 | -0.72793 | -0.62197 | -0.56455 | -0.41125356 |
| Com_1617_neg | -0.4794 | 0.357929 | 0.503265 | -0.57681 | -0.44397 | -0.68558 | -0.67391 | -0.43968 | -0.30781258 |
| Com_909_pos | -0.49458 | 0.18396 | 0.567889 | -0.63078 | -0.385 | -0.744 | -0.78328 | -0.60643 | -0.33009733 |
| Com_433_neg | -0.49441 | 0.250321 | 0.597325 | -0.58393 | -0.37931 | -0.72683 | -0.68874 | -0.5886 | -0.35313442 |
| Com_3856_pos | -0.48228 | 0.312111 | 0.56792 | -0.40998 | -0.53781 | -0.84528 | -0.62807 | -0.59284 | -0.38931002 |
| Com_101_neg | 0.656559 | -0.1982 | -0.67729 | 0.705886 | 0.304575 | 0.642052 | 0.690482 | 0.466893 | 0.299824146 |
| Com_508_neg | -0.59134 | 0.220212 | 0.571277 | -0.55756 | -0.41596 | -0.65975 | -0.37225 | -0.50242 | -0.38294318 |
| Com_260_pos | -0.56106 | 0.157 | 0.448912 | -0.63671 | -0.43817 | -0.78849 | -0.75643 | -0.66911 | -0.39565895 |
| Com_3273_neg | -0.50433 | 0.130646 | 0.570723 | -0.62545 | -0.40551 | -0.70238 | -0.74607 | -0.59488 | -0.32564101 |
| Com_1503_neg | -0.6474 | 0.114765 | 0.62949 | -0.71572 | -0.40197 | -0.68021 | -0.71439 | -0.54439 | -0.34360708 |
| Com_295_pos | -0.54066 | 0.251329 | 0.610544 | -0.63399 | -0.39742 | -0.76113 | -0.77047 | -0.5392 | -0.33107381 |
| Com_2609_pos | -0.50113 | 0.216346 | 0.584137 | -0.59875 | -0.3387 | -0.73004 | -0.74096 | -0.64667 | -0.36680565 |
| Com_2200_neg | 0.545971 | -0.01091 | -0.42928 | 0.463818 | 0.519884 | 0.716505 | 0.627953 | 0.40979 | 0.536465316 |
| Com_829_neg | -0.46724 | 0.190852 | 0.565916 | -0.61534 | -0.42604 | -0.75196 | -0.78403 | -0.54685 | -0.27787437 |
| Com_2636_neg | -0.35966 | 0.131192 | 0.488521 | -0.56836 | -0.41276 | -0.65504 | -0.65526 | -0.54098 | -0.28118768 |
| Com_383_pos | -0.67517 | 0.192324 | 0.641381 | -0.54577 | -0.44873 | -0.74269 | -0.4179 | -0.40564 | -0.43943666 |
| Com_1672_neg | -0.46021 | 0.042606 | 0.50945 | -0.56753 | -0.42676 | -0.70045 | -0.70815 | -0.48024 | -0.20731677 |
| Com_1082_neg | 0.702747 | -0.19744 | -0.6812 | 0.58457 | 0.392272 | 0.700119 | 0.441798 | 0.458306 | 0.403072902 |
| Com_262_pos | -0.56622 | 0.108268 | 0.49048 | -0.65009 | -0.38675 | -0.77753 | -0.8202 | -0.64832 | -0.31754073 |
| Com_620_pos | 0.358256 | -0.4275 | -0.46642 | 0.353041 | 0.366358 | 0.831249 | 0.47248 | 0.587082 | 0.512736412 |
| Com_683_neg | -0.36139 | 0.18796 | 0.536431 | -0.54945 | -0.4378 | -0.72344 | -0.73651 | -0.50842 | -0.27575695 |
| Com_4633_pos | -0.55334 | 0.135151 | 0.549764 | -0.67731 | -0.32076 | -0.75047 | -0.78645 | -0.66459 | -0.27217414 |
| Com_2633_pos | -0.49605 | 0.107295 | 0.67445 | -0.64945 | -0.40633 | -0.71655 | -0.73085 | -0.60193 | -0.3420304 |
| Com_339_neg | 0.429939 | -0.26723 | -0.44988 | 0.292863 | 0.125179 | 0.498881 | 0.143991 | 0.224375 | 0.040892981 |
| Com_11428_pos | -0.70766 | 0.210497 | 0.593771 | -0.50487 | -0.37336 | -0.6947 | -0.49475 | -0.44167 | -0.57009408 |
| Com_12307_pos | -0.55962 | 0.272793 | 0.486371 | -0.49335 | -0.32299 | -0.77055 | -0.60868 | -0.57002 | -0.53447805 |
| Com_1827_neg | -0.49879 | 0.115362 | 0.574188 | -0.65238 | -0.33647 | -0.68478 | -0.75854 | -0.52137 | -0.26954049 |
| Com_2532_neg | -0.53718 | 0.541021 | 0.456014 | -0.45916 | -0.49553 | -0.76557 | -0.64105 | -0.35849 | -0.24249309 |
| Com_1031_neg | -0.46575 | 0.133547 | 0.534219 | -0.6016 | -0.32121 | -0.65442 | -0.7922 | -0.6061 | -0.29046002 |
| Com_955_neg | -0.54324 | 0.487987 | 0.459171 | -0.34377 | -0.23118 | -0.60433 | -0.21402 | -0.23747 | -0.26243526 |
| Com_137_neg | -0.52791 | 0.133153 | 0.554212 | -0.65362 | -0.37688 | -0.70157 | -0.79599 | -0.57159 | -0.28896501 |
| Com_1660_neg | -0.32033 | 0.291896 | 0.560351 | -0.45827 | -0.44264 | -0.67121 | -0.58548 | -0.49256 | -0.31760814 |
| Com_1844_pos | -0.4358 | 0.150194 | 0.568381 | -0.59349 | -0.31967 | -0.68649 | -0.81345 | -0.54411 | -0.2782748 |
| Com_337_neg | -0.67983 | 0.079679 | 0.645622 | -0.70658 | -0.30504 | -0.71101 | -0.68464 | -0.53267 | -0.33103425 |
| Com_4071_pos | 0.321143 | -0.49901 | -0.24961 | -0.00779 | 0.218032 | 0.486201 | 0.085528 | 0.161567 | 0.30629126 |

Continued

| ID | Prot_A0A0G2JSU7 | Prot_A0A0G2JT26 | Prot_A0A0G2JTB5 | Prot_A0A0G2JTB6 | Prot_A0A0G2JTE4 | Prot_A0A0G2JTQ5 | Prot_A0A0G2JTV8 | Prot_A0A0G2JUC3 | Prot_A0A0G2JV16 |
| --- | --- | --- | --- | --- | --- | --- | --- | --- | --- |
| Com_2013_pos | -0.63471 | -0.72169 | 0.579319 | 0.36077 | 0.757398 | -0.46493 | -0.45022 | 0.099221 | -0.522626994 |
| Com_23145_pos | -0.4719 | -0.57733 | 0.722815 | 0.276648 | 0.622282 | -0.40628 | -0.43584 | -0.01378 | -0.583144518 |
| Com_3764_pos | -0.62825 | -0.68691 | 0.66192 | 0.233845 | 0.728061 | -0.35144 | -0.38939 | -0.01631 | -0.568835485 |
| Com_692_pos | 0.382909 | 0.332894 | -0.58007 | -0.2604 | -0.77813 | 0.186562 | 0.544683 | -0.06997 | 0.412343667 |
| Com_9745_pos | 0.43667 | 0.35023 | -0.61009 | -0.41814 | -0.79382 | 0.139678 | 0.484021 | 0.086043 | 0.454270714 |
| Com_6269_pos | -0.69266 | -0.73715 | 0.596522 | 0.212282 | 0.759812 | -0.42909 | -0.49046 | 0.042523 | -0.495844221 |
| Com_165_neg | 0.463382 | 0.616787 | -0.51925 | -0.21158 | -0.69091 | 0.437934 | 0.619851 | -0.15989 | 0.445113107 |
| Com_1512_pos | 0.556627 | 0.719693 | -0.40447 | -0.08214 | -0.54743 | 0.520081 | 0.564913 | -0.02282 | 0.385203207 |
| Com_1241_neg | -0.65351 | -0.7252 | 0.650204 | 0.28614 | 0.741178 | -0.37463 | -0.42922 | -0.05089 | -0.425217874 |
| Com_780_pos | -0.57392 | -0.69336 | 0.579219 | 0.171377 | 0.681888 | -0.53368 | -0.48668 | 0.193356 | -0.561755345 |
| Com_9406_pos | -0.62349 | -0.72648 | 0.588936 | 0.251006 | 0.775019 | -0.34209 | -0.41605 | -0.03647 | -0.446855091 |
| Com_308_pos | -0.62591 | -0.72833 | 0.636956 | 0.227541 | 0.745912 | -0.37501 | -0.45599 | 0.007774 | -0.414158425 |
| Com_8285_pos | 0.305612 | 0.356014 | -0.74354 | -0.30797 | -0.64979 | 0.242775 | 0.305249 | -0.05654 | 0.380787194 |
| Com_1214_neg | -0.62543 | -0.69827 | 0.661709 | 0.243796 | 0.729577 | -0.36817 | -0.43653 | -0.02193 | -0.427680013 |
| Com_3075_neg | -0.66088 | -0.71067 | 0.616274 | 0.17812 | 0.690699 | -0.35154 | -0.41715 | -0.04448 | -0.37110955 |
| Com_619_pos | -0.51184 | -0.61642 | 0.587221 | 0.20535 | 0.666622 | -0.50639 | -0.57799 | 0.204042 | -0.557191459 |
| Com_1617_neg | -0.4377 | -0.50125 | 0.746549 | 0.339151 | 0.582212 | -0.26477 | -0.40119 | 0.022173 | -0.319642173 |
| Com_909_pos | -0.61807 | -0.73084 | 0.633921 | 0.218726 | 0.714384 | -0.38065 | -0.43748 | 0.016297 | -0.414621194 |
| Com_433_neg | -0.61982 | -0.68087 | 0.631325 | 0.237417 | 0.696954 | -0.44539 | -0.4455 | 0.041774 | -0.513926493 |
| Com_3856_pos | -0.61979 | -0.71424 | 0.634227 | 0.606394 | 0.790414 | -0.29767 | -0.29342 | -0.05214 | -0.585515858 |
| Com_101_neg | 0.569479 | 0.715964 | -0.41435 | -0.07415 | -0.68225 | 0.585215 | 0.624092 | -0.12658 | 0.368491117 |
| Com_508_neg | -0.51598 | -0.53652 | 0.665717 | 0.161443 | 0.632743 | -0.41624 | -0.50427 | 0.104404 | -0.564287767 |
| Com_260_pos | -0.57802 | -0.6623 | 0.581962 | 0.20119 | 0.666719 | -0.3266 | -0.4865 | -0.01119 | -0.416542524 |
| Com_3273_neg | -0.61092 | -0.72945 | 0.654577 | 0.218189 | 0.663904 | -0.36387 | -0.41181 | -0.00697 | -0.432954827 |
| Com_1503_neg | -0.56488 | -0.69358 | 0.644556 | 0.18539 | 0.588437 | -0.41236 | -0.52395 | 0.069656 | -0.421889692 |
| Com_295_pos | -0.61393 | -0.70078 | 0.62307 | 0.256621 | 0.728045 | -0.44349 | -0.49865 | 0.046219 | -0.457167343 |
| Com_2609_pos | -0.62034 | -0.70477 | 0.582349 | 0.197656 | 0.697275 | -0.46013 | -0.4604 | 0.074957 | -0.510975704 |
| Com_2200_neg | 0.555857 | 0.634863 | -0.74698 | -0.27386 | -0.67479 | 0.092483 | 0.469955 | 0.117329 | 0.409350299 |
| Com_829_neg | -0.65935 | -0.73381 | 0.620779 | 0.263395 | 0.718917 | -0.37645 | -0.42096 | -0.05665 | -0.382866445 |
| Com_2636_neg | -0.61461 | -0.64743 | 0.623478 | 0.110766 | 0.664255 | -0.32177 | -0.38675 | -0.08567 | -0.360198994 |
| Com_383_pos | -0.64945 | -0.65715 | 0.5861 | 0.319001 | 0.641921 | -0.42878 | -0.54227 | 0.044155 | -0.550510448 |
| Com_1672_neg | -0.66431 | -0.71519 | 0.582045 | 0.2615 | 0.64986 | -0.30395 | -0.38138 | -0.0911 | -0.341608096 |
| Com_1082_neg | 0.552159 | 0.646284 | -0.51679 | -0.19762 | -0.74085 | 0.502302 | 0.602564 | -0.12145 | 0.570800756 |
| Com_262_pos | -0.61215 | -0.74697 | 0.553103 | 0.240357 | 0.668323 | -0.35597 | -0.4344 | -0.00887 | -0.411047801 |
| Com_620_pos | 0.694577 | 0.611284 | -0.41506 | -0.23962 | -0.84441 | 0.45622 | 0.505234 | -0.10809 | 0.493912335 |
| Com_683_neg | -0.62889 | -0.68707 | 0.64262 | 0.267164 | 0.724061 | -0.2985 | -0.36899 | -0.07046 | -0.352660985 |
| Com_4633_pos | -0.64768 | -0.72358 | 0.523591 | 0.147129 | 0.667599 | -0.48798 | -0.48597 | 0.057036 | -0.477296242 |
| Com_2633_pos | -0.65159 | -0.77115 | 0.575218 | 0.212644 | 0.690498 | -0.43515 | -0.47941 | 0.110471 | -0.448007869 |
| Com_339_neg | 0.153723 | 0.045437 | -0.63268 | -0.30384 | -0.49982 | 0.217389 | 0.304205 | -0.15312 | 0.623236725 |
| Com_11428_pos | -0.56672 | -0.63836 | 0.613024 | 0.241871 | 0.670566 | -0.36531 | -0.58809 | 0.070642 | -0.475911197 |
| Com_12307_pos | -0.70215 | -0.71898 | 0.459721 | 0.108037 | 0.786569 | -0.49814 | -0.57285 | 0.001195 | -0.506909586 |
| Com_1827_neg | -0.63508 | -0.71797 | 0.602256 | 0.133114 | 0.66092 | -0.41513 | -0.46775 | 0.015746 | -0.38368996 |
| Com_2532_neg | -0.43278 | -0.46146 | 0.624394 | 0.545366 | 0.679259 | -0.36517 | -0.35076 | -0.15298 | -0.512675456 |
| Com_1031_neg | -0.59785 | -0.72784 | 0.600881 | 0.205619 | 0.605985 | -0.36041 | -0.35191 | -0.02604 | -0.376903883 |
| Com_955_neg | -0.37389 | -0.30597 | 0.56405 | 0.204796 | 0.698147 | -0.45757 | -0.4657 | 0.064796 | -0.581932471 |
| Com_137_neg | -0.60271 | -0.70636 | 0.637666 | 0.210903 | 0.626912 | -0.37809 | -0.43597 | 0.001603 | -0.400026364 |
| Com_1660_neg | -0.60254 | -0.6765 | 0.593693 | 0.309935 | 0.713003 | -0.35566 | -0.29999 | -0.05341 | -0.429486972 |
| Com_1844_pos | -0.58563 | -0.73401 | 0.605567 | 0.220335 | 0.699599 | -0.34114 | -0.39071 | 0.040505 | -0.335724741 |
| Com_337_neg | -0.66069 | -0.75922 | 0.535653 | 0.0785 | 0.691055 | -0.52914 | -0.60289 | 0.098834 | -0.475295572 |
| Com_4071_pos | 0.265523 | 0.184485 | -0.52984 | -0.38067 | -0.64198 | 0.149052 | 0.196525 | 0.11738 | 0.443575476 |

Continued

| ID | Prot_A0A0G2JVE6 | Prot_A0A0G2JW11 | Prot_A0A0G2JW14 | Prot_A0A0G2JW18 | Prot_A0A0G2JWE3 | Prot_A0A0G2JWN8 | Prot_A0A0G2JWU3 | Prot_A0A0G2JXC9 | Prot_A0A0G2JXE5 |
| --- | --- | --- | --- | --- | --- | --- | --- | --- | --- |
| Com_2013_pos | -0.55104 | 0.408874 | -0.60271 | 0.047966 | 0.475385 | -0.66323 | 0.505157 | 0.570048 | 0.616986542 |
| Com_23145_pos | -0.69331 | 0.550973 | -0.59095 | 0.125804 | 0.507832 | -0.43527 | 0.554054 | 0.561021 | 0.519224194 |
| Com_3764_pos | -0.53519 | 0.538628 | -0.44021 | -0.10326 | 0.469843 | -0.4279 | 0.564007 | 0.473533 | 0.646127824 |
| Com_692_pos | 0.389231 | -0.43474 | 0.363368 | -0.08478 | -0.3685 | 0.403545 | -0.2078 | -0.40766 | -0.344454166 |
| Com_9745_pos | 0.400783 | -0.4482 | 0.406991 | -0.00777 | -0.54045 | 0.321873 | -0.40456 | -0.37902 | -0.446165687 |
| Com_6269_pos | -0.53842 | 0.418767 | -0.45966 | 0.039239 | 0.466087 | -0.55539 | 0.533499 | 0.472476 | 0.635333808 |
| Com_165_neg | 0.647443 | -0.37621 | 0.596926 | -0.3177 | -0.40068 | 0.567896 | -0.27403 | -0.54622 | -0.435279378 |
| Com_1512_pos | 0.550723 | -0.6058 | 0.490077 | -0.27949 | -0.23354 | 0.463285 | -0.35318 | -0.45622 | -0.406979927 |
| Com_1241_neg | -0.60567 | 0.377651 | -0.5377 | 0.084545 | 0.469504 | -0.50842 | 0.587238 | 0.510139 | 0.664470394 |
| Com_780_pos | -0.62319 | 0.515681 | -0.52317 | 0.024567 | 0.512606 | -0.61019 | 0.482728 | 0.548924 | 0.610088605 |
| Com_9406_pos | -0.54281 | 0.275667 | -0.49176 | 0.069781 | 0.476273 | -0.42083 | 0.519319 | 0.453723 | 0.604459569 |
| Com_308_pos | -0.61594 | 0.341282 | -0.53869 | 0.076086 | 0.476027 | -0.47164 | 0.569094 | 0.484699 | 0.66735464 |
| Com_8285_pos | 0.692808 | -0.38532 | 0.626352 | 0.059412 | -0.55219 | 0.357072 | -0.57639 | -0.6746 | -0.663732338 |
| Com_1214_neg | -0.62731 | 0.401231 | -0.53681 | 0.024409 | 0.497056 | -0.46616 | 0.618381 | 0.504725 | 0.678579328 |
| Com_3075_neg | -0.57563 | 0.362035 | -0.42846 | 0.064571 | 0.430411 | -0.502 | 0.559279 | 0.492252 | 0.647765894 |
| Com_619_pos | -0.65901 | 0.520372 | -0.65209 | 0.037114 | 0.46799 | -0.50222 | 0.516286 | 0.481486 | 0.576934747 |
| Com_1617_neg | -0.6325 | 0.336451 | -0.69789 | 0.10104 | 0.375255 | -0.44446 | 0.635625 | 0.537924 | 0.601127252 |
| Com_909_pos | -0.61813 | 0.359343 | -0.53909 | 0.066424 | 0.452584 | -0.4776 | 0.564979 | 0.487505 | 0.668393158 |
| Com_433_neg | -0.65211 | 0.509704 | -0.54381 | -0.05733 | 0.568598 | -0.52926 | 0.644014 | 0.561588 | 0.681522225 |
| Com_3856_pos | -0.50561 | 0.326893 | -0.67552 | -0.0895 | 0.573075 | -0.49445 | 0.616768 | 0.47719 | 0.733169891 |
| Com_101_neg | 0.676049 | -0.45945 | 0.515956 | -0.30298 | -0.42366 | 0.615837 | -0.3369 | -0.58785 | -0.484572857 |
| Com_508_neg | -0.55087 | 0.551409 | -0.46269 | 0.095236 | 0.433628 | -0.54698 | 0.484902 | 0.605904 | 0.459433574 |
| Com_260_pos | -0.52256 | 0.28623 | -0.52756 | 0.167934 | 0.280465 | -0.41371 | 0.456688 | 0.413115 | 0.468142214 |
| Com_3273_neg | -0.63732 | 0.380535 | -0.52512 | 0.092903 | 0.440735 | -0.5054 | 0.558832 | 0.542606 | 0.64891008 |
| Com_1503_neg | -0.6836 | 0.410284 | -0.62241 | 0.224773 | 0.326943 | -0.56215 | 0.508819 | 0.580906 | 0.555293982 |
| Com_295_pos | -0.67701 | 0.430028 | -0.61023 | 0.02855 | 0.569245 | -0.51326 | 0.644231 | 0.53836 | 0.684970127 |
| Com_2609_pos | -0.63917 | 0.487104 | -0.53078 | -0.05191 | 0.542414 | -0.53518 | 0.595724 | 0.561735 | 0.657193921 |
| Com_2200_neg | 0.658751 | -0.34488 | 0.544232 | -0.02191 | -0.46274 | 0.241114 | -0.61926 | -0.41563 | -0.714613029 |
| Com_829_neg | -0.62176 | 0.344283 | -0.53785 | 0.063579 | 0.476381 | -0.48565 | 0.615369 | 0.480092 | 0.690288505 |
| Com_2636_neg | -0.58132 | 0.38766 | -0.33371 | 0.057244 | 0.442031 | -0.4333 | 0.52352 | 0.469268 | 0.601915466 |
| Com_383_pos | -0.50793 | 0.517046 | -0.61194 | -0.00214 | 0.381091 | -0.45298 | 0.631388 | 0.317243 | 0.605775311 |
| Com_1672_neg | -0.53815 | 0.246821 | -0.48921 | 0.109766 | 0.389353 | -0.5004 | 0.583132 | 0.503834 | 0.625033884 |
| Com_1082_neg | 0.62169 | -0.52788 | 0.516693 | -0.17618 | -0.49309 | 0.549704 | -0.35489 | -0.54736 | -0.506842075 |
| Com_262_pos | -0.51034 | 0.220553 | -0.55871 | 0.15984 | 0.313068 | -0.41212 | 0.498566 | 0.36818 | 0.540345637 |
| Com_620_pos | 0.295187 | -0.52948 | 0.3873 | 0.303786 | -0.54432 | 0.453924 | -0.60193 | -0.30282 | -0.61283522 |
| Com_683_neg | -0.5927 | 0.315598 | -0.47848 | -0.00457 | 0.507484 | -0.42803 | 0.612611 | 0.430823 | 0.7280849 |
| Com_4633_pos | -0.57384 | 0.37681 | -0.50959 | 0.067529 | 0.43031 | -0.53114 | 0.554093 | 0.506729 | 0.54458891 |
| Com_2633_pos | -0.59702 | 0.394783 | -0.53287 | 0.063933 | 0.452779 | -0.62239 | 0.519155 | 0.544327 | 0.657347991 |
| Com_339_neg | 0.656391 | -0.41583 | 0.550366 | 0.293422 | -0.64729 | 0.235874 | -0.52174 | -0.54787 | -0.535184602 |
| Com_11428_pos | -0.66489 | 0.586985 | -0.63704 | 0.032983 | 0.375047 | -0.45846 | 0.522106 | 0.531299 | 0.652780641 |
| Com_12307_pos | -0.53146 | 0.600823 | -0.40307 | -0.11204 | 0.544212 | -0.36689 | 0.590139 | 0.346057 | 0.622297415 |
| Com_1827_neg | -0.63806 | 0.387364 | -0.4947 | 0.062907 | 0.467508 | -0.4756 | 0.609788 | 0.484705 | 0.660881548 |
| Com_2532_neg | -0.68379 | 0.388305 | -0.73423 | 0.011891 | 0.711348 | -0.31152 | 0.718678 | 0.458557 | 0.582629988 |
| Com_1031_neg | -0.63085 | 0.361935 | -0.52364 | 0.053345 | 0.381592 | -0.46932 | 0.554919 | 0.503694 | 0.664913894 |
| Com_955_neg | -0.59126 | 0.629586 | -0.45232 | -0.13438 | 0.749915 | -0.2988 | 0.593628 | 0.51172 | 0.518058943 |
| Com_137_neg | -0.6568 | 0.361393 | -0.57532 | 0.098236 | 0.42336 | -0.50671 | 0.605302 | 0.554878 | 0.633456236 |
| Com_1660_neg | -0.51578 | 0.414247 | -0.41944 | -0.06235 | 0.571739 | -0.43912 | 0.575431 | 0.386288 | 0.700221749 |
| Com_1844_pos | -0.6177 | 0.290135 | -0.54184 | 0.044312 | 0.464779 | -0.45995 | 0.553193 | 0.483331 | 0.731545893 |
| Com_337_neg | -0.64152 | 0.468271 | -0.51561 | 0.146598 | 0.435861 | -0.53464 | 0.522208 | 0.510002 | 0.58229372 |
| Com_4071_pos | 0.514198 | -0.52618 | 0.389626 | 0.289982 | -0.66046 | 0.153305 | -0.48646 | -0.51986 | -0.602375462 |

Continued

| ID | Prot_A0A0G2JY58 | Prot_A0A0G2JYF3 | Prot_A0A0G2JYU6 | Prot_A0A0G2JZ48 | Prot_A0A0G2JZZ4 | Prot_A0A0G2K0B6 | Prot_A0A0G2K0I1 | Prot_A0A0G2K0K6 | Prot_A0A0G2K0M5 |
| --- | --- | --- | --- | --- | --- | --- | --- | --- | --- |
| Com_2013_pos | -0.45635 | -0.66376 | -0.7296 | 0.454994 | -0.53208 | -0.60361 | -0.45535 | -0.83752 | -0.498264192 |
| Com_23145_pos | -0.41113 | -0.61915 | -0.65618 | 0.5714 | -0.53966 | -0.66052 | -0.51428 | -0.71124 | -0.453591357 |
| Com_3764_pos | -0.53512 | -0.47887 | -0.67799 | 0.457252 | -0.39488 | -0.68888 | -0.66736 | -0.78297 | -0.37937229 |
| Com_692_pos | 0.697575 | 0.369339 | 0.663216 | -0.44557 | 0.485407 | 0.473306 | 0.505513 | 0.568923 | 0.599747099 |
| Com_9745_pos | 0.579303 | 0.42709 | 0.717977 | -0.49376 | 0.420014 | 0.483849 | 0.428976 | 0.491067 | 0.555859581 |
| Com_6269_pos | -0.46469 | -0.50966 | -0.72433 | 0.360732 | -0.41656 | -0.55601 | -0.57467 | -0.75486 | -0.355805916 |
| Com_165_neg | 0.429894 | 0.613924 | 0.792305 | -0.42874 | 0.72405 | 0.384271 | 0.236883 | 0.658323 | 0.559372423 |
| Com_1512_pos | 0.45427 | 0.527339 | 0.711103 | -0.43368 | 0.636283 | 0.529317 | 0.217364 | 0.466614 | 0.622623407 |
| Com_1241_neg | -0.50843 | -0.55543 | -0.70676 | 0.391194 | -0.34251 | -0.56452 | -0.52788 | -0.77159 | -0.359381217 |
| Com_780_pos | -0.30613 | -0.60551 | -0.7321 | 0.47319 | -0.60796 | -0.5962 | -0.52346 | -0.81233 | -0.310797634 |
| Com_9406_pos | -0.55053 | -0.44238 | -0.70596 | 0.284945 | -0.35181 | -0.45048 | -0.53208 | -0.78771 | -0.354586761 |
| Com_308_pos | -0.4922 | -0.49961 | -0.73076 | 0.369772 | -0.36441 | -0.53007 | -0.5306 | -0.79111 | -0.305409588 |
| Com_8285_pos | 0.352286 | 0.312161 | 0.631692 | -0.25281 | 0.29213 | 0.724087 | 0.469177 | 0.715199 | 0.293330251 |
| Com_1214_neg | -0.47316 | -0.5125 | -0.71664 | 0.387023 | -0.34068 | -0.58045 | -0.56732 | -0.79164 | -0.298434717 |
| Com_3075_neg | -0.46148 | -0.48232 | -0.6586 | 0.295748 | -0.26713 | -0.50443 | -0.58021 | -0.73055 | -0.219396678 |
| Com_619_pos | -0.32585 | -0.55212 | -0.76967 | 0.506914 | -0.65243 | -0.65208 | -0.41369 | -0.75887 | -0.502012984 |
| Com_1617_neg | -0.44987 | -0.51132 | -0.58307 | 0.505635 | -0.27273 | -0.73422 | -0.43916 | -0.68391 | -0.404087812 |
| Com_909_pos | -0.47758 | -0.51585 | -0.70945 | 0.392426 | -0.36645 | -0.55305 | -0.52725 | -0.79277 | -0.29826155 |
| Com_433_neg | -0.32263 | -0.57128 | -0.74177 | 0.400987 | -0.44928 | -0.61635 | -0.55971 | -0.80778 | -0.276933075 |
| Com_3856_pos | -0.48231 | -0.69609 | -0.73337 | 0.577415 | -0.43424 | -0.65249 | -0.36417 | -0.82532 | -0.545779133 |
| Com_101_neg | 0.339343 | 0.562198 | 0.822757 | -0.23896 | 0.675015 | 0.336588 | 0.264186 | 0.653383 | 0.426399341 |
| Com_508_neg | -0.38818 | -0.43567 | -0.62709 | 0.365111 | -0.50815 | -0.6918 | -0.58276 | -0.60712 | -0.443638614 |
| Com_260_pos | -0.60496 | -0.42459 | -0.61624 | 0.313346 | -0.36626 | -0.50078 | -0.48753 | -0.70395 | -0.530455044 |
| Com_3273_neg | -0.44037 | -0.55809 | -0.67857 | 0.394674 | -0.37275 | -0.55853 | -0.51985 | -0.7636 | -0.27952362 |
| Com_1503_neg | -0.41213 | -0.58775 | -0.67614 | 0.415433 | -0.48691 | -0.60302 | -0.40022 | -0.69045 | -0.44876059 |
| Com_295_pos | -0.34607 | -0.56988 | -0.79239 | 0.399519 | -0.46222 | -0.56101 | -0.47319 | -0.79361 | -0.311417563 |
| Com_2609_pos | -0.32659 | -0.54748 | -0.7532 | 0.346792 | -0.48486 | -0.56843 | -0.53877 | -0.82229 | -0.285367871 |
| Com_2200_neg | 0.481789 | 0.436791 | 0.768955 | -0.45216 | 0.297594 | 0.552346 | 0.424851 | 0.601094 | 0.307905791 |
| Com_829_neg | -0.45622 | -0.54723 | -0.70747 | 0.357219 | -0.30422 | -0.51799 | -0.50824 | -0.78062 | -0.286484094 |
| Com_2636_neg | -0.4646 | -0.43559 | -0.6111 | 0.270804 | -0.22441 | -0.44703 | -0.65821 | -0.71231 | -0.125443559 |
| Com_383_pos | -0.38328 | -0.55291 | -0.69831 | 0.583411 | -0.47641 | -0.7427 | -0.39442 | -0.60273 | -0.612636696 |
| Com_1672_neg | -0.42888 | -0.47319 | -0.62521 | 0.209925 | -0.21746 | -0.4774 | -0.44546 | -0.66131 | -0.296180169 |
| Com_1082_neg | 0.376578 | 0.543587 | 0.828682 | -0.3629 | 0.725088 | 0.484314 | 0.331871 | 0.636304 | 0.55978762 |
| Com_262_pos | -0.55319 | -0.46335 | -0.62722 | 0.3526 | -0.35001 | -0.48508 | -0.42931 | -0.72534 | -0.465804033 |
| Com_620_pos | 0.453761 | 0.309748 | 0.765445 | -0.31342 | 0.394017 | 0.616117 | 0.618079 | 0.72637 | 0.429026652 |
| Com_683_neg | -0.45662 | -0.51167 | -0.68602 | 0.387222 | -0.22588 | -0.49892 | -0.55399 | -0.77599 | -0.168553682 |
| Com_4633_pos | -0.37458 | -0.46717 | -0.6755 | 0.216751 | -0.43224 | -0.49493 | -0.52476 | -0.76988 | -0.363374905 |
| Com_2633_pos | -0.37271 | -0.61765 | -0.74365 | 0.418428 | -0.48504 | -0.53303 | -0.46653 | -0.78674 | -0.289271863 |
| Com_339_neg | -0.06195 | 0.203064 | 0.541877 | -0.09341 | 0.401739 | 0.636035 | 0.391635 | 0.594402 | 0.30125832 |
| Com_11428_pos | -0.43294 | -0.50657 | -0.82647 | 0.448435 | -0.57181 | -0.72317 | -0.30935 | -0.61819 | -0.62050568 |
| Com_12307_pos | -0.42242 | -0.38141 | -0.84252 | 0.304129 | -0.48982 | -0.50767 | -0.55287 | -0.69943 | -0.386123924 |
| Com_1827_neg | -0.35865 | -0.47508 | -0.70377 | 0.31012 | -0.33785 | -0.51584 | -0.53261 | -0.7326 | -0.194877146 |
| Com_2532_neg | -0.272 | -0.64634 | -0.70417 | 0.491667 | -0.43009 | -0.51503 | -0.29847 | -0.71677 | -0.481200363 |
| Com_1031_neg | -0.41976 | -0.54642 | -0.63065 | 0.364379 | -0.31317 | -0.54942 | -0.47742 | -0.77136 | -0.257932387 |
| Com_955_neg | -0.14267 | -0.2478 | -0.73112 | 0.217215 | -0.50932 | -0.60502 | -0.49709 | -0.5475 | -0.333283121 |
| Com_137_neg | -0.38763 | -0.53698 | -0.67045 | 0.336519 | -0.35507 | -0.56295 | -0.48036 | -0.74833 | -0.297938444 |
| Com_1660_neg | -0.40863 | -0.59679 | -0.66284 | 0.549165 | -0.31114 | -0.51826 | -0.55464 | -0.75916 | -0.148007067 |
| Com_1844_pos | -0.42544 | -0.49555 | -0.72206 | 0.374212 | -0.32546 | -0.51397 | -0.45115 | -0.78127 | -0.1880076 |
| Com_337_neg | -0.35497 | -0.46604 | -0.78538 | 0.269023 | -0.54152 | -0.51446 | -0.45342 | -0.68579 | -0.388318231 |
| Com_4071_pos | 0.235461 | 0.199897 | 0.636178 | -0.13224 | 0.285362 | 0.573876 | 0.327611 | 0.477279 | 0.335756426 |

Continued

| ID | Prot_A0A0G2K0P0 | Prot_A0A0G2K1Q8 | Prot_A0A0G2K1S4 | Prot_A0A0G2K1W9 | Prot_A0A0G2K2I6 | Prot_A0A0G2K2Y2 | Prot_A0A0G2K3H0 | Prot_A0A0G2K3J4 | Prot_A0A0G2K3N7 |
| --- | --- | --- | --- | --- | --- | --- | --- | --- | --- |
| Com_2013_pos | -0.45223 | -0.82368 | -0.55319 | -0.67909 | -0.42912 | -0.61552 | -0.24163 | -0.26893 | -0.440239967 |
| Com_23145_pos | -0.25007 | -0.77138 | -0.52251 | -0.72833 | -0.49165 | -0.46385 | -0.15409 | -0.24953 | -0.461913832 |
| Com_3764_pos | -0.33642 | -0.72522 | -0.41733 | -0.72509 | -0.44865 | -0.65572 | -0.28471 | -0.36552 | -0.489548481 |
| Com_692_pos | 0.560913 | 0.669063 | 0.233233 | 0.574078 | 0.657908 | 0.487 | 0.425922 | 0.274011 | 0.46442874 |
| Com_9745_pos | 0.44779 | 0.609677 | 0.06984 | 0.606213 | 0.590299 | 0.562372 | 0.430892 | 0.464447 | 0.52598688 |
| Com_6269_pos | -0.30314 | -0.74957 | -0.45253 | -0.70019 | -0.37281 | -0.68811 | -0.34062 | -0.32718 | -0.397132948 |
| Com_165_neg | 0.277251 | 0.771182 | 0.538612 | 0.467295 | 0.50345 | 0.41164 | 0.099175 | 0.085136 | 0.352586536 |
| Com_1512_pos | 0.087165 | 0.738383 | 0.468123 | 0.466749 | 0.46684 | 0.469713 | 0.025169 | 0.250069 | 0.413394501 |
| Com_1241_neg | -0.30771 | -0.76105 | -0.47525 | -0.72021 | -0.44073 | -0.68571 | -0.30488 | -0.28169 | -0.438901137 |
| Com_780_pos | -0.30514 | -0.75883 | -0.60716 | -0.72897 | -0.41227 | -0.5333 | -0.12986 | -0.26701 | -0.363767124 |
| Com_9406_pos | -0.32063 | -0.69151 | -0.38974 | -0.53524 | -0.33093 | -0.61999 | -0.28289 | -0.20613 | -0.441259968 |
| Com_308_pos | -0.30206 | -0.75192 | -0.48421 | -0.65741 | -0.409 | -0.66225 | -0.28314 | -0.23317 | -0.397438537 |
| Com_8285_pos | 0.498716 | 0.639566 | 0.523652 | 0.704624 | 0.43161 | 0.606272 | 0.316406 | 0.467896 | 0.588349673 |
| Com_1214_neg | -0.3085 | -0.75437 | -0.48768 | -0.70595 | -0.42527 | -0.67157 | -0.26937 | -0.2858 | -0.412509831 |
| Com_3075_neg | -0.23863 | -0.70285 | -0.45058 | -0.70425 | -0.37948 | -0.69171 | -0.29281 | -0.26234 | -0.380594299 |
| Com_619_pos | -0.32961 | -0.81657 | -0.6371 | -0.61588 | -0.43817 | -0.49808 | -0.16511 | -0.27737 | -0.30566772 |
| Com_1617_neg | -0.42987 | -0.85438 | -0.55677 | -0.70478 | -0.47271 | -0.62513 | -0.37808 | -0.22685 | -0.371378864 |
| Com_909_pos | -0.29132 | -0.7554 | -0.51157 | -0.67029 | -0.41837 | -0.6501 | -0.25746 | -0.21915 | -0.385845558 |
| Com_433_neg | -0.28901 | -0.74828 | -0.53841 | -0.72368 | -0.38127 | -0.6251 | -0.149 | -0.35827 | -0.400359821 |
| Com_3856_pos | -0.45721 | -0.75009 | -0.37687 | -0.65954 | -0.44488 | -0.66407 | -0.31026 | -0.36366 | -0.529052128 |
| Com_101_neg | 0.166987 | 0.706239 | 0.586714 | 0.505396 | 0.415453 | 0.471836 | -0.01842 | 0.202864 | 0.374323583 |
| Com_508_neg | -0.32811 | -0.77627 | -0.44841 | -0.66896 | -0.31916 | -0.57608 | -0.33797 | -0.38576 | -0.481985956 |
| Com_260_pos | -0.31721 | -0.78385 | -0.46672 | -0.44762 | -0.34466 | -0.55085 | -0.32019 | -0.08707 | -0.332515301 |
| Com_3273_neg | -0.23506 | -0.7565 | -0.51299 | -0.67268 | -0.39905 | -0.63391 | -0.2142 | -0.20041 | -0.399243456 |
| Com_1503_neg | -0.22086 | -0.86276 | -0.6274 | -0.59488 | -0.40262 | -0.57532 | -0.19983 | -0.13642 | -0.330584184 |
| Com_295_pos | -0.28731 | -0.77466 | -0.53487 | -0.66498 | -0.38773 | -0.63082 | -0.17228 | -0.31265 | -0.377202375 |
| Com_2609_pos | -0.28561 | -0.73947 | -0.55016 | -0.63691 | -0.34341 | -0.59555 | -0.09715 | -0.30876 | -0.383078771 |
| Com_2200_neg | 0.121087 | 0.715684 | 0.287617 | 0.530828 | 0.454864 | 0.706737 | 0.322608 | 0.27239 | 0.36889768 |
| Com_829_neg | -0.2644 | -0.73333 | -0.49384 | -0.69887 | -0.41348 | -0.67864 | -0.25834 | -0.25836 | -0.378012822 |
| Com_2636_neg | -0.18989 | -0.62274 | -0.41409 | -0.73323 | -0.40722 | -0.63071 | -0.2505 | -0.2377 | -0.375610334 |
| Com_383_pos | -0.2609 | -0.82923 | -0.46577 | -0.67297 | -0.40495 | -0.65211 | -0.40323 | -0.42332 | -0.283072404 |
| Com_1672_neg | -0.21959 | -0.70478 | -0.40585 | -0.56423 | -0.25028 | -0.70617 | -0.32344 | -0.25442 | -0.384788004 |
| Com_1082_neg | 0.235074 | 0.702796 | 0.47352 | 0.563404 | 0.438563 | 0.505612 | 0.154392 | 0.35668 | 0.463305849 |
| Com_262_pos | -0.2751 | -0.76199 | -0.46008 | -0.45929 | -0.30137 | -0.57515 | -0.31289 | -0.0825 | -0.318885682 |
| Com_620_pos | 0.539858 | 0.67069 | 0.270721 | 0.602608 | 0.30361 | 0.714519 | 0.38368 | 0.611181 | 0.40467105 |
| Com_683_neg | -0.27232 | -0.66988 | -0.4247 | -0.75221 | -0.45446 | -0.69554 | -0.29758 | -0.27155 | -0.368485663 |
| Com_4633_pos | -0.26045 | -0.74696 | -0.54334 | -0.5122 | -0.20711 | -0.56708 | -0.17452 | -0.21913 | -0.329651006 |
| Com_2633_pos | -0.2749 | -0.79319 | -0.54229 | -0.64578 | -0.38139 | -0.63762 | -0.17383 | -0.20441 | -0.338821283 |
| Com_339_neg | 0.371697 | 0.408301 | 0.505626 | 0.545275 | 0.191912 | 0.35969 | 0.237147 | 0.60801 | 0.368964042 |
| Com_11428_pos | -0.26673 | -0.82148 | -0.53525 | -0.58083 | -0.52867 | -0.66527 | -0.22995 | -0.4282 | -0.424759053 |
| Com_12307_pos | -0.21308 | -0.64498 | -0.36357 | -0.57527 | -0.36974 | -0.6294 | -0.15517 | -0.48676 | -0.384594225 |
| Com_1827_neg | -0.18659 | -0.72124 | -0.51239 | -0.66167 | -0.33218 | -0.65168 | -0.21531 | -0.26144 | -0.323364329 |
| Com_2532_neg | -0.31479 | -0.63457 | -0.42605 | -0.64345 | -0.43166 | -0.42345 | -0.10584 | -0.38179 | -0.43683323 |
| Com_1031_neg | -0.21357 | -0.71437 | -0.56307 | -0.65427 | -0.40598 | -0.6096 | -0.15177 | -0.17186 | -0.355141892 |
| Com_955_neg | -0.32367 | -0.48629 | -0.32113 | -0.67596 | -0.28309 | -0.48672 | -0.23168 | -0.75297 | -0.554625501 |
| Com_137_neg | -0.23269 | -0.78249 | -0.55621 | -0.61261 | -0.33401 | -0.62632 | -0.19569 | -0.19725 | -0.352061601 |
| Com_1660_neg | -0.27693 | -0.60815 | -0.35626 | -0.84954 | -0.51184 | -0.62166 | -0.22813 | -0.32714 | -0.414447434 |
| Com_1844_pos | -0.27825 | -0.70408 | -0.49483 | -0.65328 | -0.4174 | -0.67207 | -0.23134 | -0.21575 | -0.378759095 |
| Com_337_neg | -0.16122 | -0.76545 | -0.54434 | -0.55698 | -0.29156 | -0.61274 | -0.18874 | -0.29889 | -0.344855358 |
| Com_4071_pos | 0.370814 | 0.286561 | 0.176741 | 0.608552 | 0.425819 | 0.524168 | 0.198916 | 0.805372 | 0.694678251 |

Continued

| ID | Prot_A0A0G2K4L6 | Prot_A0A0G2K4M7 | Prot_A0A0G2K4R7 | Prot_A0A0G2K5D5 | Prot_A0A0G2K652 | Prot_A0A0G2K6N2 | Prot_A0A0G2K6Y2 | Prot_A0A0G2K716 | Prot_A0A0G2K782 |
| --- | --- | --- | --- | --- | --- | --- | --- | --- | --- |
| Com_2013_pos | -0.47172 | 0.730324 | 0.400094 | -0.59893 | -0.61573 | -0.41481 | -0.59192 | -0.37463 | -0.589942408 |
| Com_23145_pos | -0.51646 | 0.631147 | 0.47109 | -0.6483 | -0.64408 | -0.41413 | -0.51318 | -0.44763 | -0.557624878 |
| Com_3764_pos | -0.42889 | 0.696395 | 0.368765 | -0.67305 | -0.65007 | -0.45239 | -0.43702 | -0.51778 | -0.446626725 |
| Com_692_pos | 0.096883 | -0.63412 | -0.38996 | 0.320365 | 0.736312 | 0.495155 | 0.332485 | 0.232275 | 0.182974223 |
| Com_9745_pos | 0.144852 | -0.58444 | -0.42241 | 0.32405 | 0.680411 | 0.528275 | 0.292911 | 0.327654 | 0.09465906 |
| Com_6269_pos | -0.44939 | 0.79465 | 0.382324 | -0.61998 | -0.59642 | -0.53495 | -0.5171 | -0.40593 | -0.527119588 |
| Com_165_neg | 0.374486 | -0.57005 | -0.47545 | 0.438246 | 0.605479 | 0.464328 | 0.530478 | 0.100667 | 0.542022597 |
| Com_1512_pos | 0.309273 | -0.44686 | -0.30519 | 0.401116 | 0.631014 | 0.438011 | 0.465454 | 0.233624 | 0.422793789 |
| Com_1241_neg | -0.45559 | 0.750669 | 0.397809 | -0.65971 | -0.59583 | -0.45655 | -0.56446 | -0.47497 | -0.519799083 |
| Com_780_pos | -0.55107 | 0.69884 | 0.401667 | -0.65916 | -0.50216 | -0.36129 | -0.52545 | -0.41908 | -0.637600803 |
| Com_9406_pos | -0.36911 | 0.698419 | 0.403709 | -0.57638 | -0.59819 | -0.54778 | -0.53602 | -0.38196 | -0.479226365 |
| Com_308_pos | -0.45951 | 0.706824 | 0.404715 | -0.68001 | -0.52691 | -0.45175 | -0.57457 | -0.47113 | -0.542437921 |
| Com_8285_pos | 0.575404 | -0.4619 | -0.15386 | 0.718022 | 0.434812 | 0.340023 | 0.560598 | 0.710376 | 0.319568681 |
| Com_1214_neg | -0.48616 | 0.687818 | 0.36875 | -0.72071 | -0.52333 | -0.42712 | -0.58006 | -0.54528 | -0.528421488 |
| Com_3075_neg | -0.43612 | 0.750496 | 0.307278 | -0.67742 | -0.5428 | -0.44118 | -0.52962 | -0.4676 | -0.492010025 |
| Com_619_pos | -0.59629 | 0.533922 | 0.446167 | -0.66992 | -0.43621 | -0.41773 | -0.59643 | -0.43831 | -0.657423071 |
| Com_1617_neg | -0.53446 | 0.547344 | 0.323274 | -0.77592 | -0.43541 | -0.27011 | -0.66739 | -0.59576 | -0.5435622 |
| Com_909_pos | -0.47443 | 0.693227 | 0.398766 | -0.69685 | -0.51625 | -0.40713 | -0.56764 | -0.47688 | -0.559830877 |
| Com_433_neg | -0.57336 | 0.628284 | 0.315926 | -0.75163 | -0.47067 | -0.39242 | -0.58412 | -0.57365 | -0.566329119 |
| Com_3856_pos | -0.45774 | 0.658161 | 0.539176 | -0.57082 | -0.60897 | -0.3791 | -0.47269 | -0.40822 | -0.473233398 |
| Com_101_neg | 0.413984 | -0.57705 | -0.30153 | 0.455013 | 0.542401 | 0.490234 | 0.587579 | 0.233807 | 0.530570669 |
| Com_508_neg | -0.46462 | 0.677276 | 0.224785 | -0.602 | -0.67549 | -0.57388 | -0.50898 | -0.39146 | -0.447578053 |
| Com_260_pos | -0.3642 | 0.627071 | 0.392391 | -0.57746 | -0.62357 | -0.56537 | -0.59116 | -0.28849 | -0.549892591 |
| Com_3273_neg | -0.48699 | 0.686247 | 0.36464 | -0.71182 | -0.5552 | -0.39182 | -0.54855 | -0.43333 | -0.556980419 |
| Com_1503_neg | -0.53968 | 0.601828 | 0.345232 | -0.71282 | -0.55798 | -0.4209 | -0.63694 | -0.3383 | -0.642079467 |
| Com_295_pos | -0.55894 | 0.608881 | 0.368164 | -0.72729 | -0.43627 | -0.41962 | -0.63514 | -0.54035 | -0.584364303 |
| Com_2609_pos | -0.55084 | 0.589538 | 0.291151 | -0.7294 | -0.46461 | -0.40812 | -0.59223 | -0.51768 | -0.575382557 |
| Com_2200_neg | 0.439872 | -0.47189 | -0.42913 | 0.7484 | 0.485275 | 0.454869 | 0.427551 | 0.411795 | 0.363665877 |
| Com_829_neg | -0.48504 | 0.711168 | 0.389511 | -0.69161 | -0.5023 | -0.41683 | -0.58535 | -0.50708 | -0.548667484 |
| Com_2636_neg | -0.39372 | 0.762758 | 0.304733 | -0.65816 | -0.55102 | -0.42423 | -0.46465 | -0.48484 | -0.444918358 |
| Com_383_pos | -0.55163 | 0.61146 | 0.489469 | -0.62322 | -0.484 | -0.49967 | -0.51834 | -0.43649 | -0.581304555 |
| Com_1672_neg | -0.43836 | 0.673787 | 0.252535 | -0.63695 | -0.53515 | -0.50028 | -0.57052 | -0.38418 | -0.470659112 |
| Com_1082_neg | 0.430319 | -0.62812 | -0.41269 | 0.426239 | 0.663379 | 0.603928 | 0.450077 | 0.206158 | 0.45218636 |
| Com_262_pos | -0.39389 | 0.651753 | 0.460155 | -0.57395 | -0.55404 | -0.51378 | -0.57732 | -0.29415 | -0.593591728 |
| Com_620_pos | 0.333903 | -0.59825 | -0.22133 | 0.517403 | 0.418426 | 0.540758 | 0.507085 | 0.63628 | 0.335600637 |
| Com_683_neg | -0.45018 | 0.727526 | 0.411735 | -0.69933 | -0.4525 | -0.3435 | -0.50564 | -0.54964 | -0.484872078 |
| Com_4633_pos | -0.50862 | 0.632574 | 0.27369 | -0.65109 | -0.507 | -0.55577 | -0.64965 | -0.40474 | -0.621365681 |
| Com_2633_pos | -0.49206 | 0.678921 | 0.354596 | -0.69154 | -0.49553 | -0.35905 | -0.56698 | -0.37271 | -0.604698432 |
| Com_339_neg | 0.783885 | -0.25882 | -0.15588 | 0.635958 | 0.222441 | 0.47684 | 0.407103 | 0.573011 | 0.334832686 |
| Com_11428_pos | -0.52964 | 0.428113 | 0.312367 | -0.66679 | -0.54515 | -0.46408 | -0.52798 | -0.42552 | -0.437476931 |
| Com_12307_pos | -0.40799 | 0.556979 | 0.326971 | -0.56579 | -0.47253 | -0.58938 | -0.49069 | -0.55082 | -0.41428274 |
| Com_1827_neg | -0.52423 | 0.656501 | 0.314057 | -0.73208 | -0.42496 | -0.42674 | -0.58859 | -0.51663 | -0.568115876 |
| Com_2532_neg | -0.56208 | 0.469197 | 0.530975 | -0.55398 | -0.41435 | -0.39755 | -0.59015 | -0.58722 | -0.498162143 |
| Com_1031_neg | -0.50588 | 0.620756 | 0.340482 | -0.72288 | -0.48141 | -0.30212 | -0.54745 | -0.4704 | -0.571383884 |
| Com_955_neg | -0.53792 | 0.467315 | 0.193511 | -0.47826 | -0.4123 | -0.61556 | -0.43309 | -0.67683 | -0.236238301 |
| Com_137_neg | -0.53971 | 0.608048 | 0.300751 | -0.75315 | -0.48336 | -0.40462 | -0.63502 | -0.46805 | -0.596111782 |
| Com_1660_neg | -0.39087 | 0.768157 | 0.491168 | -0.59141 | -0.47324 | -0.23483 | -0.37532 | -0.54489 | -0.44410524 |
| Com_1844_pos | -0.46993 | 0.638049 | 0.373034 | -0.70153 | -0.41311 | -0.30341 | -0.53818 | -0.49326 | -0.517362267 |
| Com_337_neg | -0.52337 | 0.635566 | 0.313654 | -0.63165 | -0.51883 | -0.59711 | -0.60062 | -0.37057 | -0.586416858 |
| Com_4071_pos | 0.39738 | -0.27587 | -0.0823 | 0.384746 | 0.443104 | 0.40735 | 0.216167 | 0.64744 | -0.091449514 |

Continued

| ID | Prot_A0A0G2K7I9 | Prot_A0A0G2K7W2 | Prot_A0A0G2K8R3 | Prot_A0A0G2K8Z9 | Prot_A0A0G2K9C0 | Prot_A0A0G2K9C8 | Prot_A0A0G2K9F0 | Prot_A0A0G2K9H9 | Prot_A0A0G2K9L6 |
| --- | --- | --- | --- | --- | --- | --- | --- | --- | --- |
| Com_2013_pos | -0.478079868 | -0.522360936 | 0.49219205 | -0.574032696 | -0.749736234 | -0.550413606 | -0.007291863 | -0.506368677 | -0.457807725 |
| Com_23145_pos | -0.39501056 | -0.401460459 | 0.662297616 | -0.350823805 | -0.624331677 | -0.561829124 | -0.103674066 | -0.536333293 | -0.369638029 |
| Com_3764_pos | -0.53020775 | -0.372397389 | 0.459610614 | -0.418654067 | -0.692202253 | -0.384895751 | -0.059686928 | -0.471341064 | -0.387914859 |
| Com_692_pos | 0.52458106 | 0.287202066 | -0.575970833 | 0.436513485 | 0.516655209 | 0.329161271 | -0.132185896 | 0.352892223 | 0.612525295 |
| Com_9745_pos | 0.640976084 | 0.205023509 | -0.638855959 | 0.451297499 | 0.513227172 | 0.218206462 | -0.177472114 | 0.331901897 | 0.519327498 |
| Com_6269_pos | -0.61299294 | -0.473734085 | 0.368086605 | -0.465184319 | -0.775269236 | -0.454991115 | -0.038626013 | -0.48170595 | -0.328389069 |
| Com_165_neg | 0.458840968 | 0.28285127 | -0.646171333 | 0.320472612 | 0.557089141 | 0.65545437 | 0.156439684 | 0.29484398 | 0.41054463 |
| Com_1512_pos | 0.490820527 | 0.196436305 | -0.614887647 | 0.223107352 | 0.54635057 | 0.547092104 | 0.316991697 | 0.131304277 | 0.192351877 |
| Com_1241_neg | -0.499699791 | -0.463311769 | 0.423142593 | -0.413429626 | -0.687170104 | -0.421490757 | -0.029193545 | -0.478736372 | -0.337154336 |
| Com_780_pos | -0.454209132 | -0.421859598 | 0.511994143 | -0.469272323 | -0.754188618 | -0.589705289 | -0.111337119 | -0.510283562 | -0.36727393 |
| Com_9406_pos | -0.518334777 | -0.341550795 | 0.387622189 | -0.315322625 | -0.609639556 | -0.378608319 | -0.042141246 | -0.402950928 | -0.36223254 |
| Com_308_pos | -0.485040176 | -0.389325087 | 0.417929708 | -0.355093337 | -0.661551738 | -0.427073694 | -0.102258183 | -0.429466211 | -0.352810377 |
| Com_8285_pos | 0.135136206 | 0.42000438 | -0.526425485 | 0.421085739 | 0.653152442 | 0.314168997 | 0.009050814 | 0.64158586 | 0.289968868 |
| Com_1214_neg | -0.454649788 | -0.391620128 | 0.441648334 | -0.361601443 | -0.651938638 | -0.406068141 | -0.1009977 | -0.458072994 | -0.364677412 |
| Com_3075_neg | -0.487596335 | -0.432014343 | 0.285822371 | -0.368855228 | -0.652548849 | -0.356588747 | -0.049428569 | -0.448077627 | -0.279834429 |
| Com_619_pos | -0.424696626 | -0.369027867 | 0.679141031 | -0.36021186 | -0.715292581 | -0.67747068 | -0.283640533 | -0.438701842 | -0.417086803 |
| Com_1617_neg | -0.239082548 | -0.535318822 | 0.468955138 | -0.447425364 | -0.629641657 | -0.450998196 | -0.174879783 | -0.500176726 | -0.423089477 |
| Com_909_pos | -0.454485751 | -0.398294652 | 0.415059889 | -0.35802455 | -0.657912552 | -0.44116009 | -0.120005622 | -0.429247529 | -0.346254158 |
| Com_433_neg | -0.427177506 | -0.358123418 | 0.503555869 | -0.394906271 | -0.671241762 | -0.462424652 | -0.140746654 | -0.485226901 | -0.365939401 |
| Com_3856_pos | -0.546322038 | -0.453516266 | 0.57357133 | -0.604714857 | -0.710743779 | -0.43158224 | 0.094849676 | -0.475740386 | -0.455489109 |
| Com_101_neg | 0.439542636 | 0.243746174 | -0.572024325 | 0.244142861 | 0.58305052 | 0.590603797 | 0.186852073 | 0.286769298 | 0.239601544 |
| Com_508_neg | -0.502518917 | -0.501166682 | 0.411087503 | -0.511451818 | -0.778327169 | -0.491266072 | -0.097672794 | -0.559582917 | -0.306354059 |
| Com_260_pos | -0.455832105 | -0.418936719 | 0.392169393 | -0.256995622 | -0.573175994 | -0.503718163 | -0.196967957 | -0.359144083 | -0.40261708 |
| Com_3273_neg | -0.436043416 | -0.406152761 | 0.383341543 | -0.369201995 | -0.636062078 | -0.448540337 | -0.125527567 | -0.434045784 | -0.312098635 |
| Com_1503_neg | -0.387138418 | -0.462704906 | 0.45011666 | -0.351542564 | -0.648586721 | -0.616854177 | -0.288602464 | -0.400402464 | -0.315028973 |
| Com_295_pos | -0.445676011 | -0.331161982 | 0.538551565 | -0.357100425 | -0.654003166 | -0.483484502 | -0.179306782 | -0.427027467 | -0.372441441 |
| Com_2609_pos | -0.413911139 | -0.304356795 | 0.487713328 | -0.35156565 | -0.635303559 | -0.477987708 | -0.187303283 | -0.429030447 | -0.375553631 |
| Com_2200_neg | 0.565971945 | 0.127465411 | -0.474372267 | 0.204512707 | 0.48268455 | 0.321412555 | 0.288050428 | 0.195213245 | 0.321993718 |
| Com_829_neg | -0.4625812 | -0.420652604 | 0.406902148 | -0.349283615 | -0.644006907 | -0.409573409 | -0.0724046 | -0.445004148 | -0.318065698 |
| Com_2636_neg | -0.450470938 | -0.374439781 | 0.279715211 | -0.281241991 | -0.576395547 | -0.298097714 | 0.001609545 | -0.464664004 | -0.26592521 |
| Com_383_pos | -0.646387435 | -0.509254274 | 0.568963693 | -0.464850617 | -0.821751319 | -0.595478449 | -0.230784144 | -0.419010351 | -0.325002633 |
| Com_1672_neg | -0.482885379 | -0.46181829 | 0.217729229 | -0.380756175 | -0.634558837 | -0.349452634 | -0.080327905 | -0.4054933 | -0.219532268 |
| Com_1082_neg | 0.603535397 | 0.309535256 | -0.646547779 | 0.380216558 | 0.71589906 | 0.600467044 | 0.085115422 | 0.397955466 | 0.274182962 |
| Com_262_pos | -0.491316918 | -0.431868198 | 0.362388397 | -0.290547184 | -0.619401652 | -0.498958963 | -0.177772953 | -0.349522403 | -0.340362925 |
| Com_620_pos | 0.648881993 | 0.278628032 | -0.458454583 | 0.499582485 | 0.75170866 | 0.260455475 | 0.04617978 | 0.363594309 | 0.497828878 |
| Com_683_neg | -0.465276452 | -0.382241652 | 0.368225404 | -0.358659391 | -0.615765756 | -0.312084599 | 0.000152543 | -0.443665553 | -0.330817132 |
| Com_4633_pos | -0.442502424 | -0.405364622 | 0.367856676 | -0.300327481 | -0.656093896 | -0.519906891 | -0.211096006 | -0.437165463 | -0.317044065 |
| Com_2633_pos | -0.486119377 | -0.387921615 | 0.383889735 | -0.454432856 | -0.677181268 | -0.50565783 | -0.159355581 | -0.37651447 | -0.372072219 |
| Com_339_neg | 0.125365355 | 0.384408357 | -0.614368888 | 0.304853476 | 0.649364475 | 0.460542738 | 0.031440627 | 0.777466307 | 0.194200429 |
| Com_11428_pos | -0.495742082 | -0.280112115 | 0.66561225 | -0.347412347 | -0.672618732 | -0.555613325 | -0.340282803 | -0.282123374 | -0.317487978 |
| Com_12307_pos | -0.640944762 | -0.113900041 | 0.592647981 | -0.197966796 | -0.625669339 | -0.377237716 | -0.21258559 | -0.272300622 | -0.327151139 |
| Com_1827_neg | -0.452050793 | -0.361686649 | 0.361873976 | -0.296668123 | -0.648125017 | -0.424532499 | -0.192191284 | -0.411939834 | -0.260584941 |
| Com_2532_neg | -0.31600808 | -0.308030223 | 0.847776561 | -0.267533941 | -0.500558907 | -0.463303035 | 0.025719759 | -0.53855139 | -0.396449925 |
| Com_1031_neg | -0.338450248 | -0.394583121 | 0.373811272 | -0.304494553 | -0.583517252 | -0.439656995 | -0.153973364 | -0.40724061 | -0.277902459 |
| Com_955_neg | -0.446192593 | -0.241173529 | 0.704594643 | -0.340272494 | -0.727232439 | -0.328423173 | 0.029449035 | -0.62361469 | -0.190527585 |
| Com_137_neg | -0.373927266 | -0.408080296 | 0.381595595 | -0.326698328 | -0.613380411 | -0.475910242 | -0.219534085 | -0.419351461 | -0.308327301 |
| Com_1660_neg | -0.509976599 | -0.357546909 | 0.445817232 | -0.458646786 | -0.639352764 | -0.28729484 | 0.138911733 | -0.465007852 | -0.344686284 |
| Com_1844_pos | -0.410660292 | -0.334285646 | 0.37267123 | -0.361806488 | -0.626516994 | -0.373520468 | -0.116717193 | -0.368583347 | -0.303659713 |
| Com_337_neg | -0.560253714 | -0.348261721 | 0.44985013 | -0.291301347 | -0.726820805 | -0.565833065 | -0.277828937 | -0.373623317 | -0.224692633 |
| Com_4071_pos | 0.276122828 | 0.111789971 | -0.661789429 | 0.320409249 | 0.504387076 | 0.064246032 | -0.21694911 | 0.50636089 | 0.139470681 |

Continued

| ID | Prot_A0A0G2KA88 | Prot_A0A0G2KAS8 | Prot_A0A0G2KAX2 | Prot_A0A0G2KBB3 | Prot_A0A0G2QC15 | Prot_A0A0G3F9V8 | Prot_A0A0H2UH94 | Prot_A0A0H2UH97 | Prot_A0A0H2UHM3 |
| --- | --- | --- | --- | --- | --- | --- | --- | --- | --- |
| Com_2013_pos | 0.579631562 | -0.484036169 | -0.426449502 | -0.351454559 | -0.710884525 | 0.426532526 | -0.337585454 | -0.453190457 | -0.570727933 |
| Com_23145_pos | 0.732426762 | -0.421617998 | -0.4496838 | -0.445734377 | -0.653337362 | 0.165063597 | -0.440794449 | -0.438998908 | -0.5453409 |
| Com_3764_pos | 0.784803875 | -0.36994667 | -0.463778848 | -0.405619292 | -0.733398686 | 0.237323002 | -0.357695091 | -0.296679158 | -0.42036508 |
| Com_692_pos | -0.427089098 | 0.510772662 | 0.7662178 | 0.107612497 | 0.722249455 | -0.353457712 | 0.471061195 | 0.512060304 | 0.334546069 |
| Com_9745_pos | -0.358014172 | 0.393883076 | 0.808498655 | 0.147681318 | 0.624496605 | -0.300198185 | 0.61374406 | 0.514312811 | 0.177230476 |
| Com_6269_pos | 0.674492296 | -0.400489967 | -0.43093001 | -0.411129944 | -0.653264079 | 0.360856115 | -0.380486765 | -0.383073537 | -0.517559032 |
| Com_165_neg | -0.409462195 | 0.507546372 | 0.471632503 | 0.189788114 | 0.682205376 | -0.499241302 | 0.334091966 | 0.603416 | 0.674568014 |
| Com_1512_pos | -0.573736266 | 0.503380382 | 0.438527569 | 0.125203596 | 0.714100604 | -0.465724766 | 0.297683341 | 0.587006476 | 0.678093089 |
| Com_1241_neg | 0.679395466 | -0.423012429 | -0.459962814 | -0.42433268 | -0.628281764 | 0.349109933 | -0.326285831 | -0.327036483 | -0.527657587 |
| Com_780_pos | 0.677460076 | -0.470691282 | -0.305770283 | -0.399765657 | -0.691147099 | 0.230234812 | -0.380448346 | -0.492852452 | -0.614298229 |
| Com_9406_pos | 0.623353887 | -0.336540218 | -0.460024197 | -0.407410403 | -0.619193799 | 0.393506329 | -0.206927878 | -0.24227746 | -0.477571829 |
| Com_308_pos | 0.679929185 | -0.424908199 | -0.434482554 | -0.451051306 | -0.629710424 | 0.3170713 | -0.30341909 | -0.297193353 | -0.540918745 |
| Com_8285_pos | -0.52900338 | 0.467707976 | 0.348241924 | 0.294181236 | 0.561655997 | -0.113843521 | 0.381891577 | 0.292564578 | 0.39878713 |
| Com_1214_neg | 0.719401021 | -0.437610691 | -0.429427055 | -0.464327948 | -0.644895557 | 0.243574042 | -0.338859768 | -0.288466218 | -0.512619643 |
| Com_3075_neg | 0.722033433 | -0.393122661 | -0.394381834 | -0.415325025 | -0.568883904 | 0.301178439 | -0.278647551 | -0.256013886 | -0.503130247 |
| Com_619_pos | 0.587932566 | -0.516259914 | -0.331315747 | -0.443013036 | -0.770669347 | 0.189407499 | -0.49867876 | -0.511364667 | -0.622538058 |
| Com_1617_neg | 0.672774168 | -0.546444709 | -0.44932796 | -0.514629382 | -0.554678976 | 0.159940298 | -0.460964463 | -0.25435298 | -0.507353026 |
| Com_909_pos | 0.699442474 | -0.428454959 | -0.405045303 | -0.453055252 | -0.633283429 | 0.299934224 | -0.291711522 | -0.289683381 | -0.551728615 |
| Com_433_neg | 0.714318424 | -0.448040603 | -0.333554314 | -0.446572934 | -0.678011045 | 0.156013514 | -0.392445063 | -0.362817836 | -0.515931741 |
| Com_3856_pos | 0.449682923 | -0.308488984 | -0.510697153 | -0.372906015 | -0.661499406 | 0.446264193 | -0.39414947 | -0.355228577 | -0.344599304 |
| Com_101_neg | -0.472286865 | 0.541857755 | 0.338690141 | 0.141962846 | 0.67926091 | -0.47052279 | 0.229187397 | 0.62672804 | 0.761572816 |
| Com_508_neg | 0.728960501 | -0.440383533 | -0.439820084 | -0.343279602 | -0.607897334 | 0.264221548 | -0.489898379 | -0.448537098 | -0.4998525 |
| Com_260_pos | 0.648822398 | -0.393423311 | -0.450398863 | -0.441354067 | -0.658919913 | 0.400204177 | -0.239022995 | -0.221861671 | -0.54762171 |
| Com_3273_neg | 0.730952044 | -0.400298942 | -0.382266884 | -0.42791879 | -0.59621541 | 0.313632377 | -0.276126143 | -0.277850631 | -0.548443437 |
| Com_1503_neg | 0.697085256 | -0.490216383 | -0.354448241 | -0.412707723 | -0.633059962 | 0.364201278 | -0.342523683 | -0.36443885 | -0.670988729 |
| Com_295_pos | 0.645091789 | -0.476242901 | -0.377585346 | -0.457731557 | -0.660253531 | 0.216830757 | -0.400905999 | -0.385244684 | -0.556765618 |
| Com_2609_pos | 0.693403794 | -0.443819454 | -0.301928454 | -0.411570415 | -0.700819629 | 0.198357939 | -0.319411358 | -0.347061178 | -0.539938482 |
| Com_2200_neg | -0.625723913 | 0.29195401 | 0.5429887 | 0.412409224 | 0.581581339 | -0.278521546 | 0.470889459 | 0.178714297 | 0.321681915 |
| Com_829_neg | 0.665423893 | -0.414299377 | -0.397423359 | -0.458177071 | -0.607532644 | 0.296573604 | -0.299423658 | -0.284033272 | -0.530504999 |
| Com_2636_neg | 0.766949941 | -0.360634012 | -0.390936664 | -0.411310772 | -0.543082281 | 0.20012385 | -0.242317071 | -0.225353357 | -0.468943701 |
| Com_383_pos | 0.580022616 | -0.410517751 | -0.426113319 | -0.511906846 | -0.704645672 | 0.254533912 | -0.666823054 | -0.464754158 | -0.475227457 |
| Com_1672_neg | 0.612543836 | -0.326784132 | -0.365742975 | -0.390316381 | -0.486569348 | 0.421095383 | -0.24556811 | -0.191628543 | -0.464635494 |
| Com_1082_neg | -0.442767317 | 0.425300831 | 0.438945634 | 0.159909081 | 0.722423519 | -0.470742682 | 0.425934016 | 0.664387409 | 0.596023891 |
| Com_262_pos | 0.616020521 | -0.341576012 | -0.406771425 | -0.487700814 | -0.600285459 | 0.429393376 | -0.215794598 | -0.209974537 | -0.549272565 |
| Com_620_pos | -0.551260321 | 0.478157524 | 0.490472458 | 0.362099571 | 0.791244056 | -0.159450046 | 0.507229372 | 0.409054378 | 0.30451284 |
| Com_683_neg | 0.661653072 | -0.380176434 | -0.423757249 | -0.466321484 | -0.56138116 | 0.223189864 | -0.317310296 | -0.235862076 | -0.439374832 |
| Com_4633_pos | 0.680874062 | -0.400762597 | -0.271780358 | -0.458292648 | -0.645573951 | 0.294501418 | -0.243203807 | -0.286667774 | -0.590074587 |
| Com_2633_pos | 0.665644395 | -0.45748537 | -0.357852761 | -0.386271012 | -0.638010903 | 0.358528372 | -0.31242232 | -0.371292107 | -0.583120651 |
| Com_339_neg | -0.218425757 | 0.20384712 | 0.017483994 | 0.315894829 | 0.520456147 | 0.162882798 | 0.601176509 | 0.325011376 | 0.180645132 |
| Com_11428_pos | 0.539387589 | -0.511222786 | -0.439881512 | -0.230333681 | -0.812670639 | 0.374971664 | -0.521880823 | -0.499443652 | -0.543352476 |
| Com_12307_pos | 0.633771925 | -0.419532373 | -0.424135853 | -0.355336497 | -0.806450556 | 0.179386573 | -0.417165677 | -0.442210932 | -0.464148944 |
| Com_1827_neg | 0.723693658 | -0.42051928 | -0.321099758 | -0.478956732 | -0.579521085 | 0.21104053 | -0.323594371 | -0.282451583 | -0.556807935 |
| Com_2532_neg | 0.377471706 | -0.360035056 | -0.416428033 | -0.477110303 | -0.586044855 | 0.059251846 | -0.494764282 | -0.432092808 | -0.386856248 |
| Com_1031_neg | 0.704455587 | -0.391356528 | -0.289188373 | -0.421524072 | -0.602726067 | 0.279197612 | -0.194818185 | -0.218736083 | -0.556092314 |
| Com_955_neg | 0.43093135 | -0.376164624 | -0.354300324 | -0.272510051 | -0.595809036 | -0.055143177 | -0.639907933 | -0.587928386 | -0.312874959 |
| Com_137_neg | 0.716041192 | -0.42938099 | -0.324774562 | -0.46430428 | -0.589100709 | 0.274849354 | -0.285531008 | -0.246752629 | -0.571576285 |
| Com_1660_neg | 0.646012123 | -0.356358063 | -0.457131542 | -0.422698696 | -0.552811933 | 0.165352565 | -0.359646879 | -0.355916944 | -0.386999316 |
| Com_1844_pos | 0.629414567 | -0.419633081 | -0.370079898 | -0.404998677 | -0.571219102 | 0.310143207 | -0.248872762 | -0.261826509 | -0.525148241 |
| Com_337_neg | 0.657354064 | -0.442839978 | -0.323618403 | -0.382624545 | -0.674142458 | 0.354283539 | -0.363065243 | -0.447254976 | -0.653313838 |
| Com_4071_pos | -0.186451691 | 0.241265165 | 0.366011069 | -0.045993555 | 0.556512251 | -0.082576168 | 0.439946774 | 0.41487811 | 0.058967298 |

Continued

| ID | Prot_A0A0H2UHS9 | Prot_A0A0H2UHW2 | Prot_A0A140TAC4 | Prot_A0A140TAI6 | Prot_A0A1B0GWY3 | Prot_A0A1K0FUA6 | Prot_A0A1W2Q680 | Prot_A1A5P9 | Prot_A1A5S1 |
| --- | --- | --- | --- | --- | --- | --- | --- | --- | --- |
| Com_2013_pos | 0.647779684 | -0.480667346 | 0.397450047 | 0.762395445 | -0.336166991 | -0.476660105 | -0.677169149 | -0.398940422 | -0.699441746 |
| Com_23145_pos | 0.531135818 | -0.525539006 | 0.477259136 | 0.813567494 | -0.424971301 | -0.616222919 | -0.591152587 | -0.416489587 | -0.701918894 |
| Com_3764_pos | 0.552643056 | -0.583623929 | 0.469356152 | 0.746539367 | -0.444480433 | -0.633589637 | -0.520542616 | -0.427046951 | -0.612338446 |
| Com_692_pos | -0.515313699 | 0.566405387 | -0.334920661 | -0.631684042 | 0.418632997 | 0.306343178 | 0.314352125 | 0.488430323 | 0.510856049 |
| Com_9745_pos | -0.384482082 | 0.693577484 | -0.471389011 | -0.575509822 | 0.566668137 | 0.34576367 | 0.380335012 | 0.620377661 | 0.420588849 |
| Com_6269_pos | 0.60935283 | -0.634390641 | 0.427399173 | 0.727836466 | -0.308108195 | -0.54530597 | -0.629699687 | -0.517984631 | -0.563415955 |
| Com_165_neg | -0.688343333 | 0.365503678 | -0.227935065 | -0.818279494 | 0.250218814 | 0.355173637 | 0.58982873 | 0.353755027 | 0.682769109 |
| Com_1512_pos | -0.631544383 | 0.387852454 | -0.503783476 | -0.852277596 | 0.219430099 | 0.366654703 | 0.593087301 | 0.237350514 | 0.739808312 |
| Com_1241_neg | 0.618586147 | -0.587151446 | 0.423232067 | 0.701230518 | -0.369712383 | -0.556312338 | -0.626542438 | -0.484763094 | -0.601788797 |
| Com_780_pos | 0.520884973 | -0.480081497 | 0.356758982 | 0.775367707 | -0.317638862 | -0.612058333 | -0.703120615 | -0.436977707 | -0.706504111 |
| Com_9406_pos | 0.72870194 | -0.519727501 | 0.303728444 | 0.668618609 | -0.371612912 | -0.466212034 | -0.553616081 | -0.403743929 | -0.54989949 |
| Com_308_pos | 0.656189434 | -0.525688443 | 0.357627584 | 0.699215772 | -0.37983411 | -0.568006194 | -0.620342476 | -0.475366987 | -0.612155882 |
| Com_8285_pos | -0.492911588 | 0.44577075 | -0.420463897 | -0.435255485 | 0.480745934 | 0.744569392 | 0.464544466 | 0.474564222 | 0.66499346 |
| Com_1214_neg | 0.598533904 | -0.545034916 | 0.400326738 | 0.695326106 | -0.40493754 | -0.614498629 | -0.614432468 | -0.477309456 | -0.639844377 |
| Com_3075_neg | 0.561558589 | -0.585883148 | 0.375976195 | 0.630871175 | -0.277392399 | -0.567639187 | -0.589164556 | -0.461714238 | -0.535357254 |
| Com_619_pos | 0.606790696 | -0.404444673 | 0.391445618 | 0.873141926 | -0.396109652 | -0.61689312 | -0.630559825 | -0.454225476 | -0.792917207 |
| Com_1617_neg | 0.59270032 | -0.38883829 | 0.515610719 | 0.648557993 | -0.413552893 | -0.612382213 | -0.548858415 | -0.450698951 | -0.700454266 |
| Com_909_pos | 0.636107681 | -0.496301589 | 0.359687789 | 0.708358485 | -0.36942508 | -0.582798819 | -0.617798949 | -0.44714773 | -0.626107501 |
| Com_433_neg | 0.506110951 | -0.51879269 | 0.408176329 | 0.720320708 | -0.390838471 | -0.669708169 | -0.665603967 | -0.448899594 | -0.709321541 |
| Com_3856_pos | 0.580214059 | -0.504235566 | 0.44914758 | 0.712656423 | -0.578318397 | -0.481904155 | -0.603256625 | -0.466315329 | -0.561039669 |
| Com_101_neg | -0.650027996 | 0.427698817 | -0.251294054 | -0.753873489 | 0.153024579 | 0.402665321 | 0.707317466 | 0.335740834 | 0.742947511 |
| Com_508_neg | 0.583729132 | -0.596796355 | 0.575681549 | 0.707674809 | -0.23291819 | -0.575456673 | -0.552154717 | -0.430506409 | -0.626454383 |
| Com_260_pos | 0.820803155 | -0.41912951 | 0.338135205 | 0.782136944 | -0.261193413 | -0.410725198 | -0.45106143 | -0.304279968 | -0.608745617 |
| Com_3273_neg | 0.608931898 | -0.48375901 | 0.372332268 | 0.707854826 | -0.31324655 | -0.590078726 | -0.606248963 | -0.392336836 | -0.610335891 |
| Com_1503_neg | 0.703581388 | -0.387026879 | 0.413319802 | 0.821376934 | -0.206897892 | -0.558263603 | -0.597492328 | -0.329957631 | -0.714873744 |
| Com_295_pos | 0.584437758 | -0.4946249 | 0.385851645 | 0.730393447 | -0.410840044 | -0.620225961 | -0.696743213 | -0.485523759 | -0.716538787 |
| Com_2609_pos | 0.562863921 | -0.451426668 | 0.341089228 | 0.730946806 | -0.349154984 | -0.630760761 | -0.648067463 | -0.375329319 | -0.727103724 |
| Com_2200_neg | -0.567799519 | 0.486881425 | -0.393095184 | -0.713138072 | 0.500192179 | 0.644214102 | 0.398165246 | 0.508267978 | 0.46675147 |
| Com_829_neg | 0.591571657 | -0.550603495 | 0.369016737 | 0.679285727 | -0.36680443 | -0.571662487 | -0.636441474 | -0.481793386 | -0.60050863 |
| Com_2636_neg | 0.475253857 | -0.622464212 | 0.320518238 | 0.585300907 | -0.278770925 | -0.584434926 | -0.53640583 | -0.460250175 | -0.489763905 |
| Com_383_pos | 0.548507602 | -0.588588719 | 0.636909657 | 0.886431451 | -0.419928188 | -0.56840594 | -0.592892336 | -0.589416331 | -0.619463356 |
| Com_1672_neg | 0.647962772 | -0.524569616 | 0.401154363 | 0.590576095 | -0.216108818 | -0.481864322 | -0.558558217 | -0.393286184 | -0.486450901 |
| Com_1082_neg | -0.605635693 | 0.567799211 | -0.375494755 | -0.806345929 | 0.288716544 | 0.46562287 | 0.610902112 | 0.461650774 | 0.638622828 |
| Com_262_pos | 0.810094679 | -0.410999678 | 0.339290091 | 0.760424141 | -0.298449576 | -0.407918737 | -0.531854331 | -0.341035333 | -0.564975758 |
| Com_620_pos | -0.507246724 | 0.625868307 | -0.515683811 | -0.626098973 | 0.504744373 | 0.478493086 | 0.596361757 | 0.564909292 | 0.64411572 |
| Com_683_neg | 0.484509476 | -0.579713597 | 0.336317283 | 0.597392091 | -0.432352984 | -0.600412371 | -0.5991705 | -0.547522592 | -0.513375271 |
| Com_4633_pos | 0.719947176 | -0.453886022 | 0.339263731 | 0.740338618 | -0.208790412 | -0.512831024 | -0.616777263 | -0.32087512 | -0.679315458 |
| Com_2633_pos | 0.599053332 | -0.434513605 | 0.339514949 | 0.739630987 | -0.274452337 | -0.542987155 | -0.678885949 | -0.391420209 | -0.650753207 |
| Com_339_neg | -0.22103014 | 0.456003002 | -0.264853835 | -0.417300581 | 0.412991856 | 0.815005248 | 0.247320294 | 0.53088202 | 0.494617166 |
| Com_11428_pos | 0.613328334 | -0.438697301 | 0.509862362 | 0.857041456 | -0.39423364 | -0.632813207 | -0.506061355 | -0.431804922 | -0.749731228 |
| Com_12307_pos | 0.53868809 | -0.622594595 | 0.420064316 | 0.773421222 | -0.456332619 | -0.567175413 | -0.60759048 | -0.510306201 | -0.678629644 |
| Com_1827_neg | 0.572673482 | -0.519827772 | 0.372160451 | 0.67501327 | -0.301044984 | -0.623098863 | -0.645177684 | -0.46718664 | -0.615832402 |
| Com_2532_neg | 0.417946906 | -0.528774163 | 0.39848635 | 0.662845749 | -0.637065591 | -0.532073768 | -0.628464966 | -0.537579799 | -0.67225032 |
| Com_1031_neg | 0.575124887 | -0.405306278 | 0.319940527 | 0.684939273 | -0.303706072 | -0.596643884 | -0.573756768 | -0.329026248 | -0.624521393 |
| Com_955_neg | 0.29638345 | -0.732762234 | 0.537700981 | 0.497427448 | -0.514495503 | -0.680967769 | -0.568597429 | -0.670392997 | -0.608368742 |
| Com_137_neg | 0.64607337 | -0.425804663 | 0.378028302 | 0.709006957 | -0.277513907 | -0.595114915 | -0.608421374 | -0.358599063 | -0.664812325 |
| Com_1660_neg | 0.31574039 | -0.62566384 | 0.386407749 | 0.584013512 | -0.515849405 | -0.56227856 | -0.666997419 | -0.576452427 | -0.486347404 |
| Com_1844_pos | 0.585632392 | -0.430997781 | 0.304740964 | 0.613604605 | -0.391218168 | -0.589119598 | -0.629194205 | -0.453641761 | -0.585150308 |
| Com_337_neg | 0.696281761 | -0.529096911 | 0.406823862 | 0.802662177 | -0.218783169 | -0.563067699 | -0.664388116 | -0.4363869 | -0.680878038 |
| Com_4071_pos | -0.131960664 | 0.604792366 | -0.410573002 | -0.264190744 | 0.597450718 | 0.630880583 | 0.28431339 | 0.521167009 | 0.433556132 |

Continued

| ID | Prot_A2IBE0 | Prot_A2RRT6 | Prot_A9UK05 | Prot_B0BMX3 | Prot_B0BN59 | Prot_B0BND5 | Prot_B0BNJ9 | Prot_B0BNN3 | Prot_B0K021 |
| --- | --- | --- | --- | --- | --- | --- | --- | --- | --- |
| Com_2013_pos | -0.65408 | 0.651666 | -0.5363 | 0.632856 | 0.4850726 | 0.520401 | -0.71423 | -0.52878 | -0.58173 |
| Com_23145_pos | -0.61483 | 0.551512 | -0.43285 | 0.493333 | 0.40426073 | 0.675027 | -0.61736 | -0.60649 | -0.54883 |
| Com_3764_pos | -0.726 | 0.638085 | -0.40643 | 0.666579 | 0.54686608 | 0.679801 | -0.59466 | -0.67921 | -0.4255 |
| Com_692_pos | 0.747603 | -0.50174 | 0.085269 | -0.59624 | -0.5160054 | -0.5099 | 0.824179 | 0.325975 | 0.149734 |
| Com_9745_pos | 0.701563 | -0.59151 | -0.0346 | -0.59962 | -0.5283201 | -0.56822 | 0.86574 | 0.305488 | 0.040156 |
| Com_6269_pos | -0.67103 | 0.580958 | -0.47653 | 0.596968 | 0.59497753 | 0.694918 | -0.63949 | -0.59726 | -0.52871 |
| Com_165_neg | 0.596696 | -0.43131 | 0.536562 | -0.44922 | -0.3434433 | -0.49589 | 0.65236 | 0.348585 | 0.57681 |
| Com_1512_pos | 0.509888 | -0.43163 | 0.528828 | -0.43667 | -0.4164979 | -0.54581 | 0.520956 | 0.35733 | 0.479699 |
| Com_1241_neg | -0.61309 | 0.584226 | -0.57482 | 0.537133 | 0.53931411 | 0.624067 | -0.62887 | -0.59845 | -0.52112 |
| Com_780_pos | -0.58626 | 0.558029 | -0.47111 | 0.557147 | 0.42780465 | 0.68699 | -0.55598 | -0.63548 | -0.64861 |
| Com_9406_pos | -0.66119 | 0.514215 | -0.62511 | 0.610188 | 0.55191602 | 0.540219 | -0.55249 | -0.5257 | -0.48053 |
| Com_308_pos | -0.59103 | 0.553075 | -0.61444 | 0.539643 | 0.50988128 | 0.606874 | -0.56065 | -0.60414 | -0.54997 |
| Com_8285_pos | 0.455155 | -0.70692 | 0.374298 | -0.51723 | -0.3141306 | -0.47076 | 0.516824 | 0.780046 | 0.332947 |
| Com_1214_neg | -0.58219 | 0.60224 | -0.57706 | 0.536579 | 0.51306997 | 0.618227 | -0.57303 | -0.64316 | -0.52716 |
| Com_3075_neg | -0.57951 | 0.547413 | -0.57073 | 0.469987 | 0.58640605 | 0.641388 | -0.56435 | -0.62157 | -0.50661 |
| Com_619_pos | -0.54516 | 0.586742 | -0.46608 | 0.562171 | 0.27933859 | 0.596833 | -0.54013 | -0.59936 | -0.65179 |
| Com_1617_neg | -0.43398 | 0.673971 | -0.52302 | 0.404848 | 0.36274344 | 0.43027 | -0.63328 | -0.61831 | -0.53215 |
| Com_909_pos | -0.57611 | 0.55253 | -0.61996 | 0.523494 | 0.48253872 | 0.600834 | -0.54008 | -0.61944 | -0.56794 |
| Com_433_neg | -0.55918 | 0.665236 | -0.51542 | 0.548607 | 0.47376774 | 0.636962 | -0.56321 | -0.67895 | -0.56375 |
| Com_3856_pos | -0.68786 | 0.719697 | -0.40712 | 0.71822 | 0.37376035 | 0.468682 | -0.73316 | -0.49816 | -0.41722 |
| Com_101_neg | 0.493671 | -0.41132 | 0.657891 | -0.4211 | -0.4384117 | -0.56934 | 0.526038 | 0.416617 | 0.6056 |
| Com_508_neg | -0.67253 | 0.650236 | -0.31095 | 0.55425 | 0.62128161 | 0.670409 | -0.70677 | -0.62067 | -0.46556 |
| Com_260_pos | -0.6515 | 0.491727 | -0.66206 | 0.533797 | 0.48876532 | 0.444175 | -0.5669 | -0.47243 | -0.54794 |
| Com_3273_neg | -0.58743 | 0.568899 | -0.6213 | 0.475098 | 0.49964036 | 0.599801 | -0.56067 | -0.62724 | -0.57359 |
| Com_1503_neg | -0.55169 | 0.57897 | -0.67017 | 0.403082 | 0.42399472 | 0.528277 | -0.59659 | -0.58209 | -0.67482 |
| Com_295_pos | -0.52097 | 0.63202 | -0.58413 | 0.526006 | 0.44834233 | 0.597834 | -0.56854 | -0.61398 | -0.58897 |
| Com_2609_pos | -0.56806 | 0.64316 | -0.57825 | 0.563101 | 0.46591308 | 0.582236 | -0.52411 | -0.65006 | -0.58204 |
| Com_2200_neg | 0.61604 | -0.63817 | 0.444314 | -0.43889 | -0.4061415 | -0.57682 | 0.58233 | 0.593315 | 0.355203 |
| Com_829_neg | -0.54543 | 0.560192 | -0.62574 | 0.492684 | 0.49297646 | 0.602884 | -0.55608 | -0.60344 | -0.55075 |
| Com_2636_neg | -0.57499 | 0.465343 | -0.52231 | 0.423383 | 0.59538271 | 0.700108 | -0.50751 | -0.63714 | -0.45815 |
| Com_383_pos | -0.57819 | 0.646372 | -0.2967 | 0.576621 | 0.36677983 | 0.661033 | -0.65098 | -0.54262 | -0.53955 |
| Com_1672_neg | -0.55249 | 0.589658 | -0.63072 | 0.450276 | 0.57462576 | 0.495587 | -0.58525 | -0.5427 | -0.49021 |
| Com_1082_neg | 0.674336 | -0.50833 | 0.371148 | -0.57891 | -0.4369697 | -0.67912 | 0.649907 | 0.471255 | 0.483738 |
| Com_262_pos | -0.60075 | 0.461361 | -0.69965 | 0.53978 | 0.4509388 | 0.460133 | -0.51105 | -0.46438 | -0.59234 |
| Com_620_pos | 0.609744 | -0.7155 | 0.215331 | -0.83275 | -0.6155974 | -0.56845 | 0.619016 | 0.505028 | 0.284257 |
| Com_683_neg | -0.52996 | 0.53986 | -0.52873 | 0.476122 | 0.48200607 | 0.637405 | -0.5359 | -0.62292 | -0.47427 |
| Com_4633_pos | -0.57648 | 0.556576 | -0.68564 | 0.548854 | 0.53005807 | 0.52661 | -0.49968 | -0.57184 | -0.63797 |
| Com_2633_pos | -0.57816 | 0.604197 | -0.60106 | 0.50528 | 0.49569063 | 0.570975 | -0.60177 | -0.57083 | -0.62311 |
| Com_339_neg | 0.426696 | -0.67473 | -0.0227 | -0.48993 | -7.32E-05 | -0.49171 | 0.370998 | 0.801522 | 0.295523 |
| Com_11428_pos | -0.59966 | 0.719367 | -0.42056 | 0.545989 | 0.32255227 | 0.552813 | -0.62934 | -0.60866 | -0.4521 |
| Com_12307_pos | -0.60003 | 0.558292 | -0.41659 | 0.671497 | 0.52939134 | 0.712316 | -0.47535 | -0.55569 | -0.40681 |
| Com_1827_neg | -0.497 | 0.544784 | -0.61714 | 0.44675 | 0.5061122 | 0.643356 | -0.48077 | -0.64478 | -0.59009 |
| Com_2532_neg | -0.42217 | 0.567521 | -0.39257 | 0.501877 | 0.19992177 | 0.496991 | -0.57051 | -0.46154 | -0.43902 |
| Com_1031_neg | -0.51158 | 0.534235 | -0.6788 | 0.435962 | 0.39724005 | 0.534789 | -0.45364 | -0.63932 | -0.5867 |
| Com_955_neg | -0.47673 | 0.609887 | -0.01253 | 0.622719 | 0.41938463 | 0.756356 | -0.50886 | -0.64822 | -0.22991 |
| Com_137_neg | -0.52173 | 0.602887 | -0.68611 | 0.440458 | 0.46926409 | 0.524144 | -0.5337 | -0.62578 | -0.61584 |
| Com_1660_neg | -0.52945 | 0.492615 | -0.34336 | 0.521198 | 0.46747016 | 0.723282 | -0.55156 | -0.56477 | -0.4156 |
| Com_1844_pos | -0.49311 | 0.541276 | -0.62893 | 0.481083 | 0.42295821 | 0.554399 | -0.47828 | -0.61658 | -0.53774 |
| Com_337_neg | -0.57693 | 0.537317 | -0.60953 | 0.519274 | 0.52347963 | 0.66988 | -0.52111 | -0.58777 | -0.62939 |
| Com_4071_pos | 0.467268 | -0.65798 | -0.0937 | -0.58694 | -0.2338151 | -0.50716 | 0.468514 | 0.61615 | -0.11011 |

Continued

| ID | Prot_B0K025 | Prot_B1H216 | Prot_B1H234 | Prot_B1WBP1 | Prot_B1WBR5 | Prot_B1WC02 | Prot_B1WC49 | Prot_B2GUY8 | Prot_B2GV65 | Prot_B2GV89 |
| --- | --- | --- | --- | --- | --- | --- | --- | --- | --- | --- |
| Com_2013_pos | 0.743875 | -0.57706 | -0.63525 | -0.07753 | -0.62058 | -0.53769 | -0.62483 | -0.02435 | -0.60689 | -0.62293 |
| Com_23145_pos | 0.622119 | -0.66387 | -0.5699 | -0.04016 | -0.52659 | -0.51394 | -0.51728 | -0.11474 | -0.55094 | -0.55908 |
| Com_3764_pos | 0.60853 | -0.69339 | -0.60837 | 0.000381 | -0.58248 | -0.53409 | -0.50704 | -0.22939 | -0.46087 | -0.50003 |
| Com_692_pos | -0.62649 | 0.325574 | 0.696469 | 0.302533 | 0.345518 | 0.517219 | 0.691635 | 0.373999 | 0.752897 | 0.757832 |
| Com_9745_pos | -0.59403 | 0.274605 | 0.56396 | 0.403031 | 0.438259 | 0.47763 | 0.728177 | 0.427512 | 0.620524 | 0.742219 |
| Com_6269_pos | 0.755739 | -0.6316 | -0.6075 | -0.0118 | -0.59623 | -0.48509 | -0.57737 | -0.08559 | -0.45994 | -0.57733 |
| Com_165_neg | -0.8224 | 0.441879 | 0.720749 | 0.138768 | 0.449716 | 0.538641 | 0.591949 | -0.15778 | 0.713075 | 0.609637 |
| Com_1512_pos | -0.63462 | 0.464001 | 0.615549 | 0.212294 | 0.249449 | 0.546243 | 0.450222 | -0.13656 | 0.662814 | 0.413086 |
| Com_1241_neg | 0.701739 | -0.62333 | -0.65245 | 0.048237 | -0.63611 | -0.42101 | -0.59869 | -0.05006 | -0.45855 | -0.62785 |
| Com_780_pos | 0.681646 | -0.70579 | -0.57297 | -0.05635 | -0.58237 | -0.56715 | -0.51045 | 0.037154 | -0.46593 | -0.47025 |
| Com_9406_pos | 0.762005 | -0.54427 | -0.67161 | 0.10978 | -0.64195 | -0.41464 | -0.60858 | -0.01584 | -0.47176 | -0.57592 |
| Com_308_pos | 0.710428 | -0.63422 | -0.66701 | 0.108218 | -0.65381 | -0.43892 | -0.5683 | 0.017246 | -0.42581 | -0.58078 |
| Com_8285_pos | -0.53516 | 0.757168 | 0.597295 | 0.043464 | 0.534168 | 0.378375 | 0.584976 | 0.126285 | 0.250737 | 0.596193 |
| Com_1214_neg | 0.646389 | -0.66334 | -0.65723 | 0.097017 | -0.64201 | -0.41388 | -0.55016 | -0.02624 | -0.40962 | -0.57408 |
| Com_3075_neg | 0.641374 | -0.63909 | -0.62606 | 0.099777 | -0.59188 | -0.32465 | -0.52644 | -0.02262 | -0.34306 | -0.58109 |
| Com_619_pos | 0.701299 | -0.67996 | -0.62165 | -0.0047 | -0.53019 | -0.64732 | -0.45012 | 0.040868 | -0.57639 | -0.42781 |
| Com_1617_neg | 0.505549 | -0.64405 | -0.56638 | 0.121295 | -0.56212 | -0.4114 | -0.46664 | -0.06774 | -0.42444 | -0.65662 |
| Com_909_pos | 0.677607 | -0.65473 | -0.65411 | 0.121338 | -0.64482 | -0.44649 | -0.53079 | 0.023442 | -0.41701 | -0.55248 |
| Com_433_neg | 0.603471 | -0.70805 | -0.60876 | 0.021005 | -0.61796 | -0.42945 | -0.50383 | 0.010946 | -0.38689 | -0.49004 |
| Com_3856_pos | 0.71528 | -0.50505 | -0.53798 | -0.09882 | -0.77398 | -0.60862 | -0.63956 | -0.19322 | -0.54294 | -0.59396 |
| Com_101_neg | -0.79787 | 0.510817 | 0.742687 | 0.152826 | 0.399188 | 0.416101 | 0.608899 | -0.31943 | 0.590574 | 0.522756 |
| Com_508_neg | 0.671868 | -0.66487 | -0.50206 | -0.16286 | -0.38123 | -0.47436 | -0.52729 | -0.18178 | -0.47875 | -0.6093 |
| Com_260_pos | 0.73857 | -0.52119 | -0.69366 | 0.204797 | -0.49498 | -0.43079 | -0.47447 | -0.02573 | -0.61461 | -0.56489 |
| Com_3273_neg | 0.651678 | -0.66603 | -0.6259 | 0.109031 | -0.61569 | -0.40198 | -0.49226 | 0.02813 | -0.39163 | -0.55742 |
| Com_1503_neg | 0.693266 | -0.66153 | -0.65148 | 0.10843 | -0.49236 | -0.445 | -0.42001 | 0.112351 | -0.51791 | -0.56426 |
| Com_295_pos | 0.669546 | -0.65139 | -0.64469 | 0.040172 | -0.64619 | -0.43952 | -0.54808 | 0.094305 | -0.42058 | -0.53201 |
| Com_2609_pos | 0.626502 | -0.68663 | -0.64362 | 0.049041 | -0.59824 | -0.42621 | -0.48402 | 0.07969 | -0.40986 | -0.45183 |
| Com_2200_neg | -0.60687 | 0.593002 | 0.63746 | -0.13239 | 0.625392 | 0.42626 | 0.382204 | 0.093568 | 0.308465 | 0.525336 |
| Com_829_neg | 0.668763 | -0.627 | -0.65453 | 0.122001 | -0.66044 | -0.37481 | -0.55438 | 0.024956 | -0.39797 | -0.57226 |
| Com_2636_neg | 0.576804 | -0.64418 | -0.62697 | 0.118368 | -0.55901 | -0.25851 | -0.51347 | -0.06556 | -0.30096 | -0.56001 |
| Com_383_pos | 0.682959 | -0.60439 | -0.46799 | -0.046 | -0.51936 | -0.67404 | -0.40815 | -0.18891 | -0.5556 | -0.44428 |
| Com_1672_neg | 0.682531 | -0.55714 | -0.57737 | 0.121568 | -0.57454 | -0.26539 | -0.50644 | 0.026218 | -0.3294 | -0.5916 |
| Com_1082_neg | -0.86297 | 0.545075 | 0.650256 | 0.276238 | 0.431076 | 0.594169 | 0.628439 | -0.00706 | 0.628555 | 0.537512 |
| Com_262_pos | 0.761535 | -0.51981 | -0.62076 | 0.224292 | -0.59222 | -0.47272 | -0.47881 | 0.033512 | -0.53674 | -0.51658 |
| Com_620_pos | -0.58398 | 0.490107 | 0.545393 | 0.190474 | 0.525393 | 0.55433 | 0.614538 | 0.219355 | 0.492618 | 0.439698 |
| Com_683_neg | 0.596962 | -0.6234 | -0.61729 | 0.123314 | -0.70657 | -0.35892 | -0.54978 | -0.04785 | -0.30221 | -0.56935 |
| Com_4633_pos | 0.726841 | -0.62432 | -0.63286 | 0.149842 | -0.53004 | -0.37406 | -0.47642 | 0.11481 | -0.46772 | -0.46558 |
| Com_2633_pos | 0.676213 | -0.62561 | -0.61831 | 0.031043 | -0.62051 | -0.45918 | -0.49247 | 0.108693 | -0.43072 | -0.53485 |
| Com_339_neg | -0.53786 | 0.771755 | 0.394553 | 0.048194 | 0.408852 | 0.379734 | 0.337375 | 0.264818 | 0.142335 | 0.276683 |
| Com_11428_pos | 0.676026 | -0.66136 | -0.70309 | -0.11524 | -0.43142 | -0.61278 | -0.42911 | -0.03245 | -0.56044 | -0.47447 |
| Com_12307_pos | 0.660455 | -0.58147 | -0.66828 | -0.07425 | -0.50408 | -0.51053 | -0.53485 | -0.02427 | -0.48483 | -0.35442 |
| Com_1827_neg | 0.636396 | -0.68172 | -0.60967 | 0.140934 | -0.59776 | -0.35908 | -0.46848 | 0.095375 | -0.3097 | -0.49142 |
| Com_2532_neg | 0.597694 | -0.47141 | -0.53713 | -0.05555 | -0.65967 | -0.40313 | -0.638 | -0.05955 | -0.51846 | -0.52959 |
| Com_1031_neg | 0.581458 | -0.67826 | -0.63368 | 0.190513 | -0.6062 | -0.37406 | -0.41861 | 0.076228 | -0.3574 | -0.46625 |
| Com_955_neg | 0.606029 | -0.63879 | -0.42821 | -0.36955 | -0.39123 | -0.45503 | -0.65624 | -0.2162 | -0.30105 | -0.42993 |
| Com_137_neg | 0.634007 | -0.66791 | -0.63123 | 0.170729 | -0.58631 | -0.35097 | -0.44589 | 0.102862 | -0.38246 | -0.53284 |
| Com_1660_neg | 0.520891 | -0.5713 | -0.48716 | -0.06719 | -0.7133 | -0.45733 | -0.59366 | -0.13988 | -0.31872 | -0.52399 |
| Com_1844_pos | 0.633666 | -0.64333 | -0.62823 | 0.117983 | -0.68765 | -0.42699 | -0.51944 | 0.099934 | -0.29962 | -0.52195 |
| Com_337_neg | 0.798182 | -0.6614 | -0.64836 | 0.017963 | -0.4874 | -0.47456 | -0.49556 | 0.141559 | -0.46508 | -0.46749 |
| Com_4071_pos | -0.42026 | 0.5346 | 0.470398 | 0.422413 | 0.373087 | 0.341046 | 0.615072 | 0.357933 | 0.191326 | 0.403513 |
